# Synthesis of Novel Chalcone-Based Phenothiazine Derivatives as Antioxidant and Anticancer Agents

**DOI:** 10.3390/molecules25194566

**Published:** 2020-10-06

**Authors:** Nourah A. Al Zahrani, Reda M. El-Shishtawy, Mahmoud M. Elaasser, Abdullah M. Asiri

**Affiliations:** 1 Chemistry Department, Faculty of Science, King Abdulaziz University, Jeddah 21589, Saudi Arabia; nourh.a.z@hotmail.com (N.A.A.Z.); aasiri2@kau.edu.sa (A.M.A.); 2 Chemistry Department, Faculty of Science, University of Jeddah, Jeddah 21959, Saudi Arabia; 3 Dyeing, Printing and Textile Auxiliaries Department, Textile Research Division, National Research Centre, Dokki, Cairo 12611, Egypt; 4 The Regional Center for Mycology and Biotechnology, Al-Azhar University, Cairo 11759, Egypt; mmelaasser@hotmail.com; 5 Center of Excellence for Advanced Materials Research, King Abdulaziz University, Jeddah 21589, Saudi Arabia

**Keywords:** chalcone, phenothiazine, antioxidant, cytotoxicity, Hep-G2, MCF-7

## Abstract

Based on reported results for the potential medicinal impact of phenothiazine core, as well as the chalcone skeleton that is widely present in many natural products, together with their reported bioactivities, the present work was aimed at combining both moieties in one molecular skeleton and to synthesize and characterize a novel series of chalone-based phenothiazine derivatives. For this purpose, 2-acetylphenothiazine was N-alkylated, followed by the Claisen-Schmidt reaction to produce the chalcones with good yield. Antioxidant activity, as evaluated by 2,2-diphenyl-1-picrylhydrazyl (DPPH) free radical scavenging, was assessed to determine if their antioxidant potential was comparable with ascorbic acid, and attributable to the phenothiazine core. Screening anticancer activities of the synthesized chalone-based phenothiazine derivatives against human breast cancer cell line MCF-7 cells, and human hepatocellular carcinoma HepG-2 cells, compared with standard drugs cisplatin and doxorubicin, was evaluated. The results revealed that compounds **4a, 4b, 4d, 4h, 4j, 4k, 4m, 4o,** and **4p** were good against human hepatocellular carcinoma HepG-2 cells, and among these compounds **4b** and **4k** were the most effective compounds, with IC_50_ values of 7.14 μg/mL and 7.6 1 μg/mL, respectively. On the other hand, compounds **4a, 4b, 4k,** and **4m** were good against human breast cancer cell line MCF-7 cells and, among these compounds, **4k** and **4b** were the most effective compounds, with IC_50_ values of 12 μg/mL and 13. 8 μg/mL, respectively. The overall results suggest that these compounds could, potentially, be further modified for the formation of more potent antioxidant and anticancer agents.

## 1. Introduction

Studies related to pathology and prevention have received significant attention, especially in regard to the effect of free radicals in causing many diseases in addition to the role of antioxidants in preventing diseases [1,2]. Under normal conditions, glutathione synthesized in the cells protects them from harmful objects and oxidative damage [3]. However, there are factors that prevent the functioning of this natural defense balance such as going through bad psychological states and exposure to pollution, radioactive environmental factors such as ultraviolet rays and the use of toxic chemicals. Smoking and malnutrition can lead to lipid peroxidation, oxidative damage to DNA and proteins, etc., which necessitates taking medication doses of antioxidants to prevent the occurrence of some chronic diseases such as many cancers, cardiovascular diseases and neurological diseases [4]. Studies have shown that increasing the production of free radicals or decreasing the efficiency of the defense systems in the human body, greatly affects the occurrence of Alzheimer’s disease. Natural antioxidants are found in foods, artificial types can be added to food to extend their expiry dates, and they can be prepared by extracting them from plant sources to be taken as concentrated nutritional supplements [5,6]. Antioxidants can be obtained in limited proportions from natural sources. Moreover, most natural antioxidants have restricted therapeutic success due to their poor solubility in both aqueous or lipid systems, which prevents them from reaching the target cells [7,8].

Inspired by nature, considerable attention has been paid to developing synthetic and semisynthetic antioxidant-containing natural cores [9,10]. In this context, synthesis of the chalcone skeleton, which is present in many natural products with extensive bioactivities [11], was attempted in conjunction with the phenothiazine core, the first lead pharmacophore of the 20th century [12]. Several phenothiazine derivatives with various structural motifs have been recently reported and evaluated as anticancer and antioxidant agents [13,14,15,16,17,18,19,20,21]. For the treatment of Alzheimer’s disease, some N-alkyl and phenothiazine amide derivatives were synthesized, and their efficacy, as well their selectivity as inhibitors for cholinesterase, were investigated [22]. Chalcone compounds are among the most important fundamental categories of natural products that are abundant within tea leaves, fruits and vegetables, and are of great interest because of their pharmacological effectiveness in treating many diseases. The term chalcone characterizes structures of the 1,3-diphenylprop-2-en-1-one system. Chemically, chalcones can be prepared conveniently by means of the Claisen–Schmidt condensation or aldol condensation procedure [11]. 

Structures with chalcone skeletons have a variety of biological activities, e.g., anti-inflammatory [23], anticancer [24], antimicrobial [25], antiviral [26], analgesic [27], antiplatelet [28], antioxidant [29], antitubercular [30], antihyperglycemic [31], inhibition of chemical mediator production [32], inhibition of leukotriene B4 [33], inhibition of tyrosine and inhibition of aldose reductase activities [34]. Chalcone compounds also have important antioxidant capacity and can form phenoxyl radicals with peroxide reductases. Phenoxyl radicals are highly effective and produce reactive oxygen species (ROS), e.g., the superoxide anion (O^2−^), hydrogen peroxide (H_2_O_2_), and the hydroxyl radical (HO^•^) [35]. Chalcones derivatives are extremely vigorous free radical scavengers and DNA-damaging factors [36]. Several chalcone-based compounds have been approved for clinical use, such as metochalcone and sofalcone [37]. A few studies have been reported on chalcone-based phenothiazine [38,39,40]. In this context, a series of different N-substituted rhodamines were synthesized and evaluated for their chemotherapeutic effectiveness on antileukemia cell line K562 [41]. Other phenothiazine-based chalcones have been synthesized and evaluated as inhibitors for acetylcholine esterase as an indication for the treatment of Alzheimer’s disease [42] and as antitumor agents [43].

2-acetylphenothiazine has been reported as a superoxide ion inhibitor [44]. Since this compound is amenable for N-alkylation, it was envisioned that introducing a long chain would furnish the precursor of chalcone-based phenothiazine, which would result in a series of different compounds. Thus, N-alkylation of 2-acetylphenothiazine by phase transfer catalysis (PTC), followed by Claisen-Schmidt condensation with 16 different aryl aldehydes, produced the corresponding novel chalcone-based phenothiazine derivatives, and their bioactivities as antioxidant and anticancer agents were evaluated.

## 2. Results and Discussion

### 2.1. Chemistry

**Scheme 1:**
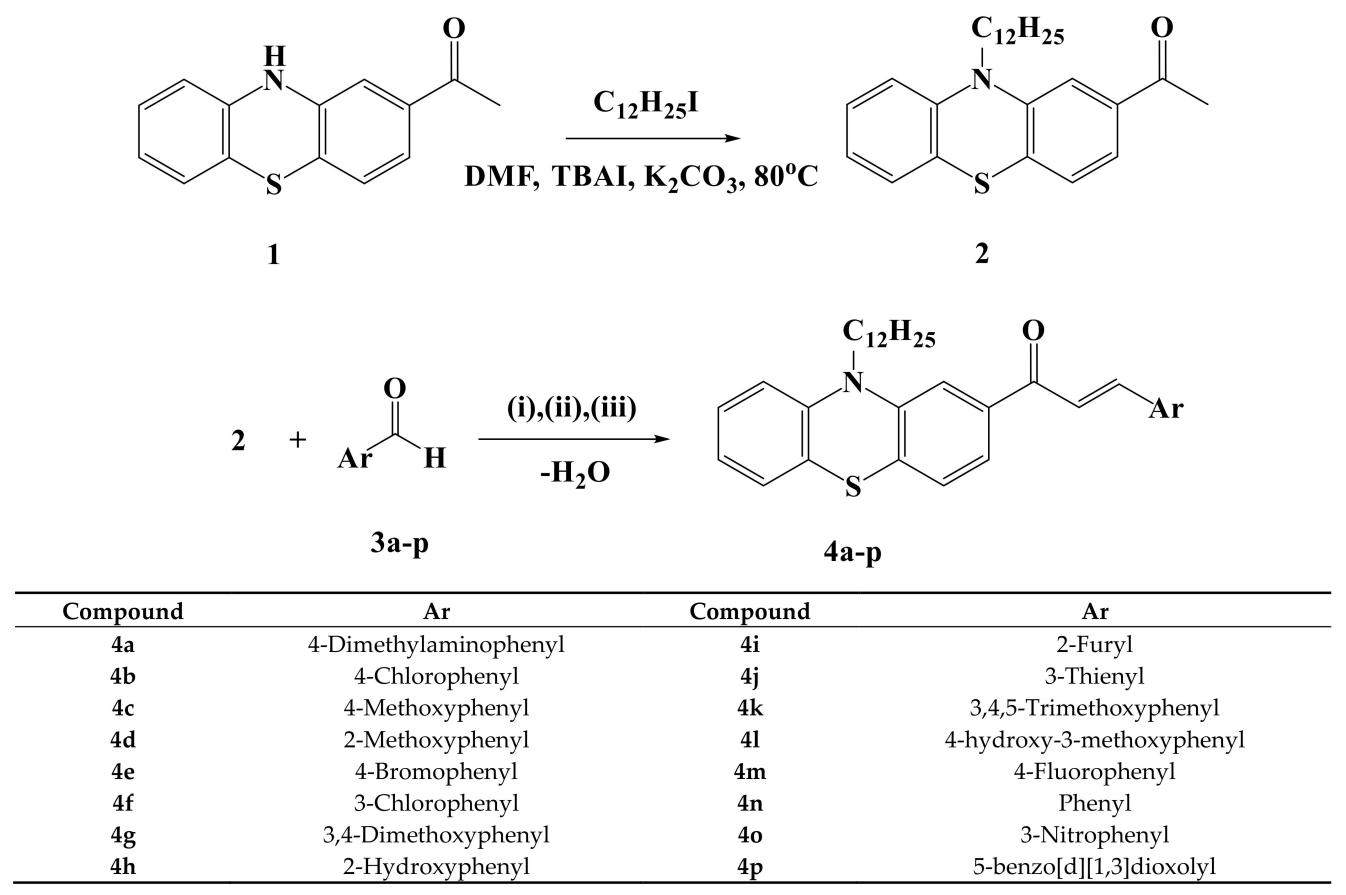
Synthetic routes for chalcone derivatives (**4a–4p**); i = 5% alcoholic NaOH/room temperature/overnight; ii = ethanol-piperidine/reflux/overnight; iii = methanol/50% aqueous KOH/ room temperature/overnight.

A series of chalcone-based phenothiazine derivatives was synthesized, as shown in Scheme 1. For this purpose, 2-acetylphenothiazine (1) was N-alkylated with dodecyl iodide via S_N_2 reaction by phase transfer catalysis using tert-butyl ammonium iodide (TBAI) as the catalyst in a heterogeneous basic medium to afford the corresponding product (2) in a good yield. Dodecyl iodide was selected as the alkylating agent to introduce a long chain, as it would improve the solubility of the final product in common organic solvents and lead to better absorption of chalcone by living cells.

Three base-catalyzed methods of the Claisen-Schmidt reaction were adopted for the synthesis of chalcones. The reaction proceeds via enolate addition on aryl carbonyl, followed by the elimination of water to produce the chalcones [45]. It is noteworthy that alcoholic NaOH and/or KOH were used for the synthesis of chalcones from compound **2** with various aryl aldehydes, except **4h** and **4l** because their aryl aldehydes precursors contain phenolic groups that would react with compound **2** in alcoholic piperidine. The suitability of piperidine in these cases was attributed to the increased nucleophilicity of enolate over the phenolate anion, leading to the formation of the chalcones **4h** and **4l**. The synthesized chalcones **4a**–**4p** were fully characterized, and their chemical structures shown in Scheme 1 were elucidated. The chalcones synthesized in the present study were considered *E* isomers because the range of their coupling constants (*J*) was 15–17 Hz. A signal belonging to the N-CH_2_ group on the phenothiazine ring was observed at around 3.9 ppm as a triplet. Using ^13^C nuclear magnetic resonance (^13^C-NMR), a signal indicating the carbonyl group was seen around 190 ppm. The Supplementary file (S1–S85) contains the NMR, the attenuated total reflectance–Fourier transform infrared (ATR–FTIR) and high resolution mass spectrometry (HRMS) charts for the synthesized compounds.

### 2.2. Antioxidant Activity

**Scheme 2:**
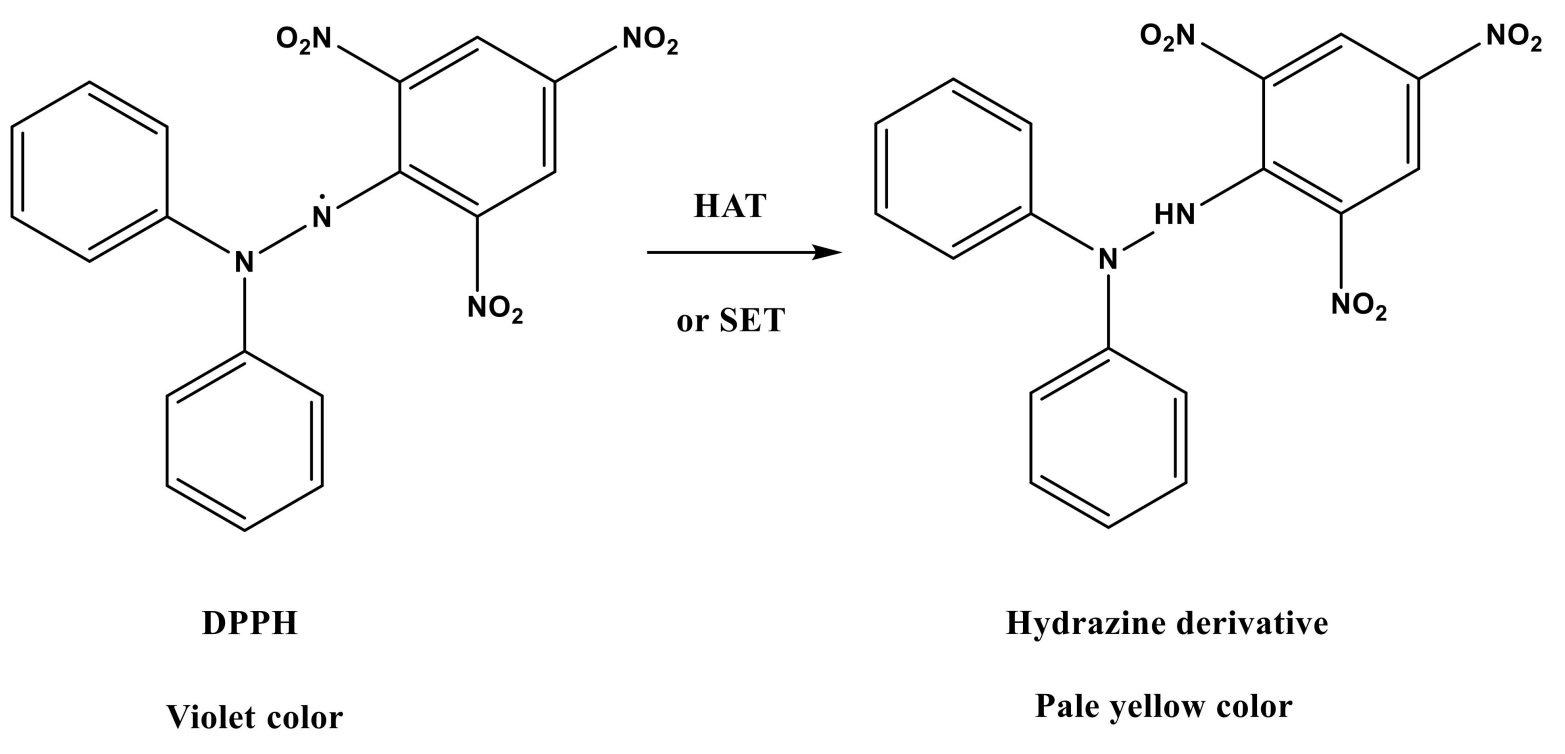
Antioxidant activity of a compound by transfer of the hydrogen atom (HAT) or single electron (SET).

The use of DPPH (2,2-diphenyl-1-(2,4,6-trinitrophenyl)hydrazyl), an indicator that has a maximum absorption in methanol at 516 nm due its stable radical, for the evaluation of antioxidant activity in vitro, has been reported as viable radical scavenging method [46,47,48,49]. The antioxidant activity of a compound depends on either its ability to transfer the hydrogen atom (HAT) or a single electron (SET) to DPPH to form a pale-yellow hydrazine derivative, as shown in Scheme 2.

**Figure 1:**
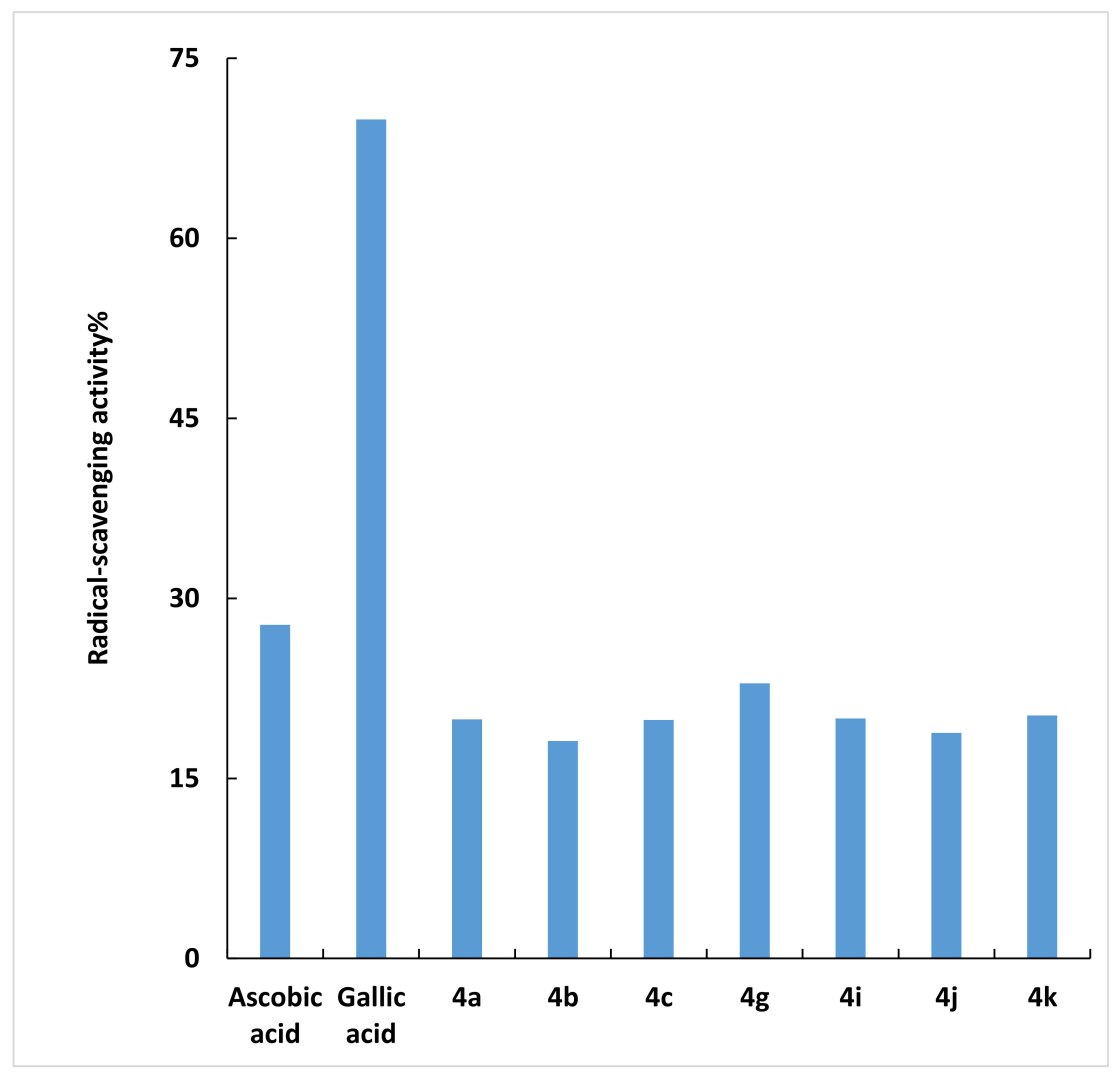
2,2-diphenyl-1-picrylhydrazyl (DPPH) scavenging activity of 1 µM of compounds **4a–4c**, **4g**, **4i–4k**, ascorbic acid and gallic acid.

Antioxidant activity was evaluated for compounds **4a–4c**, **4g** and **4i–4k** compared with gallic acid and ascorbic acid, at the same concentration, as shown in Figure 1. It is clearly observed that the antioxidant activities of the tested compounds were lower than that of gallic acid but closer to that of ascorbic acid. It is also observed that the activity of the tested compounds as antioxidants was slightly dependent on the aryl ring, indicating that the potency of this class of compounds as antioxidants is mainly due to the phenothiazine ring. This result is in accordance with similar chemical antioxidant activity for the nonalkylated phenothiazine-based chalcone reported earlier [38]. It has been reported that the antioxidant activity of phenothiazine and its derivatives is due to the formation of a stable radical cation that becomes stabilized over a large conjugative moiety, and to the change of the phenothiazine butterfly structure to a planar one. In this context, the pharmacological activity of phenothiazine core as an anticancer agent has been attributed to radical stability [50]. It is worthy of mentioning that chemical antioxidant activity was determined by a simple assay, and in vitro antioxidant activity would better reflect a closer value to the in vivo activity [51,52].

### 2.3. Cytotoxic Activity

**Figure 2:**
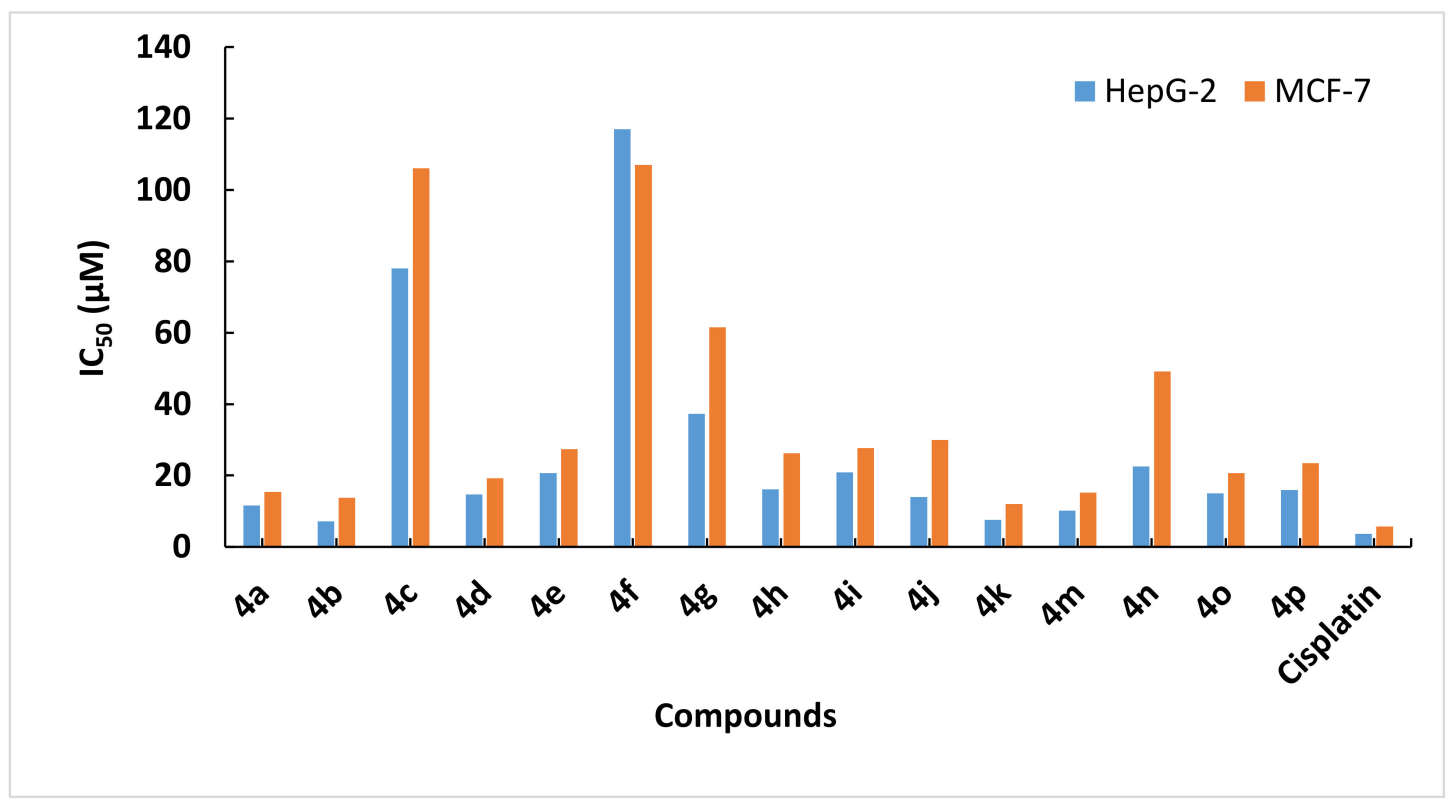
Cytotoxic activities of tested compounds against human hepatocellular carcinoma (HepG-2) and human breast cancer cell line MCF-7 cells.

The in vitro growth inhibitory activity of the chalcone-based phenothiazine derivatives was evaluated against two carcinoma cell lines (human breast cancer cell line MCF-7 cells and human hepatocellular carcinoma HepG-2 cells) and compared with the well-known anticancer standard drugs cisplatin and doxorubicin under the same conditions following the MTT (methylthiazol-tetrazolium) colorimetric assay. The obtained data in triplicate for each concentration were plotted and tabulated as shown in Figure 2, Table 1 and S86, S87. IC_50_ values (concentrations of tested compounds required to kill 50% of cells) were determined. The results shown in S86 and S87 indicate that all compounds had concentration-dependent inhibitory activity to both cancer cell lines and were comparable with cisplatin. Figure 2 shows that the synthesized chalcones revealed structurally-dependent potential anticancer activities compared with cisplatin. Table 1 summarizes the IC_50_ values for all tested compounds. It is clear that compounds **4a, 4b, 4d, 4h, 4j, 4k, 4m, 4o** and **4p** were effective against human hepatocellular carcinoma HepG-2 cells and, among these compounds, **4b** and **4k** were the most effective compounds with IC_50_ values of 7.14 μg/mL and 7.6 μg/mL, respectively. On the other hand, compounds **4a, 4b, 4k,** and **4m** were effective against the human breast cancer cell line MCF-7 cells and, among these compounds, **4k** and **4b** were the most effective with IC_50_ values of 12 μg/mL and 13. 8 μg/mL, respectively. Interestingly, compound **4f** was the least effective among all tested compounds against both cancer cell lines. This compound is an isomer of the most potent compound **4b** in which the chlorine atom is in the 4-position whereas, for **4f**, the chlorine atom is in 3-position in the aryl ring. This result indicates that structural isomerization has a different impact on the cytotoxicity of the tested compounds. Other tested compounds had from moderate to weak activity against both cancer cell lines.

The analysis was performed using the methylthiazol-tetrazolium (MTT) assay after 24 h of incubation. Values are shown as mean ±SD of three replicates.

## 3. Experimental

### 3.1. General

All solvents were purchased from Sigma Aldrich and Fisher and used directly without further purification. 2-acetylphenothiazine was purchased from Sigma Aldrich (St. Louis, MO, USA). All other chemicals used were of analytical grade and were used without further purification. Chromatographic separations were carried out on silica gel (60–120 mesh). ^1^H and ^13^C-NMR spectra were recorded in DMSO-d_6_ or CDCl_3_ on a Bruker Avance (Billerica, MA, USA) 400 M Hz or 850 M Hz and 100 M Hz or 213 M Hz spectrometer, respectively. The reported chemical shifts were against TMS. The attenuated total reflectance–Fourier transform infrared (ATR–FTIR) spectrum was performed on a PerkinElmer spectrum 100 FT-IR spectrometer (Shelton, CT 06484, USA). Mass spectra were recorded on a positive ion mode on a Bruker Impact II, LC-MS/MSm (Billerica, MA, USA). Melting points were determined in open capillary tubes in Stuart scientific melting point apparatus SMP3 and were uncorrected.

### 3.2. 1-(10-Dodecylphenothiazin-2-yl)Ethan-1-one (***2***)

A mixture of 2-acetylphenothiazine (1) (1.4 g, 6 mmol), dodecyl iodide (5.3 g, 1.8 mmol), potassium hydroxide 40% (20 mL) and tert-butyl ammonium iodide (TBAI) (0.66 g, 1.8 mmol) in 20 mL toluene was heated with stirring at 80 °C for 1 h. The mixture was then cooled and mixed with 100 mL of water, followed by ethyl acetate extraction (4 × 60 mL). The organic layer was washed with a saturated aqueous solution of ammonium chloride and then water. After drying the organic layer with sodium sulfate anhydrous and evaporation under reduced pressure, a brown oil of the product was obtained. The product was purified by column chromatography (eluent: petroleum ether: ethyl acetate 98:2) on silica gel to afford the pure oily product in 59% yield. ^1^H-NMR (850 M Hz, DMSO-*d_6_*) δ (ppm): 0.84 (t, 3H, *J* = 7.65 Hz, CH_3_), 1.20–1.27 (m, 16H, 8CH_2_), 1.37 (quintet, 2H, *J* = 7.65 Hz, CH_2_), 1.67 (quintet, 2H, *J* = 7.65 Hz, CH_2_), 2.55 (s, 3H, COCH_3_), 3.91 (t, 2H, *J* = 6.8 Hz, N-CH_2_), 6.96 (td, 1H, *J* = 6.8, 0.85 H, Ar-H), 7.03 (dd, 1H, *J* = 7.65, 0.85 Hz, Ar-H), 7.14 (dd, 1H, *J* = 7.65, 1.7 Hz, Ar-H), 7.22 (td, 1H, *J* = 6.8, 1.7 Hz, Ar-H), 7.27 (d, 1H, *J* = 8.5, Ar-H), 7.40 (sd, 1H, *J* = 1.7 Hz, Ar-H), 7.53 (dd, 1H, *J* = 7.65, 1.7 Hz, Ar-H). ^13^C-NMR (213 M Hz, DMSO-*d_6_*) δ (ppm): 14.43, 22.55, 26.22, 26.36, 26.44, 28.86, 29.13, 29.20, 29.27, 29.40, 29.42, 31.73, 46.84, 113.91, 115.72, 122.70, 122.95, 123.61, 126.96, 127.41, 127.62, 132.04, 136.24, 144.57, 145.57, 197.45. IR *ν* cm^−1^: 2922, 2852 (aliphatic C-H), 1681 (C=O), 1591, 1558(C=C).

### 3.3. General Procedure for the Synthesis of Chalcone-Based Phenothiazine Derivatives 

#### 3.3.1. Compounds **4a–g, 4j, 4k, 4m, 4n** and **4p** (i)

A mixture of compound **2** (0.41 g, 1 mmol) and the appropriate aryl aldehyde (2 mmol) was stirred overnight in 15 mL of 5% alcoholic NaOH at room temperature. TLC monitored the completion of the reaction. Upon completion of the reaction, water was added, and the so-formed precipitate was filtered off, washed with water, ethanol, and purified with column chromatography using 8:2 petroleum ether: ethyl acetate as eluent to produce the pure product. In the case of an oil product, the usual work-up of organic phase extraction was followed using ethyl acetate. After removing the solvent in vacuo and following the same protocol of purification by column chromatography, the pure oily product was obtained. 

#### 3.3.2. Compounds **4h** and **4i** (ii)

A mixture of compound **2** (0.41 g, 1 mmol) and the appropriate aryl aldehyde (2 mmol) was refluxed with stirring overnight in 30 mL ethanol that contained five drops of piperidine. TLC monitored the completion of the reaction. Upon completion of the reaction, the oily product was extracted, as usual, and purified by column chromatography as above to give the pure product. 

#### 3.3.3. Compounds **4i** and **4o** (iii)

A mixture of compound **2** (0.41 g, 1 mmol), 0.5 mL methanol, and 1 mL of an aqueous KOH (50%) was stirred for 1 h, and then the appropriate aryl aldehyde (2 mmol) dissolved in 0.5 mL methanol was added. The reaction mixture was stirred at room temperature overnight. Methanol was removed in vacuo, and another 1 mL of aqueous KOH (50%) was added to the mixture. TLC monitored the completion of the reaction. After 1 h, the mixture was neutralized with ice-cooled aqueous HCl (10%), then water was added and the mixture finally extracted with ethyl acetate (4 × 25 mL), followed by the usual work-up, to produce a solid product that was further purified by column chromatography, as above, to give the pure product.

*3-(4-(dimethylamino)phenyl)-1-(10-dodecylphenothiazin-2-yl)prop-2-en-1-one *(**4a**)Orange solid, 80% yield (0.42 g, 1.0 mmol), m.p 77 °C; **^1^**H-NMR (850 M Hz, CDCl_3_) δ(ppm): 0.88 (t, 3H, *J* = 7.65 Hz, CH_3_), 1.20–1.33 (m, 16H, CH_2_), 1.44 (quintet, 2H, *J* = 7.65 Hz, CH_2_), 1.82 (quintet, 2H, *J* = 6.8 Hz, CH_2_), 3.05 (s, 6H, O-CH_3_), 3.91 (t, 2H, *J* = 7.65 Hz, N-CH_2_), 6.72 (d, 1H, *J* = 6.97 Hz, Ar-H), 6.87 (dd, 1H, *J* = 8.5, 0.85 Hz, Ar-H), 6.92 (td, 1H, *J* = 6.8, 0.85 Hz, Ar-H), 7.11 (dd, 1H, *J* = 7.65, 0.85 Hz, Ar-H), 7.16 (td,1H, *J* = 7.65, 1.7 Hz, Ar-H), 7.19 (d,1H, *J* = 7.56 Hz, Ar-H), 7.29 (d,1H, *J* = 16.15 Hz, =CH), 7.49 (d,1H, *J* = 1.7 Hz, Ar-H), 7.53(dd, 1H, *J* = 8.5, 1.7 Hz, Ar-H), 7.55 (d,2H, *J* = 8.5 Hz, ArH), 7.78 (d, 1H, *J* = 15.3 Hz, =CH). ^13^C-NMR (213 M Hz, CDCl_3_) δ (ppm)**:** 14.14, 22.70, 26.77, 26.97, 29.28, 29.36, 29.54, 29.58, 29.65, 29.71, 31.93, 40.28, 47.57, 112.04, 114.61, 115.67, 116.86, 122.52, 122.55, 123.77, 126.80, 127.38, 127.53, 130.42, 130.76, 138.13, 144.71, 145.55, 189.71. IR *ν* cm^−1^: 2920, 2849 (aliphatic C-H), 1734 (C=O), 1646 (olefinic C=C), 1571, 1549 (Ar C=C). HRMS (ESI): *m/z* calculated for C_35_H_45_N_2_OS 541.3253 [M]^+^, found 541.3247.

*3-(4-chlorophenyl)-1-(10-dodecylphenothiazin-2-yl)prop-2-en-1-one *(**4b**) Orange oil, 50% (0.2 g, 1.0 mmol); ^1^H-NMR (850 M Hz, CDCl_3_) δ (ppm): 0.86 (t, 3H, *J* = 7.65 Hz, CH_3_), 1.20–1.33 (m, 16H, CH_2_), 1.44 (quintet, 2H, *J* = 7.65 Hz, CH_2_), 1.82 (quintet, 2H, *J* = 7.65 Hz, CH_2_), 3.91 (t, 2H, *J* = 6.8 Hz, N-CH_2_), 6.87 (dd, 1H, *J* = 7.65, 0.85 Hz, Ar-H), 6.93 (td, 1H, *J* = 7.65, 0.85 Hz, Ar-H), 7.11 (dd, 1H, *J* = 7.65, 0.85 Hz, Ar-H), 7.17 (td, 1H, *J* = 7.65, 1.7 Hz, Ar-H), 7.2 (d, 1H, *J* = 7.65, Ar-H), 7.31 (dd, 2H, *J* = 8.5, 1.7 Hz, ArH), 7.45 (d, 1H, *J* = 15.3 Hz, =CH), 7.48 (d,1H, *J* = 1.7 Hz, Ar-H), 7.52 (dd, 1H, *J* = 8.5, 1.7 Hz, ArH), 7.57 (dd, 2H, *J* = 6.8, 1.7 Hz, Ar-H), 7.75 (d, 1H, *J* = 16.15 Hz, =CH). ^13^C-NMR (213 M Hz, CDCl_3_) δ(ppm)**:** 14.10, 22.63, 26.70, 26.94, 29.22, 31.73, 47.60, 114.47, 115.73, 122.31, 122.72, 123.51, 126.91, 127.41, 127.65, 129.27, 129.58, 132.03, 133.42, 136.44, 137.08, 143.14, 144.48, 145.75, 189.31. IR *ν* cm^−1^: 3061 (H-C=C), 2926, 2853 (aliphatic C-H),1659 (C=O), 1605 (olefinic C=C),1589 (Ar C=C). HRMS (ESI): *m/z* calculated for C_33_H_39_ClNOS 532. 2441 [M + 1]^+^, found 532.2435.

*1-(10-dodecylphenothiazin-2-yl)-3-(4-methoxyphenyl)prop-2-en-1-one *(**4c**) Orange oil, 60% (0.25 g; 1.0 mmol); ^1^H-NMR (850 M Hz, CDCl_3_) δ(ppm): 0.87 (t, 3H, *J* = 6.8 Hz, CH_3_), 1.20–1.33 (m, 16H, CH_2_), 1.44 (quintet, 2H, *J* = 7.65 Hz, CH_2_), 1.82 (quintet, 2H, *J* = 6.8 Hz, CH_2_), 3.86 (s, 3H, O-CH_3_), 3.91 (t, 2H,*J* = 6.8 Hz, N-CH_2_), 6.87 (d, 1H, *J* = 7.65 Hz, Ar-H), 6.92 (dd, 1H, *J* = 7.65, 0.85 Hz, Ar-H), 6.94 (dd, 2H, *J* = 6.8, 1.7 Hz, Ar-H), 7.11 (dd, 1H, *J* = 7.65, 1.7 Hz, Ar-H), 7.17(td,1H, *J* = 7.65, 0.85 Hz Ar-H), 7.19 (d, 1H, *J* = 8.5 Hz, Ar-H), 7.36 (d, 1H, *J* = 16.15 Hz, =CH), 7.48 (d, 1H, *J* = 1.7 Hz, Ar-H), 7.53 (dd,1H, *J* = 7.65, 1.7 Hz, Ar-H), 7.59 (dd, 2H,*J* = 6.8, 1.7 Hz, Ar-H), 7.78 (d, 1H,*J* = 15.3 Hz, =CH). ^13^C-NMR (213 M Hz, CDCl_3_) δ(ppm): 14.13, 22.7, 26.73, 26.95, 29.26, 29.35, 29.52, 29.56, 29.63, 31.92, 47.58, 55.43, 114.43, 114.55, 115.70, 119.59, 122.63, 123.64, 126.86, 127.39, 127.59, 127.65, 130.23, 131.41, 137.57, 144.55, 144.59, 145.65, 161.69, 189.66. IR *ν* cm^−1^: 3065 (H-C=C), 2924, 2851 (aliphatic C-H), 1736 (C=O), 1656 (olefinic C=C), 1591 (Ar C=C), 1171(C-O). HRMS (ESI): *m/z* calculated for C_34_H_42_NO_2_S 528.2936 [M + 1]^+^, found 528.2931.

*1-(10-dodecylphenothiazin-2-yl)-3-(2-methoxyphenyl)prop-2-en-1-one *(**4d**) Orange oil, 60% (0.25 g, 1.0 mmol); ^1^H-NMR (850 M Hz, CDCl_3_) δ(ppm): 0.87 (t, 3H, *J* = 7.65 Hz, CH_3_), 1.20–1.33 (m, 16H, CH_2_), 1.44 (quintet, 2H, *J* = 7.65 Hz, CH_2_), 1.82 (quintet, 2H, *J* = 7.65 Hz, CH_2_), 3.90 (t, 2H, *J* = 6.8 Hz, N-CH_2_), 3.92(s, 3H, O-CH_3_), 6.87 (dd, 1H, *J* = 7.65, 0.85 Hz, Ar-H), 6.92 (td, 1H, *J* = 6.8, 0.85 Hz, Ar-H), 6.94 (d, 1H, *J* = 8.5 Hz, Ar-H), 6.99 (t, 1H, *J* = 6.8 Hz, Ar-H), 7.11 (dd, 1H, *J* = 7.65, 0.85 Hz, Ar-H), 7.13 (td, 1H, *J* = 8.5, 1.7 Hz, Ar-H), 7.19 (d, 1H, *J* = 7.65 Hz, Ar-H), 7.38 (td, 1H, *J* = 9.35, 1.7 Hz, Ar-H), 7.49 (d, 1H, *J* = 1.7 Hz, Ar-H), 7.53 (dd, 1H, *J* = 8.5, 1.7 Hz, Ar-H), 7.56 (d, 1H, *J* = 16.15 Hz, =CH), 7.63 (dd, 1H, *J* = 7.65, 1.7 Hz, Ar-H), 8.10 (d, 1H, *J* = 16.15 Hz, =CH). ^13^C-NMR (213 M Hz, CDCl_3_) δ(ppm): 14.13, 22.68, 26.71, 26.95, 29.24, 29.33, 29.51, 29.55, 29.62, 31.91, 47.57, 54.65, 55.54, 111.22, 114.65, 115.66, 120.75, 122.60,122.78, 123.62, 123.96, 126.85, 127.37, 127.57, 129.20, 131.29, 131.74, 137.58, 140.25, 144.61, 145.56, 158.79, 190.31, 198.84. IR ν cm^−1^: 2924, 2850 (aliphatic C-H), 1743 (C=O), 1657 (olefinic C=C), 1598 (Ar C=C). HRMS (ESI): *m/z* calculated for C_34_H_42_NO_2_S 528.2936 [M + 1]^+^, found 528.2931.

*3-(4-bromophenyl)-1-(10-dodecylphenothiazin-2-yl)prop-2-en-1-one (***4e**) Orange oil, 80% (0.24 g, 1.0 mmol); ^1^H-NMR (850 M Hz, CDCl_3_) δ(ppm): 0.87 (t, 3H, *J* = 6.8 Hz, CH_3_), 1.20–1.33 (m, 16H, CH_2_), 1.44 (quintet, 2H, *J* = 7.65 Hz, CH_2_), 1.87 (quintet, 2H, *J* = 7.65 Hz, CH_2_), 3.91 (t, 2H, *J* = 7.65 Hz, N-CH_2_), 6.87 (d, 1H, *J* = 7.65 Hz, Ar-H), 6.93 (td, 1H, *J* = 7.65, 0.85 Hz, Ar-H), 7.11 (dd, 1H, *J* = 7.65, 1.7 Hz, Ar-H), 7.17 (td, 1H, *J* = 7.65, 1.7 Hz, Ar-H), 7.2 (d, 1H, *J* = 8.5 Hz, Ar-H), 7.47 (d, 1H, *J* = 15.3 Hz, =CH), 7.48 (d, 1H, *J* = 1.7 Hz, Ar-H), 7.49 (d, 2H, *J* = 7.65 Hz, Ar-H), 7.52 (dd, 1H, *J* = 8.5, 1.7 Hz, Ar-H), 7.56 (d, 2H, *J* = 8.5 Hz, Ar-H), 7.73 (d, 1H, *J* = 16.15 Hz, =CH). ^13^C-NMR (213 M Hz, CDCl_3_) δ(ppm): 14.12, 22.69, 26.68, 26.93, 29.24, 29.34, 29.51, 29.55, 29.62, 31.91, 47.59, 114.45, 115.72, 122.38,122.71, 123.50, 124.79, 126.90, 127.39, 127.64, 129.77, 129.91, 132.04, 132.22, 132.37, 133.84, 137.05, 143.17, 144.46, 145.74, 189.28. IR *ν* cm^−1^: 2925, 2852 (aliphatic C-H), 1661 (C=O), 1604 (olefinic C=C), 1590 (Ar C=C). HRMS (ESI): *m/z* calculated for C_33_H_39_BrNOS 576.1936 [M + 1]^+^, found 576.1930.

*3-(3-chlorophenyl)-1-(10-dodecylphenothiazin-2-yl)prop-2-en-1-one *(**4f**) Orange oil, 80% (0.24 g, 1.0 mmol); ^1^H-NMR (850 M Hz, CDCl_3_) δ(ppm): 0.90 (t, 3H, *J* = 7.65 Hz, CH_3_), 1.23–1.36 (m, 16H, CH_2_), 1.47 (quintet, 2H, *J* = 7.65 Hz, CH_2_), 1.84 (quintet, 2H, *J* = 7.65 Hz, CH_2_), 3.93 (t, 2H, *J* = 7.65 Hz, N-CH_2_), 6.91 (d, 1H, *J* = 7.65 Hz, Ar-H), 6.96 (t, 1H, *J* = 7.65 Hz, Ar-H), 7.13 (dd, 1H, *J* = 7.65, 1.7 Hz, Ar-H), 7.19 (td, 1H, *J* = 8.5, 1.7 Hz, Ar-H), 7.23(d, 1H, *J* = 8.5 Hz, Ar-H), 7.38 (m, 1H, Ar-H), 7.41 (dt, 1H, *J* = 8.5, 1.7 Hz, Ar-H), 7.45 (d, 1H, *J* = 15.3 Hz, =CH), 7.52 (d, 2H, *J* = 7.65 Hz, Ar-H), 7.56 (d, 1H, *J* = 7.65 Hz, Ar-H), 7.65 (t, 1H, *J* = 1.7 Hz, Ar-H), 7.75(d, 1H, *J* = 15.3 Hz, =CH). ^13^C-NMR (213 M Hz, CDCl_3_) δ(ppm): 14.14, 22.71, 26.69, 26.94, 29.25, 29.36, 29.52, 29.55, 29.63, 29.71, 31.93, 47.66, 114.50, 115.78, 122.75,122.80, 123.05, 123.51, 126.79, 126.94, 127.41,127.66,127.90,130.22, 130.34, 132.18, 135.00, 136.78, 136.96, 142. 87, 144.42, 145.72, 189.12. IR ν cm^−1^: 3063 (H-C=C), 2923, 2849 (aliphatic C-H),1739 (C=O), 1660 (olefinic C=C), 1605, 1592 (Ar C=C). HRMS (ESI): *m/z* calculated for C_33_H_39_ClNOS 532. 2441 [M + 1]^+^, found 532.2435.

*3-(3,4-dimethoxyphenyl)-1-(10-dodecylphenothiazin-2-yl)prop-2-en-1-one *(**4g**) Orange oil, 44% (0.27 g, 1.0 mmol); ^1^H-NMR (850 M Hz, CDCl_3_) δ(ppm): 0.88 (t, 3H, *J* = 6.8 Hz, CH_3_), 1.20–1.33 (m, 16H, CH_2_), 1.44 (quintet, 2H, *J* = 8.5 Hz, CH_2_), 1.81 (quintet, 2H, *J* = 7.65 Hz, CH_2_), 3.91 (t, 2H, *J* = 6.8 Hz, N-CH_2_), 3.94 (s, 3H, O-CH_3_), 3.96 (s, 3H, O-CH_3_), 6.88 (dd, 1H, *J* = 8.5, 0.85 Hz, Ar-H), 6.91 (d, 1H, *J* = 7.65 Hz, Ar-H), 6.93 (td, 1H, *J* = 7.65, 0.85 Hz, Ar-H), 7.11 (dd, 1H, *J* = 8.5, 1.7 Hz Ar-H), 7.15 (d, 1H, J = 1.7 Hz, Ar-H), 7.17 (td, 1H, *J* = 8.5, 1.7 Hz, Ar-H), 7.20 (d, 1H, *J* = 7.65 Hz, Ar-H), 7.24(dd,1H, *J* = 8.5, 2.6 Hz, Ar-H), 7.33 (d,1H, *J* = 16.15 Hz,=CH), 7.49(d, 1H, *J* = 1.7 Hz, Ar-H), 7.53 (dd, 1H, *J* = 8.5, 1.7 Hz, Ar-H), 7.75 (d, 1H, *J* = 15.3 Hz, =CH). ^13^C-NMR (213 M Hz, CDCl_3_) δ(ppm): 14.09, 14.12, 22.62, 26.72, 26.94,29.21, 29.36, 29.70, 31.72, 31.92, 47.58, 56.02, 110.14, 111.12, 114.55, 115.70, 119.92, 122.65, 123.09, 126.82, 127.39, 127.59, 127.89, 131.45, 144.87, 145.68, 149.25, 151.44, 189.74. IR *ν* cm^−1^: 3199 (H-C=C), 2922, 2853 (aliphatic C-H), 1727 (C=O), 1660 (olefinic C=C), 1592 (Ar C=C). HRMS (ESI): *m/z* calculated for C_35_H_44_NO_3_S 558.3042 [M + 1]^+^, found 558.3036.

*1-(10-dodecylphenothiazin-2-yl)-3-(2-hydroxyphenyl)prop-2-en-1-one *(**4h**) Red oil, 60% (0.24 g, 1.0 mmol); ^1^H-NMR (850 M Hz, CDCl_3_) δ(ppm): 0.87 (t, 3H, *J* = 6.8 Hz, CH_3_), 1.20-1.33 (m, 16H, CH_2_), 1.44 (quintet, 2H, *J* = 7.65 Hz, CH_2_), 1.82 (quintet, 2H, *J* = 6.8 Hz, CH_2_), 3.90 (t, 2H, *J* = 7.65 Hz, N-CH_2_), 6.87 (d, 2H, *J* = 7.65 Hz, Ar-H), 6.92 (td, 1H, *J* = 6.8, 0.85 Hz, Ar-H), 6.97 (t, 1H, *J* = 7.65 Hz, Ar-H), 7.11 (dd, 1H, *J* = 7.65, 0.85 Hz, Ar-H), 7.16 (td, 1H, *J* = 6.8, 0.85 Hz, Ar-H), 7.19 (d, 1H, *J* = 8.5 Hz, Ar-H), 7.26 (m, 2H, Ar-H), 7.49 (sd, 1H, *J* = 1.7 Hz, Ar-H), 7.55 (dd, 1H, *J* = 7.65, 1.7 Hz, Ar-H), 7.59 (dd, 1H, *J* = 7.65 Hz, 0.85 Hz, Ar-H), 7.63 (d, 1H, *J* = 15.32 Hz, =CH), 8.10 (d, 1H, *J* = 16.15 Hz, =CH). ^13^C-NMR (213 M Hz, CDCl_3_) δ(ppm): 14.12, 22.69, 26.70, 26.94, 29.25, 29.34, 29.52, 29.56, 29.62, 29.70, 31.91, 47.58, 114.63, 115.68, 116.53, 121.01, 122.25, 122.63, 122.82, 122.87, 123.56, 126.87, 127.37, 127.59, 129.56, 131.62, 131.68, 137.38, 140.30, 144.55, 145.60, 155.54, 190.57. IR *ν* cm^−1^: 3061 (H-C=C), 2925, 2851 (aliphatic C-H),1645(C=O), 1591 (olefinic C=C), 1554 (Ar C=C). HRMS (ESI): *m/z* calculated for C_33_H_40_NO_2_S 514.2780 [M + 1]^+^, found 514.2774.

*1-(10-dodecylphenothiazin-2-yl)-3-(furan-2-yl)prop-2-en-1-one (***4i**) Deep orange oil, 90% (0.47 g, 1.0 mmol); ^1^H-NMR (850 M Hz, CDCl_3_) δ(ppm): 0.90 (t, 3H, *J* = 6.8 Hz, CH_3_), 1.22–1.35 (m, 16H, CH_2_), 1.47 (quintet, 2H, *J* = 7.65 Hz, CH_2_), 1.84 (quintet, 2H, *J* = 6.8 Hz, CH_2_), 3.93 (t, 2H, *J* = 7.65 Hz, N-CH_2_), 6.54 (dd, 1H, *J* = 3.4 Hz, 1.7 Hz, Ar-H), 6.74 (d, 1H, *J* = 3.4 Hz, Ar-H), 6.89 (dd, 1H, *J* = 7.65, 0.85 Hz, Ar-H), 6.94 (td, 1H, *J* = 7.65, 0.85 Hz, Ar-H), 7.13 (dd, 1H, *J* = 7.65, 1.7 Hz, Ar-H), 7.18 (td, 1H, *J* = 6.8, 0.85 Hz, Ar-H), 7.19 (sd, 1H, *J* = 0.85 Hz, Ar-H), 7.43 (d, 1H, *J* = 15.3 Hz, =CH), 7.52 (sd, 1H, *J* = 1.7 Hz, Ar-H), 7.55 (sd, 1H, *J* = 1.7 Hz, Ar-H), 7.58 (dd, 1H, *J* = 7.65, 1.7 Hz,Ar-H), 7.61 (d, 1H, *J* = 15.3 Hz, =CH). ^13^C-NMR (213 M Hz, CDCl_3_) δ(ppm): 14.14, 22.71, 26.71, 26.93, 29.25, 29.36, 29.53, 29.64, 31.93, 47.58, 112.71, 114.44, 115.72, 116.25, 119.13, 122.65, 122.70, 123,61, 126.92, 127.39, 127.60, 130.53, 131.76, 137.24, 144.55, 144.92, 145.66, 151.72, 188.88. IR *ν* cm^−1^: 3060 (H-C=C), 2922, 2852 (aliphatic C-H), 1658 (C=O), 1600 (olefinic C=C), 1552 (Ar C=C). HRMS (ESI): *m/z* calculated for C_31_H_38_NO_2_S 488.2623 [M + 1]^+^, found 488.2618.

*1-(10-dodecylphenothiazin-2-yl)-3-(thiophen-3-yl)prop-2-en-1-one (***4j**) Orange oil, 90% (0.47g, 1.0 mmol); ^1^H-NMR (850 M Hz, CDCl_3_) δ(ppm): 0.89 (t, 3H, *J* = 7.65 Hz, CH_3_), 1.22–1.35 (m, 16H, CH_2_), 1.47 (quintet, 2H, *J* = 7.65 Hz, CH_2_), 1.84 (quintet, 2H, *J* = 6.8 Hz, CH_2_), 3.93 (t, 2H, *J* = 7.65 Hz, N-CH_2_), 6.89 (d, 1H, *J* = 8.5 Hz, Ar-H), 6.95 (t, 1H, *J* = 6.8 Hz, Ar-H), 7.12 (m, 2H, Ar-H), 7.19 (t, 1H, *J* = 8.5 Hz, Ar-H), 7.22 (dd, 1H, *J* = 8.5, 0.85 Hz, Ar-H), 7.31 (d, 1H, *J* = 14.45 Hz, =CH), 7.38 (d, 1H, *J* = 3.4 Hz, Ar-H), 7.44 (d, 1H, *J* = 4.25 Hz, Ar-H), 7.50 (s, 1H, Ar-H), 7.54 (d, 1H, *J* = 7.65 Hz, Ar-H), 7.9 (d, 1H, *J* = 15.3 Hz, =CH). ^13^C-NMR (213 M Hz, CDCl_3_) δ(ppm): 14.15, 22.71, 26.71, 26.95, 29.26, 29.36, 29.53, 29.57, 29.64, 31.93, 47.61, 114.44, 115.71, 120.62, 122.63, 122.67, 123.55, 126.90, 127.39, 127.62, 128.38, 128.79, 131.74, 132.06, 137.06, 137.18, 140.45, 144.52, 145.65,188.93. IR *ν* cm^−1^: (H-C=C), 2923,2851 (aliphatic C-H),1654(C=O), 1587 (olefinic C=C), 1557(Ar C=C). HRMS (ESI): *m/z* calculated for C_31_H_38_NOS_2_ 504.2395 [M + 1]^+^, found 504.2389.

*1-(10-dodecylphenothiazin-2-yl)-3-(3,4,5-trimethoxyphenyl)prop-2-en-1-on *(**4k**) Red oil, 80% (0.24 g; 1.0 mmol); ^1^H-NMR (850 M Hz, CDCl_3_) δ(ppm): 0.89 (t, 3H, *J* = 7.65 Hz, CH_3_), 1.21–1.34 (m, 16H, CH_2_), 1.46 (quintet, 2H, *J* = 7.65 Hz, CH_2_), 1.84 (quintet, 2H, *J* = 6.8 Hz, CH_2_), 3.93 (t, 2H, *J* = 7.65 Hz, N-CH_2_), 3.93 (s, 3H.O-CH_3_), 3.95 (s, 6H. O-CH_3_), 6.88 (s,2H, Ar-H), 6.90 (d, 1H, *J* = 8.5 Hz, Ar-H), 6.95 (t, 1H, *J* = 7.65 Hz, Ar-H), 7.14 (dd, 1H, *J* = 7.65, 1.7 Hz, Ar-H), 7.19 (td, 1H, *J* = 8.5, 0.85 Hz, Ar-H), 7.23 (d, 1H, *J* = 7.65 Hz, Ar-H), 7.87 (d, 1H, *J* = 16.15 Hz, =CH), 7.51 (s,1H, Ar-H), 7.55 (dd, 1H, *J* = 7.65, 0.85 Hz, Ar-H), 7.73 (d, 1H, *J* = 15.3 Hz, =CH). ^13^C-NMR (213 M Hz, CDCl_3_) δ(ppm): 14.14, 22.70, 26.71, 26.93, 29.24, 29.35, 29.52, 29.55, 29.63, 31.92, 47.63, 61.03, 105.67, 114.58, 115.78, 121.33, 122.74, 123.60, 126.85, 127.41, 127.64, 130.40, 131.74, 137.34, 140.47, 144.48, 144.87, 145.71, 153.50, 189.64. IR *ν* cm^−1^: 3063 (H-C=C), 2923, 2851 (aliphatic C-H), 1659(C=O), 1581 (olefinic C=C), 1505 (Ar C=C), 1126 (C-O). HRMS (ESI): *m/z* calculated for C_36_H_46_NO_4_S 588.3148 [M + 1]^+^, found 588.3142.

*1-(10-dodecylphenothiazin-2-yl)-3-(4-hydroxy-3-methoxyphenyl)prop-2-en-1-one *(**4l**) Orange oil, 60% (0.24 g; 1.0 mmol); ^1^H-NMR (850 M Hz, CDCl_3_) δ(ppm): 0.89 (t, 3H, *J* = 7.65 Hz, CH_3_), 1.21–1.34 (m, 16H, CH_2_), 1.46 (quintet, 2H, *J* = 7.65 Hz, CH_2_), 1.85 (quintet, 2H, *J* = 6.8 Hz, CH_2_), 3.93 (t, 2H, *J* = 6.8 Hz, N-CH_2_), 3.96 (s, 3H.O-CH_3_), 6.27(s, 1H.O-H), 6.89 (m, 2H, Ar-H), 6.92 (m, 1H, Ar-H), 6.94 (m, 1H, Ar-H), 7.13 (dd, 1H, *J* = 7.65, 0.85 Hz, Ar-H), 7.19 (m,1H, Ar-H), 7.22 (dd, 1H, *J* = 7.65, 2.55 Hz, Ar-H), 7.52(dd, 1H, *J* = 8.5, 1.7 Hz,Ar-H), 7.57 (td, 1H, *J* = 7.56, 1.7 Hz, Ar-H), 7.72 (d, 1H, *J* = 16 Hz, H-C=C), 8.03 (d,1H, *J* = 16.15 Hz, H-C=C). ^13^C-NMR (213 M Hz, CDCl_3_) δ(ppm): 14.14, 22.70, 26.73, 26.97, 29.27, 29.36, 29.53, 29.64, 31.93, 47.62, 56.25, 111.92, 112.34, 114.61, 114.70, 115.68, 115.71, 119.75, 120.51, 121.34, 121.83, 122.09, 122.87, 123.49, 126.88, 127.39, 127.58, 137.57, 138.19, 139.94, 144.63, 145.54, 145.82, 146.85. IR *ν* cm^−1^: 2924, 2854 (aliphatic C-H), 1721(C=O), 1656 (olefinic C=C), 1589 (Ar C=C), 1267(C-O). HRMS (ESI): *m/z* calculated for C_34_H_42_NO_3_S 544.2885 [M + 1]^+^, found 544.2880.

*1-(10-dodecylphenothiazin-2-yl)-3-(4-fluorophenyl)prop-2-en-1-on (***4m**) Shiny Orange oil, 80% (0.24 g; 1.0 mmol); ^1^H-NMR (850 M Hz, CDCl_3_) δ(ppm): 0.87 (t, 3H, *J* = 6.8 Hz, CH_3_), 1.20–1.33 (m, 16H, CH_2_), 1.44 (quintet, 2H, *J* = 7.65 Hz, CH_2_), 1.81 (quintet, 2H, *J* = 7.65 Hz, CH_2_), 3.89 (t, 2H, *J* = 6.8 Hz, N-CH_2_), 6.87 (d, 1H, *J* = 8.5 Hz, Ar-H), 7.11 (t, 3H, *J* = 8.5 Hz, Ar-H), 7.17 (td, 1H, *J* = 7.65, 1.7 Hz, Ar-H), 7.19 (d, 1H, *J* = 8.5 Hz Ar-H), 7.40 (d, 1H, *J* = 15.3 Hz, =CH), 7.48 (sd, 1H, *J* = 1.7 Hz, Ar-H), 7.52 (dd, 1H, *J* = 7.65, 1.7 Hz, Ar-H), 7.76 (d, 1H, *J* = 16.15 Hz, =CH). ^13^C-NMR (213 M Hz, CDCl_3_) δ(ppm): 14.12, 22.68, 26.68, 26.93, 29.24, 29.34, 29.51, 29.54, 29.61, 31.91, 47.58, 114.47, 115.71, 116.08, 116.18, 121.56, 122.67, 122.68, 123.52, 126.88, 127.38, 127.62, 130.30, 130.34, 131.16, 131.17, 131.87, 137.16, 143.30, 144.48, 145.71, 163.46, 189.33. IR *ν* cm^−1^: 3067 (H-C=C), 2919,2849 (aliphatic C-H), 1661 (C=O), 1587 (olefinic C=C), 1508 (Ar C=C). HRMS (ESI): *m/z* calculated for C_33_H_39_FNOS 516.2736 [M + 1]^+^, found 516.2731.

*1-(10-dodecylphenothiazin-2-yl)-3-phenylprop-2-en-1-one *(**4n**) Orange oil, 100% (0.1 g; 1.0 mmol); ^1^H-NMR (850 M Hz, CDCl_3_) δ(ppm): 0.87 (t, 3H, *J* = 6.8 Hz, CH_3_), 1.20–1.33 (m, 16H, CH_2_), 1.44 (quintet, 2H, *J* = 6.8 Hz, CH_2_), 1.82 (quintet, 2H, *J* = 7.65 Hz, CH_2_), 3.91 (t, 2H, *J* = 6.8 Hz, N-CH_2_), 6.88 (dd, 1H, *J* = 8.5, 0.85 Hz, Ar-H), 6.93 (td, 1H, *J* = 7.65, 0.85 Hz, Ar-H), 7.11 (dd, 1H, *J* = 7.65, 1.7 Hz, Ar-H), 7.17 (td, 1H, *J* = 8.5, 1.7 Hz, Ar-H), 7.2 (d, 1H, *J* = 8.5 Hz, Ar-H), 7.42 (m, 3H, Ar-H), 7.48 (d, 1H, *J* = 17 Hz, =CH), 7.49 (s, 1H, Ar-H), 7.54 (dd, 1H, *J* = 7.65, 1.7 Hz, Ar-H), 7.64 (m, 2H, Ar-H), 7.80 (d, 1H, *J* = 17 Hz, =CH). ^13^C-NMR (213 M Hz, CDCl_3_) δ(ppm): 14.13, 22.69, 26.70, 26.93, 29.24, 29.34, 29.51, 29.55, 29.62, 31.91, 47.59, 114.53, 115.71, 121.90, 122.67, 122.73, 123.56, 126.89, 127.39, 127.61, 128.44, 128.97, 130.54, 131.77, 134.91, 137.26, 144.53, 144.67, 145.68, 189.62. IR *ν* cm^−1^: 3062 (H-C=C), 2922, 2851 (aliphatic C-H), 1741 (C=O), 1662 (olefinic C=C), 1602 (Ar C=C). HRMS (ESI): *m/z* calculated for C_33_H_40_NOS 498.2831 [M + 1]^+^, found 498.2825.

*1-(10-dodecylphenothiazin-2-yl)-3-(3-nitrophenyl)prop-2-en-1-one (***4o**) Dark red solid mp.115 °C, 60% (0.07 g; 1.0 mmol) ^1^H-NMR (400 M Hz, DMSO-*d_6_*) δ(ppm): 0.88 (t, 3H, *J* = 7.6 Hz, CH_3_), 1.20–1.33 (m, 16H, CH_2_), 1.42 (quintet, 2H, *J* = 6.8 Hz, CH_2_), 1.72 (quintet, 2H, *J* = 7.65 Hz, CH_2_), 3.98 (t, 2H, *J* = 6.8 Hz, N-CH_2_), 6.99 (t, 1H, *J* = 7.2 Hz, Ar-H), 7.07 (t, 1H, *J* = 7.65 Hz, Ar-H), 7.18 (dd, 1H, *J* = 7.65, 1.6 Hz, Ar-H), 7.25 (td, 1H, *J* = 7.6, 1.6 Hz, Ar-H), 7.35 (d, 1H, *J* = 8 Hz, Ar-H), 7.58 (sd, 1H, *J* = 1.6 Hz, Ar-H), 7.77 (t, 1H, *J* = 8 Hz, Ar-H), 7.86 (d, d, 1H, *J* = 15.6 Hz, =CH), 7.87 (d, 1H, *J* = 7.6 Hz, Ar-H), 8.12 (d, 1H, *J* = 15.6 Hz, =CH), 8.29 (dd, 1H, *J* = 9.2 Hz, 1.2 Hz, Ar-H), 8.36 (d, 1H, *J* = 8 Hz, Ar-H), 8.78(s,1H, Ar-H). ^13^C-NMR (100 M Hz, DMSO-*d_6_*) δ(ppm): 86, 21.97, 25.86, 26.01, 28.41, 28.51, 31.00, 46.61, 114.45, 116.15, 122.30, 122.82, 123.07, 123.38, 124.65, 124.86, 127.05, 127.15, 128.00, 130.34, 130.96, 134.99, 136.62, 141.37, 143.96, 144.91, 148.42, 188.30. IR *ν* cm^−1^: 3064 (H-C=C), 2919, 2853 (aliphatic C-H), 1655 (C=O), 1592 (olefinic C=C),1558 (Ar C=C). HRMS (ESI): *m/z* calculated for C_33_H_39_N_2_O_3_S 543.2681 [M + 1]^+^, found 543.2676.

*3-(benzo[d][1,3]dioxol-5-yl)-1-(10-dodecyl-10H-phenothiazin-2-yl)prop-2-en-1-one *(**4p**) Orange oil, 60% (0.26 g; 1.0 mmol); ^1^H-NMR (850 M Hz, CDCl_3_) δ(ppm): 0.89 (t, 3H, *J* = 7.65 Hz, CH_3_), 1.23–1.36 (m, 16H, CH_2_), 1.46 (quintet, 2H, *J* = 7.65 Hz, CH_2_), 1.84 (quintet, 2H, *J* = 7.65 Hz, CH_2_), 3.92 (t, 2H, *J* = 6.8 Hz, N-CH_2_), 6.05 (s, 2H,-O-CH_2_-O), 6.87 (d, 1H, *J* = 7.65 Hz, Ar-H), 6.92 (d, 1H, *J* = 8.5 Hz, Ar-H), 6.95 (t, 1H, *J* = 7.65, Ar-H), 7.14 (td, 2H, *J* = 7.65, 1.7 Hz, Ar-H), 7.1 (m, 2H, Ar-H), 7.22 (d, 1H, *J* = 8.5 Hz, Ar-H), 7.34 (d, 1H, *J* = 15.3, Hz, H-C=C), 7.52 (s, 1H, Ar-H), 7.55 (d, 1H, *J* = 8.5 Hz, Ar-H), 7.75 (d, 1H.*J* = 15.3 Hz, H-C=C). ^13^C-NMR (213 M Hz, CDCl_3_) δ(ppm): 14.14, 22.70, 26.71, 26.91, 26.94, 29.26, 29.35, 29.52, 29.56, 39.63, 31.92, 47.72, 101.56, 106.66, 108.70, 114.65, 115.84, 119.89, 122.71, 122.75, 123.67, 125.25, 126.90, 127.40 127.62, 129.39, 131.58, 137.47, 144.44, 144.55, 145.54, 148.43, 149.94,189.43. IR *ν* cm^−1^: 3067 (H-C=C), 2922, 2853 (aliphatic C-H), 1659 (C=O), 1592 (olefinic C=C),1556 (Ar C=C), 1237 (C-O). HRMS (ESI): *m/z* calculated for C_34_H_40_NO_3_S 542.2729 [M + 1]^+^, found 542.2723.

### 3.4. Antioxidant Activity

The antioxidant activity of chalcones **4a**, **4b**, **4c**, **4g**, **4i**, **4j**, and **4k** was measured spectrophotometrically from the discoloration of ethanolic solution of 2,2-diphenyl-1-picrylhydrazyl (DPPH) at the lambda maximum of its color (516 nm) [53]. Due to the low solubility of the synthesized chalcones in ethanol, a stock solution was made for each compound in DMSO so that 1 mL of each solution was mixed with 9 mL ethanolic solution of DPPH to form a 10 mL mixture (80 µM of DPPH and 1 µM). These solutions were kept in the dark for 30 min. Similarly, a 10 mL solution of 80 µM of DPPH was kept in the dark for 30 min. After that, the absorbance was measured against the blank sample at 516 nm. The discoloration percent as a measurement of antioxidant activity was calculated by the following Equation (1):

(1) $$\%\;\mathrm{Antioxidant}\;\mathrm{activity}=\left(\frac{\mathrm{control}\;\mathrm{absorbance}-\mathrm{sample}\;\mathrm{absorbance}}{\mathrm{control}\;\mathrm{absorbance}}\right)\times 100\%$$

Ascorbic acid and gallic acid were used standard antioxidants for comparison. Data are means ± standard deviations of triplicate experiments.

### 3.5. Cytotoxic Assay

Mammalian cell lines MCF-7 cells (human breast cancer cell line) and HepG-2 (human hepatocellular carcinoma) were obtained from the American Type Culture Collection (ATCC, Rockville, MD, USA). Chemicals used: dimethyl sulfoxide (DMSO), MTT and trypan blue dye were purchased from Sigma (St. Louis, MO, USA). Fetal bovine serum, DMEM, RPMI-1640, HEPES buffer solution, L-glutamine, gentamycin and 0.25% trypsin-EDTA were purchased from Lonza (Verviers, Belgium). Cell line propagation: the cells were grown on RPMI-1640 medium supplemented with 10% inactivated fetal calf serum and 50 µg/mL gentamycin. The cells were maintained at 37 °C in a humidified atmosphere with 5% CO_2_ and were subcultured two to three times a week. 

Cytotoxicity evaluation using viability assay: for antitumor assays, the tumor cell lines were suspended in medium at a concentration of 5 × 10^4^ cells/well in Corning^®^ 96-well tissue culture plates, then incubated for 24 h. The tested compounds were then added into 96-well plates (three replicates) to achieve twelve concentrations for each compound (concentration ranges from 0–1000 µg/mL). Six vehicle controls, with media or 0.5% DMSO, were run for each 96 well plate. After incubating for 24 h, the numbers of viable cells were determined by the MTT test. Briefly, the media was removed from the 96 well plate and replaced with 100 µL of fresh culture RPMI 1640 medium without phenol red then 10 µL of the 12 mM MTT stock solution (5 mg of MTT in 1 mL of PBS) was added to each well including the untreated controls. The 96 well plates were then incubated at 37 °C and 5% CO_2_ for 4 h. An 85 µL aliquot of the media was removed from the wells, and 50 µL of DMSO was added to each well and mixed thoroughly with the pipette and incubated at 37 °C for 10 min. Then, the optical density was measured at 590 nm with a microplate reader (SunRise, TECAN, Inc., USA) to determine the number of viable cells, and the percentage of viability was calculated as [(ODt/ODc)] × 100% where ODt is the mean optical density of wells treated with the tested sample and ODc is the mean optical density of untreated cells. The relation between surviving cells and drug concentration was plotted to get the survival curve of each tumor cell line after treatment with the specified compound. The 50% inhibitory concentration (IC_50_), the concentration required to cause toxic effects in 50% of intact cells, was estimated from graphic plots of the dose response curve for each concentration using Graphpad Prism software (San Diego, CA. USA) [54,55].

## 4. Conclusions

A new series of chalcone-based phenothiazine derivatives were synthesized and fully characterized. The antioxidant activity of this class of compounds was evaluated based on DPPH radical scavenging to reveal comparable activities with ascorbic acid owing mainly to the phenothiazine core. The cytotoxic activity of the synthesized compounds against Hep-G2 and MCF-7 cancer cell lines was evaluated and compared with the standard drugs cisplatin and doxorubicin. Compounds **4b** and **4k** were most effective against both cancer cell lines. The overall results suggest that these compounds could, potentially, be further modified to produce more potent antioxidant and anticancer agents. 

## Figures and Tables

**Table 1:** The in vitro inhibitory activity expressed as 50% inhibitory concentration values (μg/mL) of tested compounds against HepG-2 and MCF-7 cancer cell lines.

Tested Compounds	IC_50_ (µg/mL)	
	HepG-2	MCF-7
**4a**	11.6 ± 0.2	15.4 ± 0.3
**4b**	7.14 ± 0.3	13.8 ± 0.4
**4c**	78 ± 0.9	106 ± 1.2
**4d**	14.7 ± 0.2	19.2 ± 0.3
**4e**	20.7 ± 0.3	27.4 ± 0.5
**4f**	117 ± 1.2	107 ± 1.3
**4g**	37.3 ± 0.5	61.5 ± 0.7
**4h**	16.1 ± 0.1	26.2 ± 0.4
**4i**	20.9 ± 0.4	27.7 ± 0.3
**4j**	14 ± 0.3	30 ± 0.7
**4k**	7.6 ± 0.2	12 ± 0.2
**4m**	10.2 ± 0.3	15.2 ± 0.4
**4n**	22.5 ± 0.5	49.1 ± 0.9
**4o**	15 ± 0.2	20.7 ± 0.3
**4p**	15.9 ± 0.4	23.5 ± 0.3
**Cisplatin**	3.67 ± 0.2	5.71 ± 0.3
**Doxorubicin**	0.36 ± 0.04	0.35 ± 0.03

## Supplementary Materials

The following are available online. The Supplementary file (S1–S85) contains the NMR, ATR-FTIR, and HRMS charts for the synthesized compounds.
