# Supplementary material for: Synthesis of Novel Chalcone-Based Phenothiazine Derivatives as Antioxidant and Anticancer Agents
Source: Molecules. 2020 Oct 6;25(19):4566. doi: 10.3390/molecules25194566 (PMC7583060; doi:10.3390/molecules25194566)
Supplement: Supplementary file 1 [file molecules-25-04566-s001.pdf]

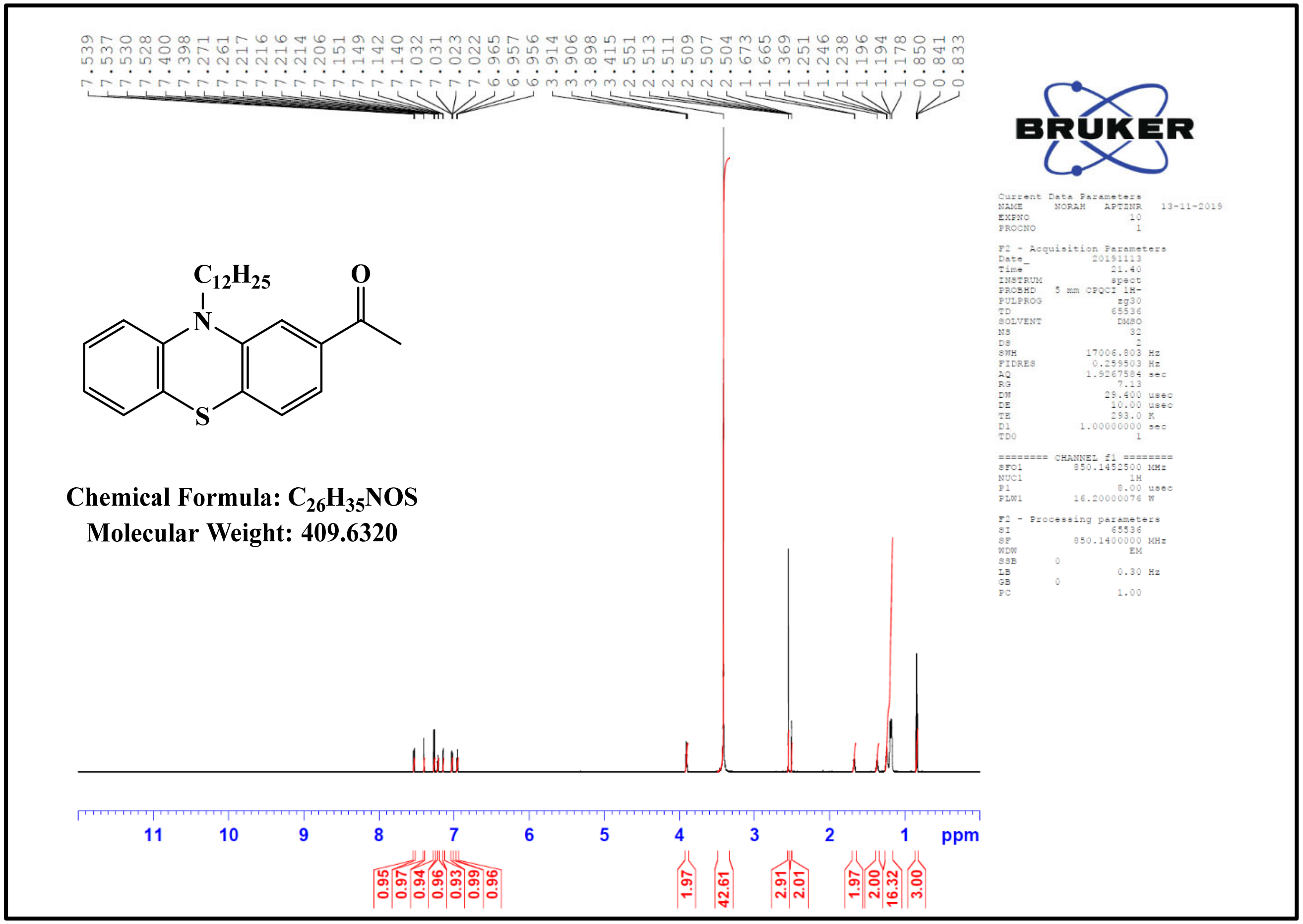

S1. 1-(10-dodecylphenothiazin-2-yl**)ethan-1-one**

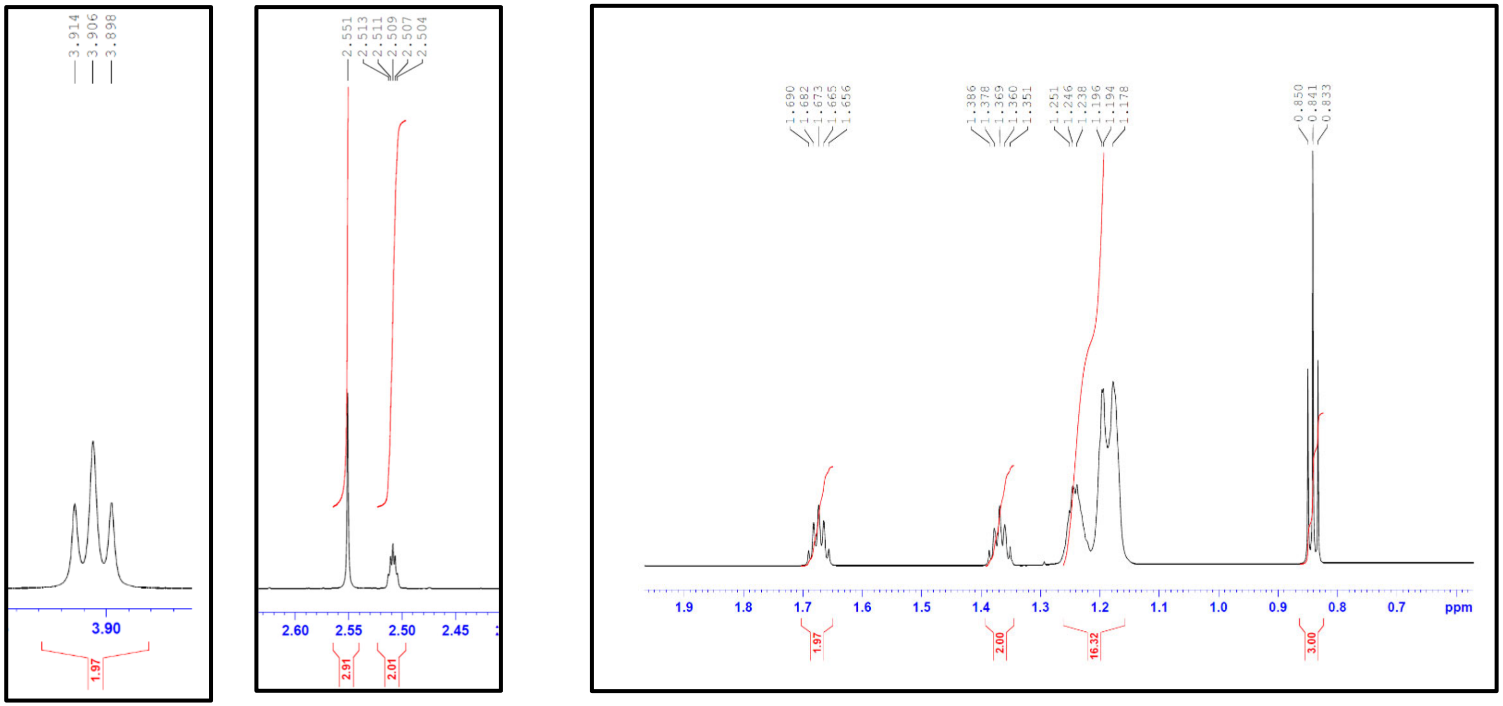

S2. 1-(10-dodecylphenothiazin-2-yl**)ethan-1-one**

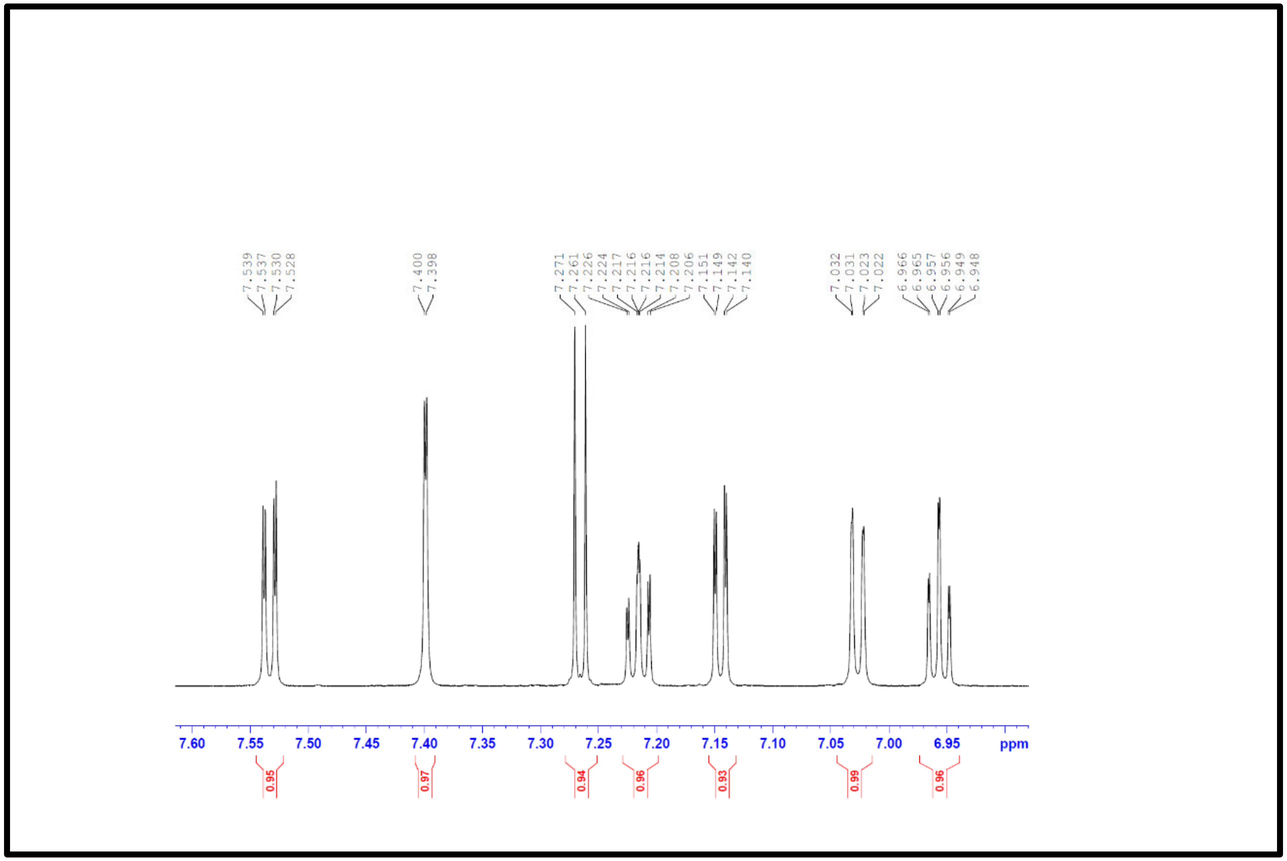

S3. 1-(10-dodecylphenothiazin-2-yl**)ethan-1-one**

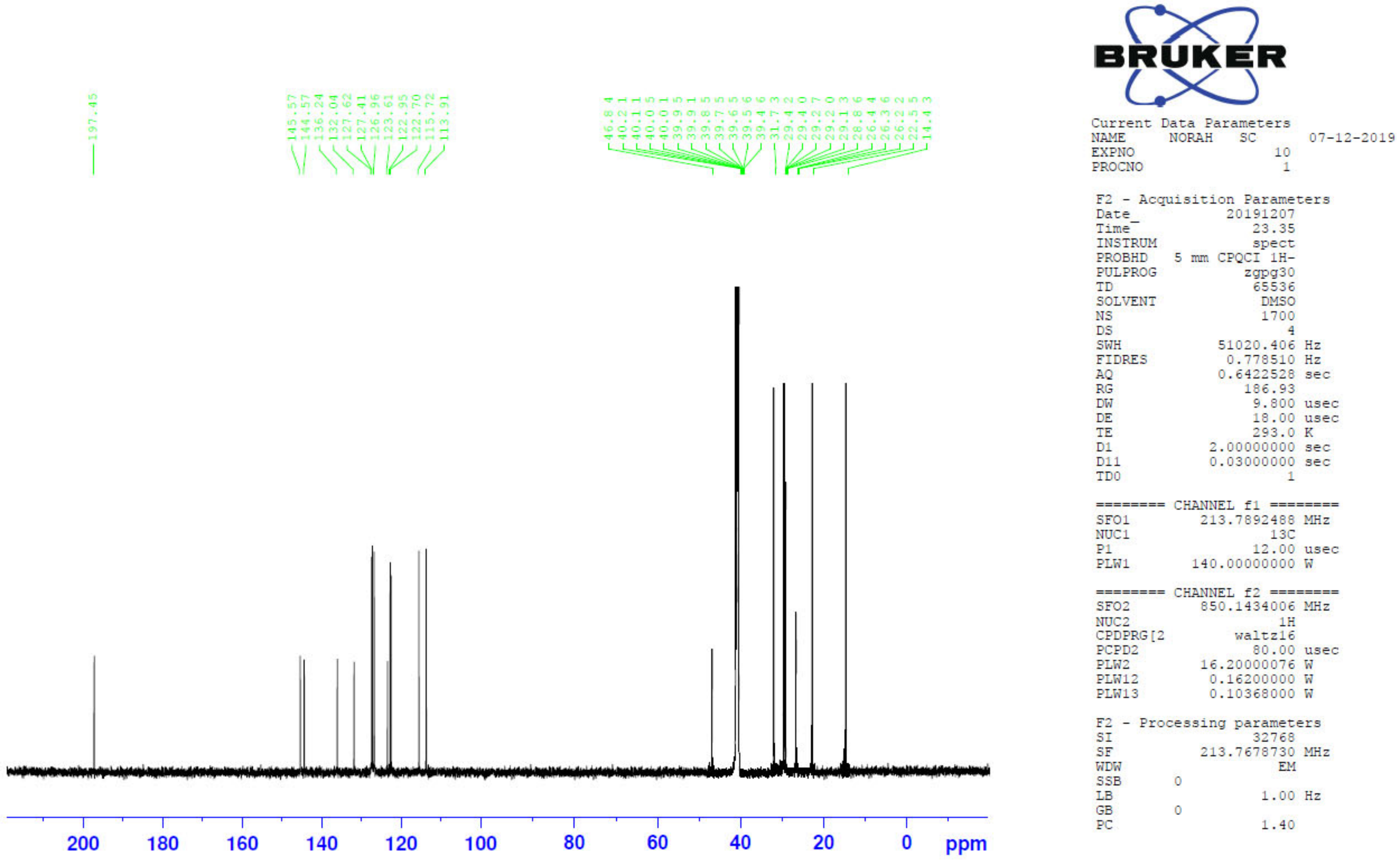

S4. 1-(10-dodecylphenothiazin-2-yl**)ethan-1-one**

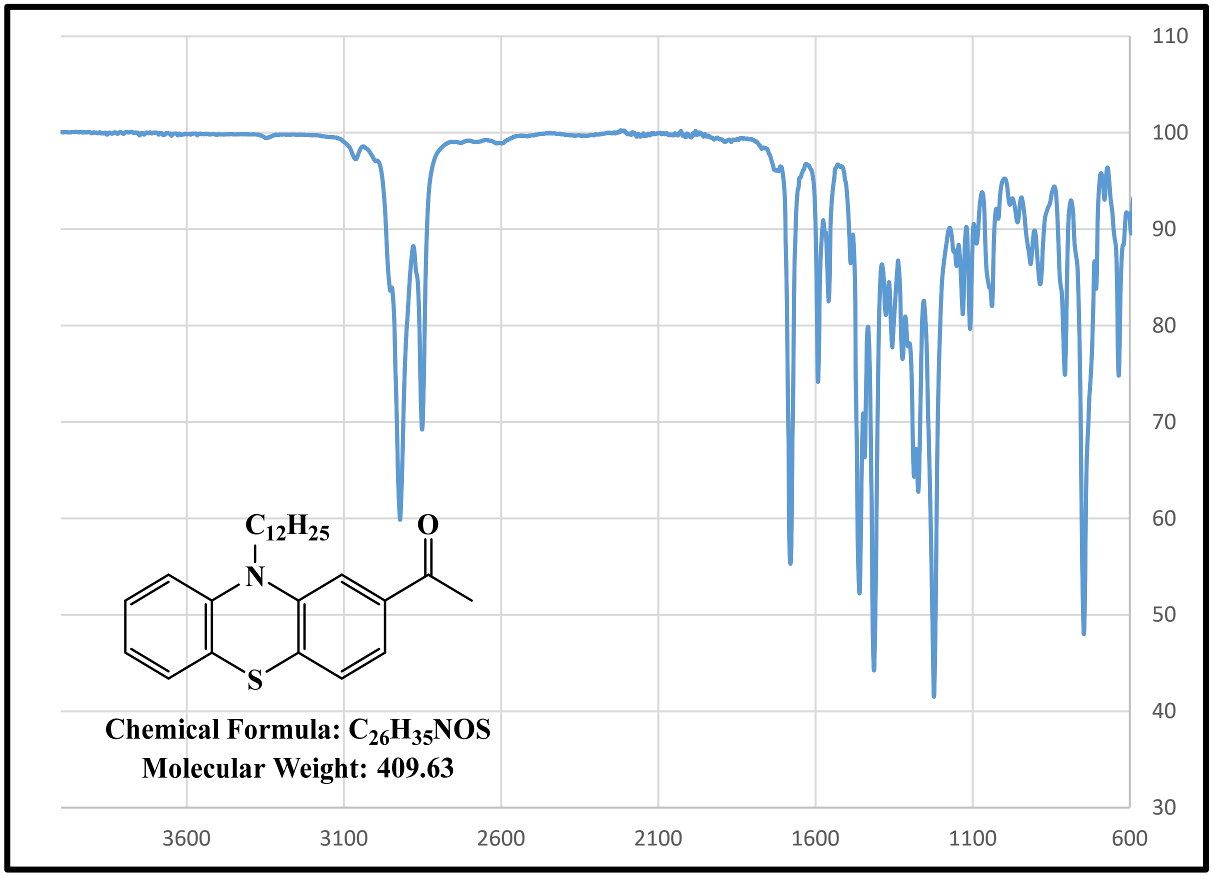

S5. 1-(10-dodecylphenothiazin-2-yl)**ethan-1-one**

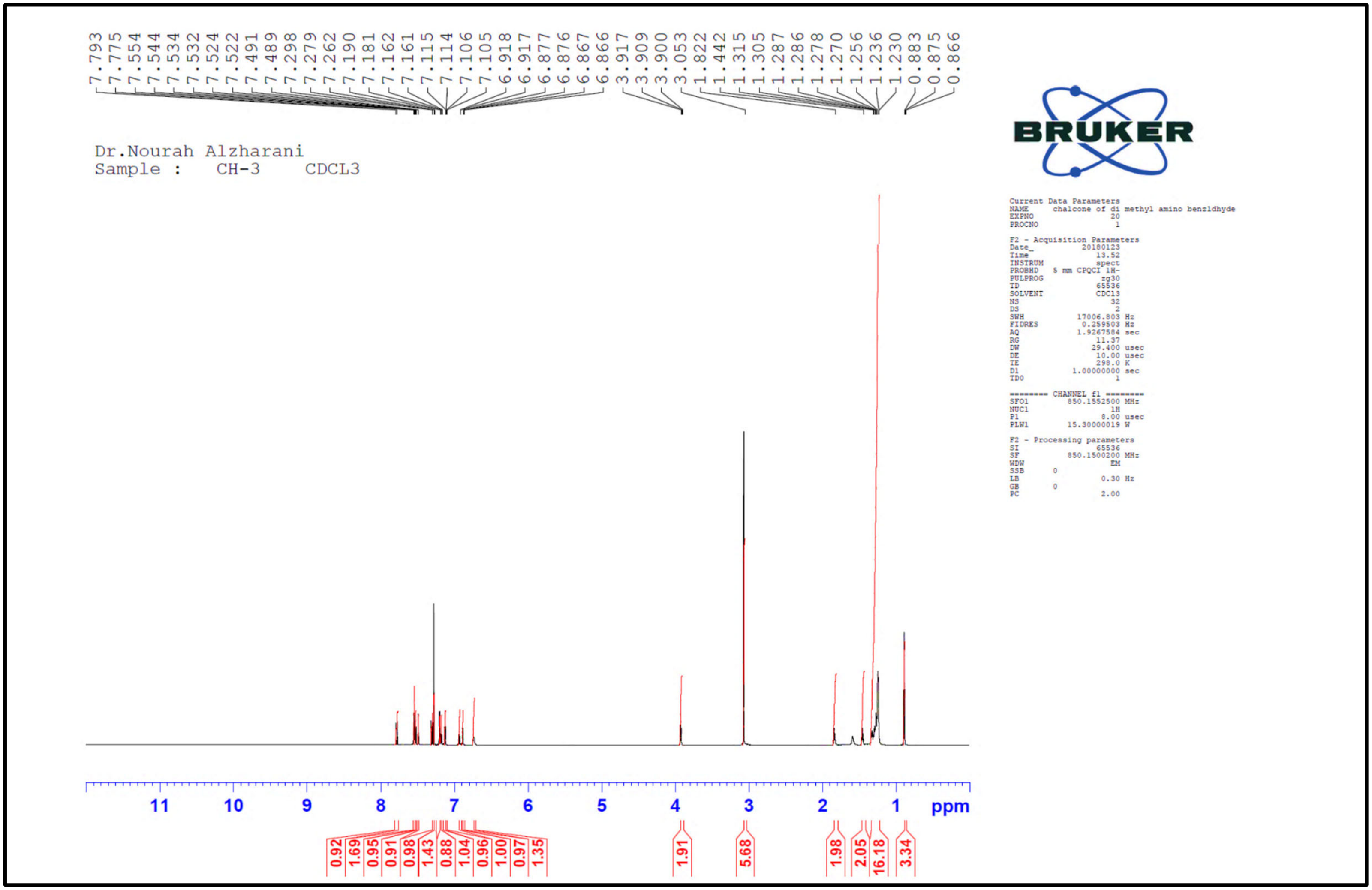

S6. ^{1}H NMR of (E)-3-(4-(dimethylamino)phenyl)-1-(*10-dodecylphenothiazin-2-yl*)prop-2-en-1-one (4a).

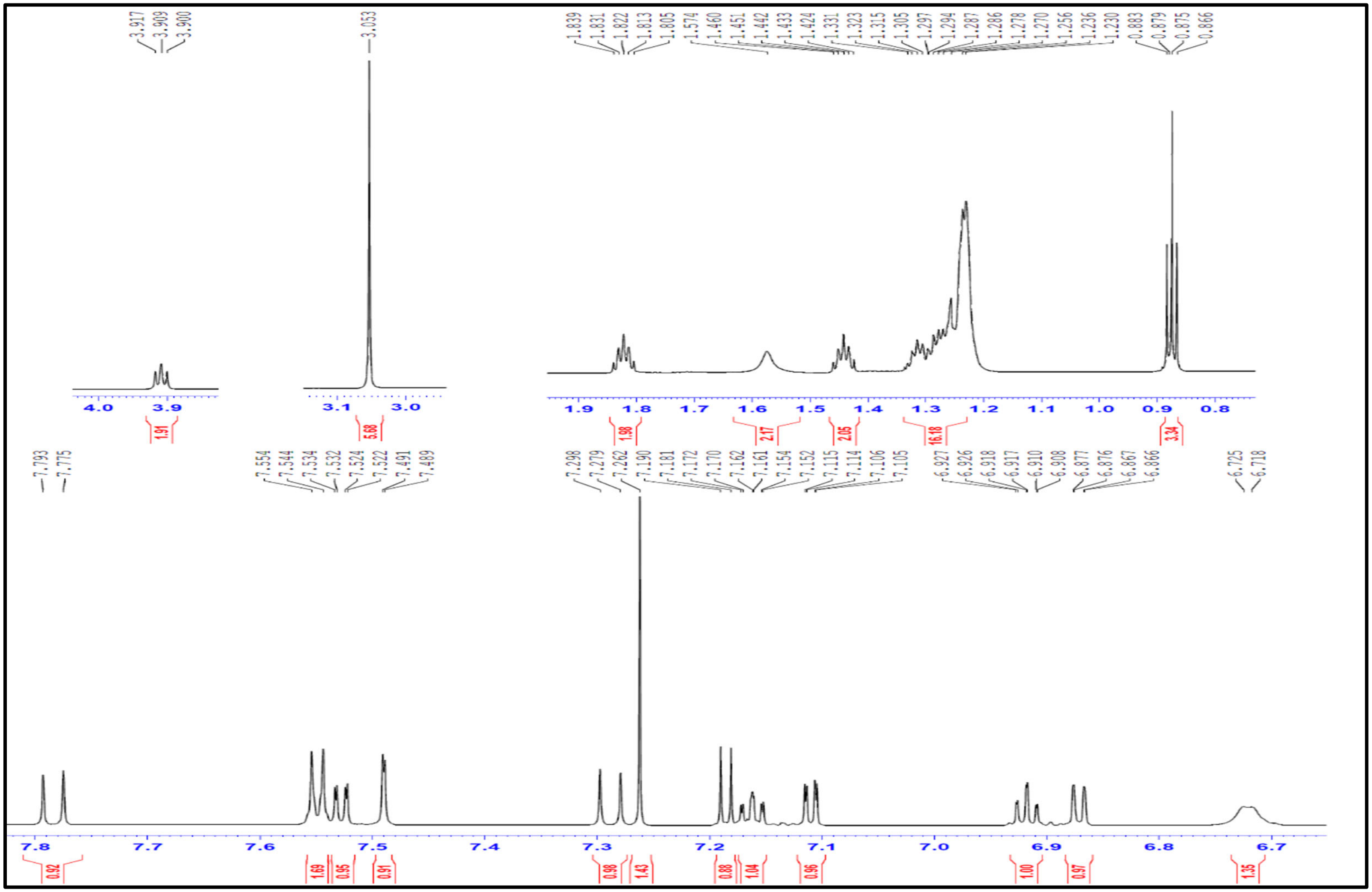

S7. ^{1}H NMR of (E)-3-(4-(dimethylamino)phenyl)-1-(*10-dodecylphenothiazin-2-yl*)prop-2-en-1-one (4a).

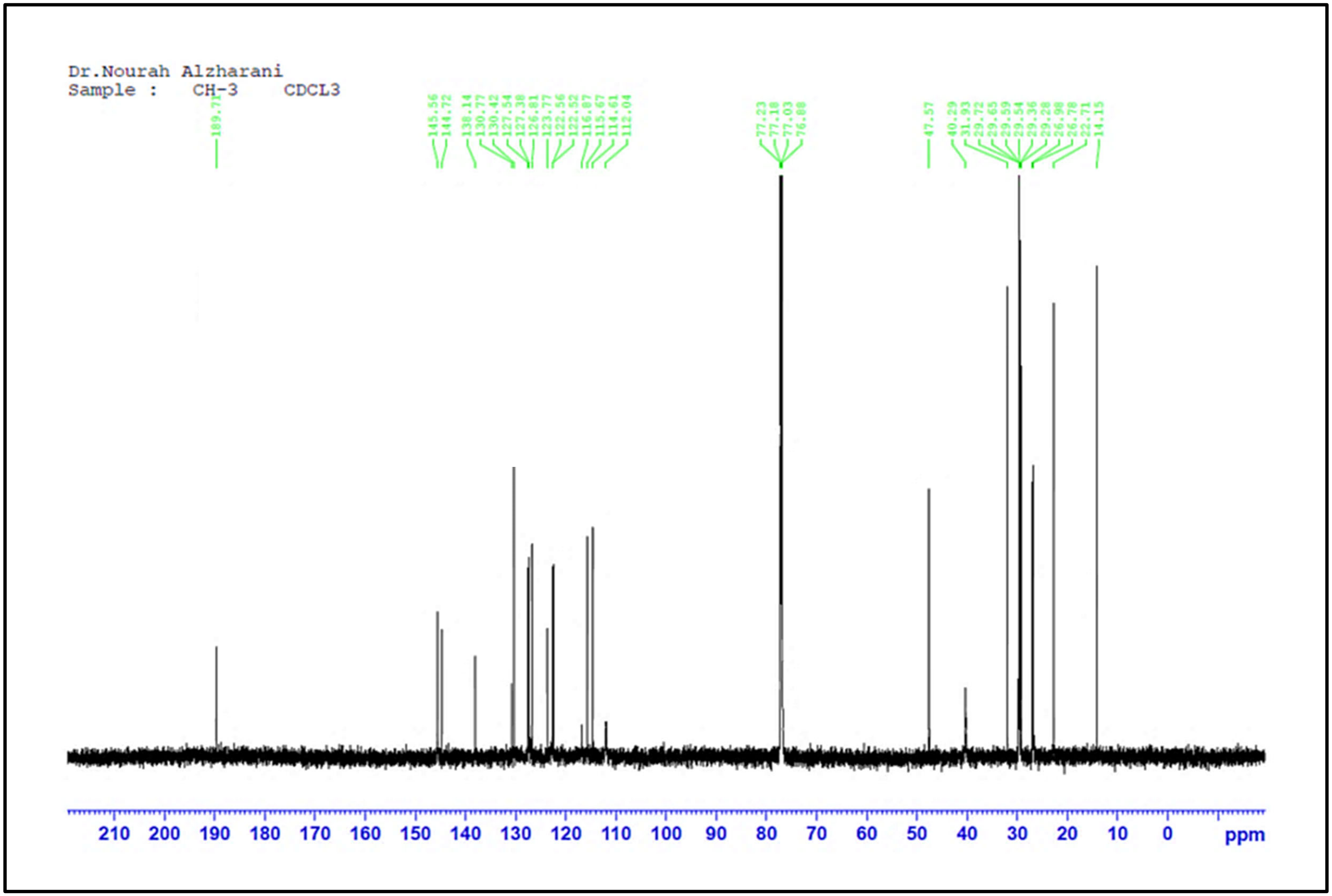

S8. ^{13}C NMR of (E)-3-(4-(dimethylamino)phenyl)-1-(*10-dodecylphenothiazin-2-yl*)prop-2-en-1-one (4a).

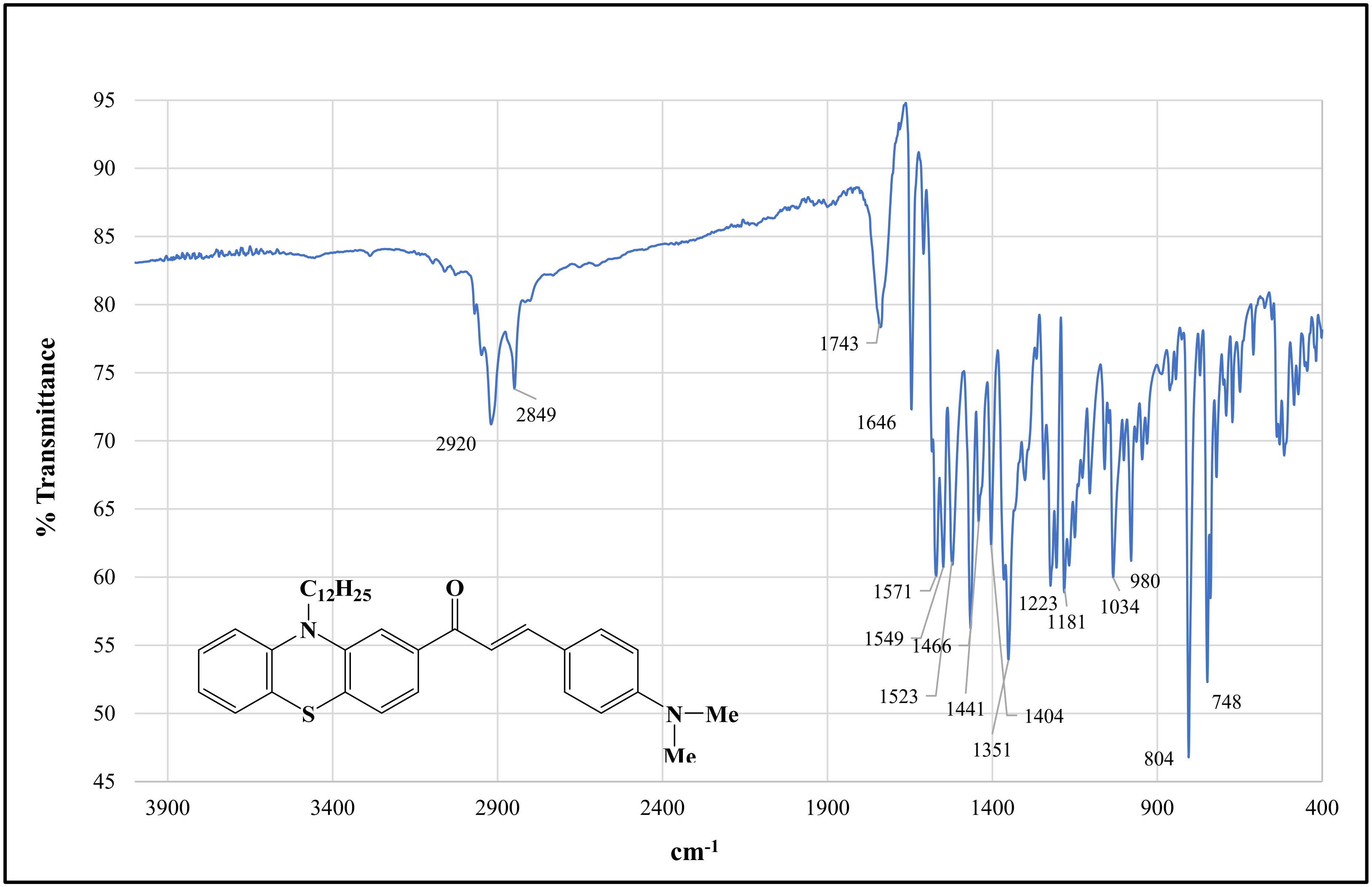

S9. IR of (E)-3-(4-(dimethylamino)phenyl)-1-(*10-dodecylphenothiazin-2-yl*)prop-2-en-1-one (4a).

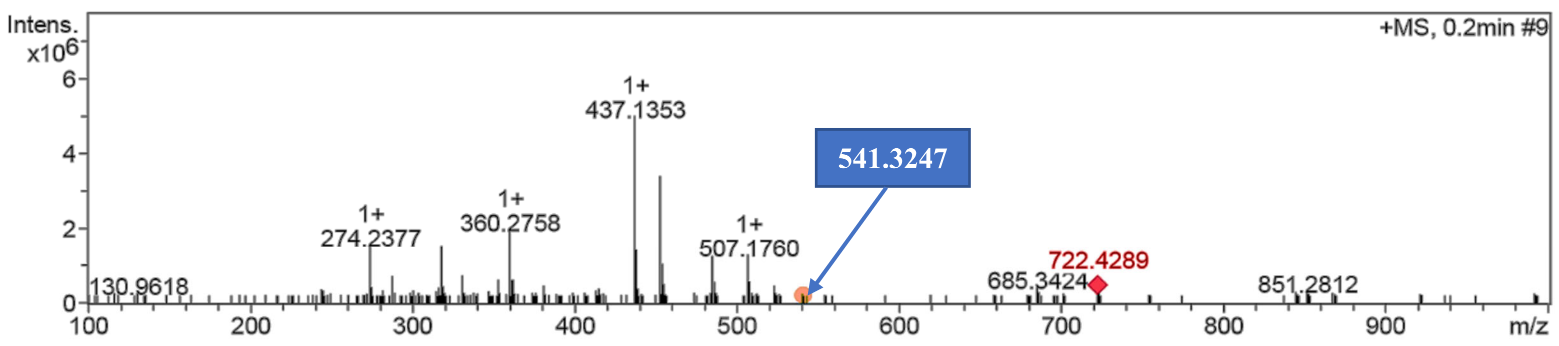

Meas. m/z	#	Ion Formula	m/z	err [ppm]	Mean err [ppm]	rdb	N-Rule	e⁻ Conf	mSigma	Std I	Std Mean m/z	Std I VarNorm	Std m/z Diff	Std Comb Dev
540.827861	1	C35H45N2OS	541.324711	917.8	979.1	14.5	ok	even	633.2	497.9	n.a.	n.a.	n.a.	n.a.

S10. MS of (E)-3-(4-(dimethylamino)phenyl)-1-(*10-dodecylphenothiazin-2-yl*)prop-2-en-1-one (4a).

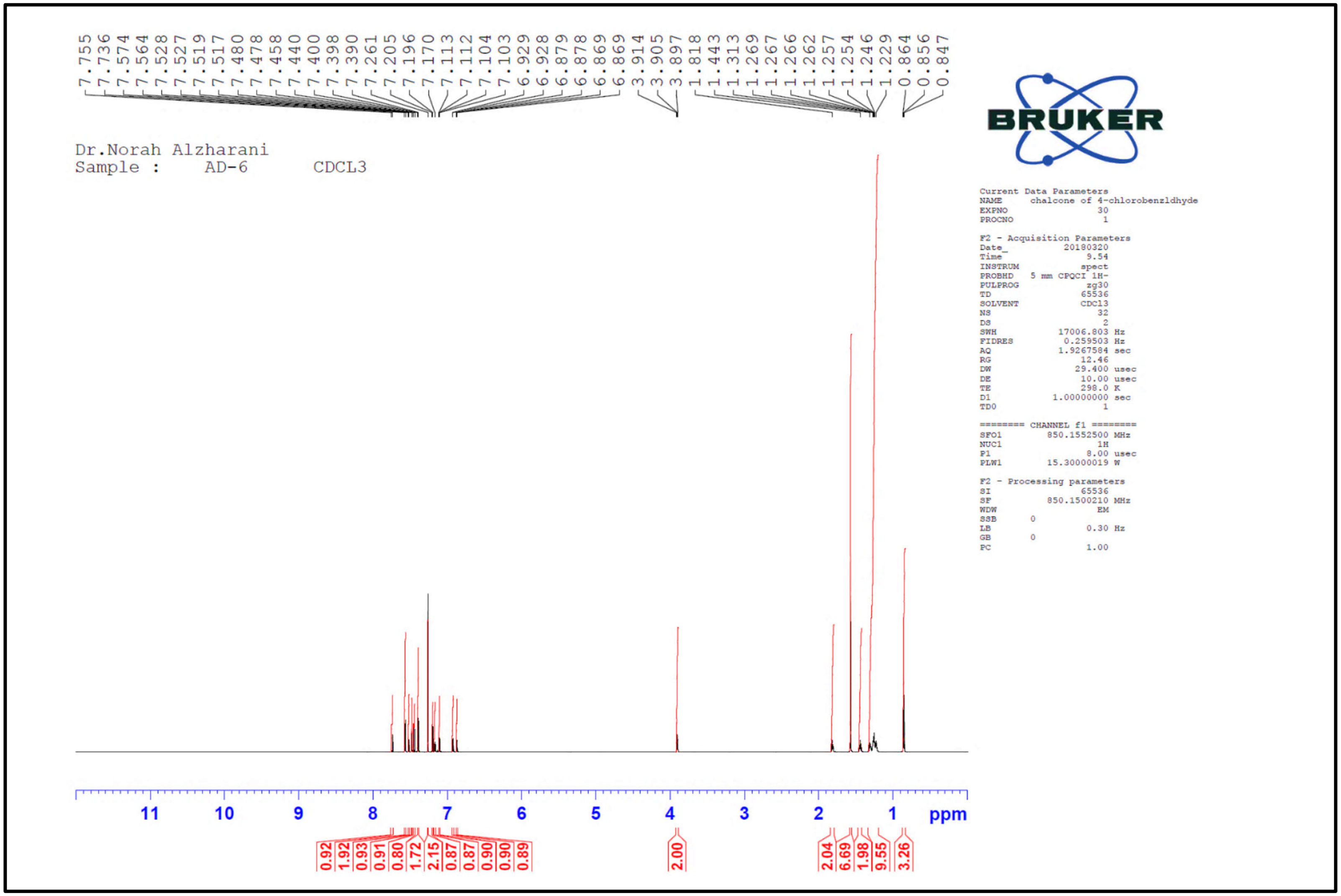

S11. ^{1}H NMR of (E)-3-(4-chlorophenyl)-1-(*10-dodecylphenothiazin-2-yl*)prop-2-en-1-one (4b).

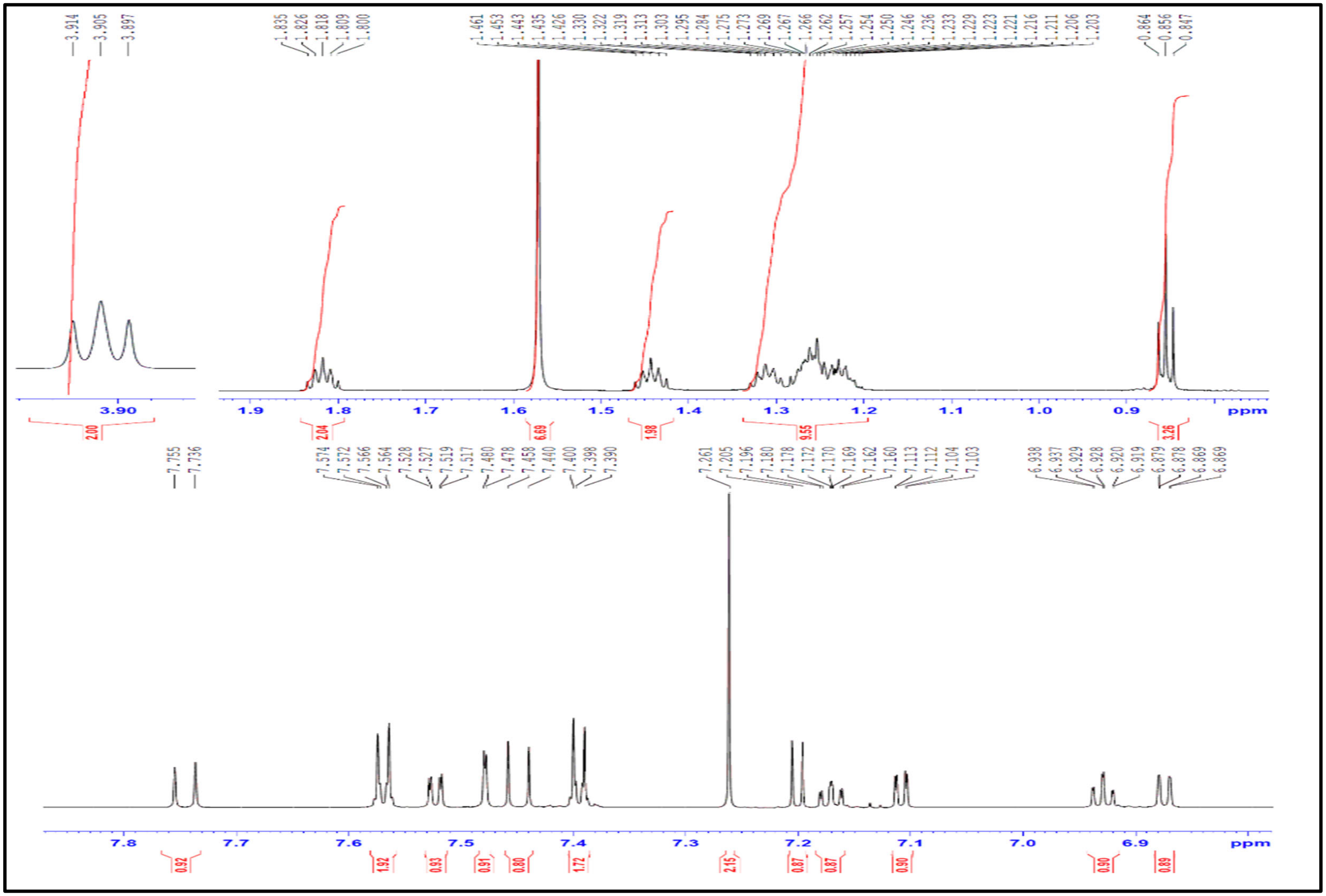

S12. ^{1}H NMR of (E)-3-(4-chlorophenyl)-1-(*10-dodecylphenothiazin-2-yl*)prop-2-en-1-one (4b).

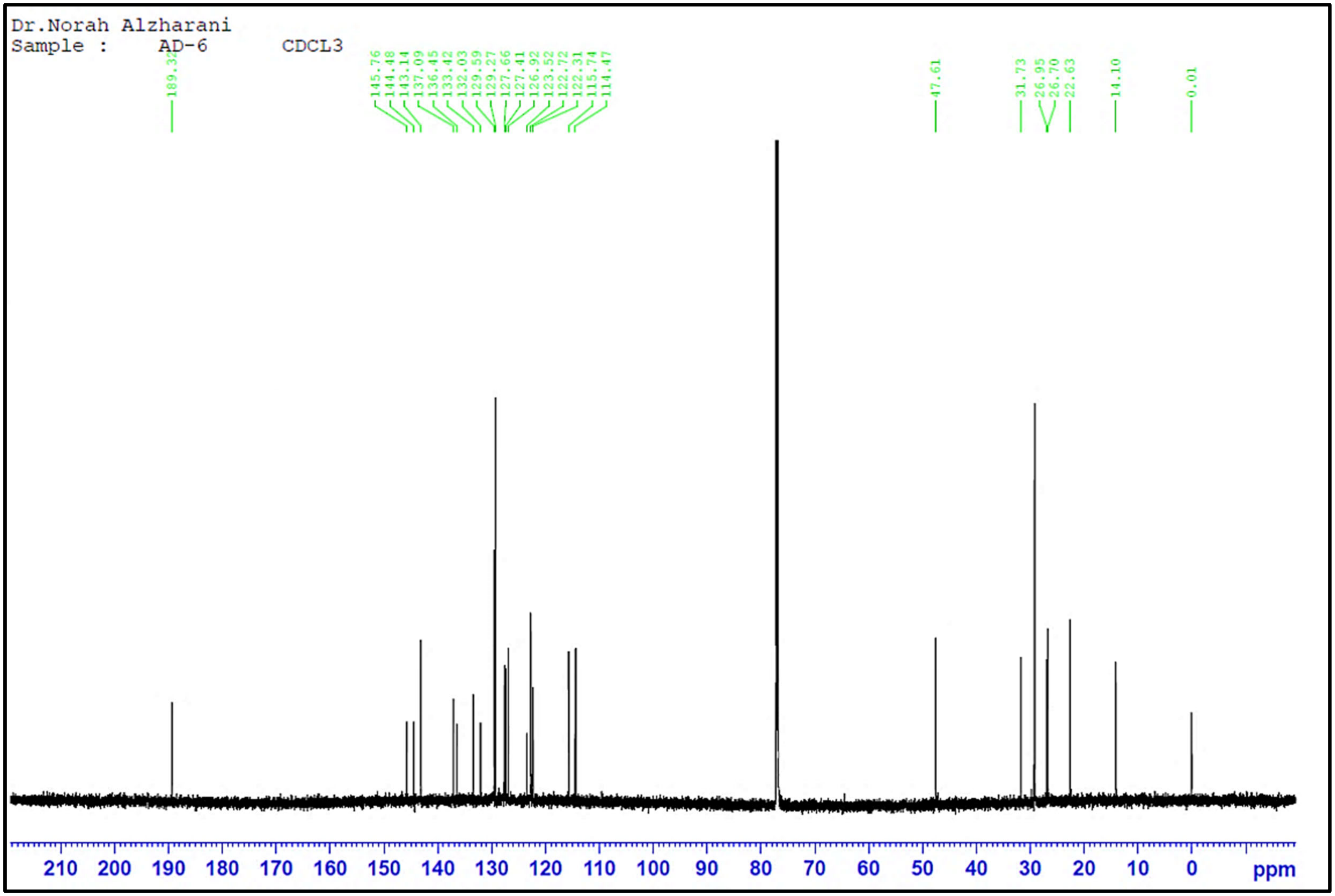

S13. ^{13}C NMR of (E)-3-(4-chlorophenyl)-1-(*10-dodecylphenothiazin-2-yl*)prop-2-en-1-one (4b).

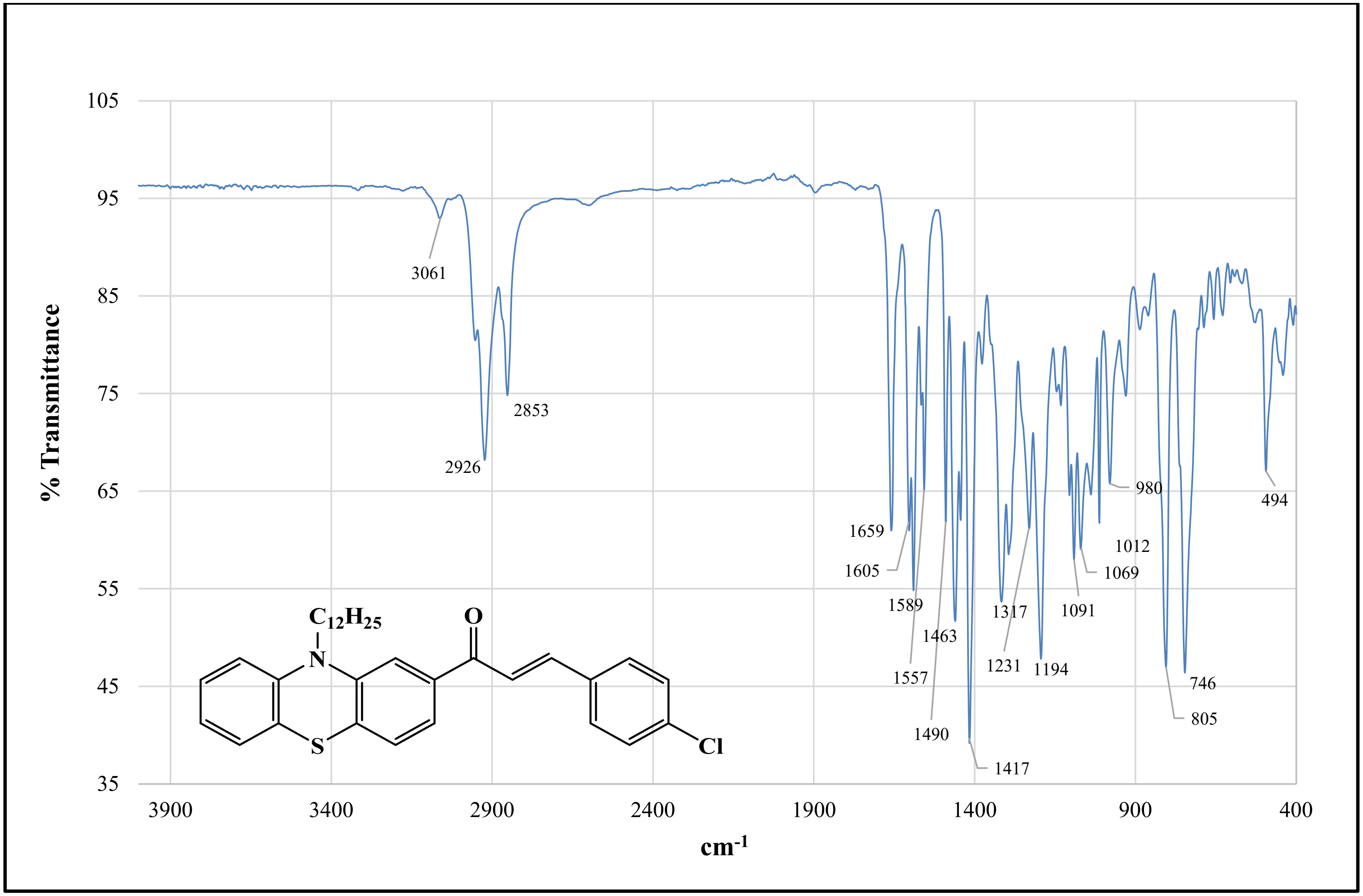

S14. IR of (E)-3-(4-chlorophenyl)-1-(*10-dodecylphenothiazin-2-yl*)prop-2-en-1-one (4b).

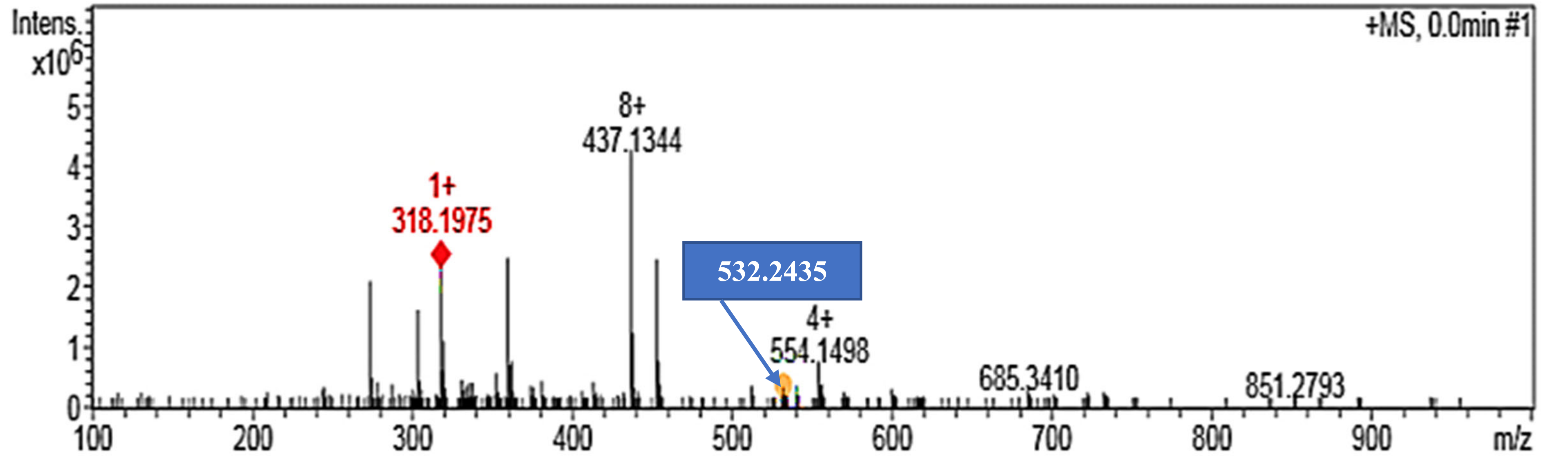

Meas. m/z	#	Ion Formula	m/z	err [ppm]	Mean err [ppm]	rdb	N-Rule	e⁻ Conf	mSigma	Std I	Std Mean m/z	Std I VarNorm	Std m/z Diff	Std Comb Dev
532.170407	1	C33H39ClNOS	532.243540	137.4	138.7	14.5	ok	even	23.8	26.9	n.a.	n.a.	n.a.	n.a.

S15. MS of (E)-3-(4-chlorophenyl)-1-(*10-dodecylphenothiazin-2-yl*)prop-2-en-1-one (4b).

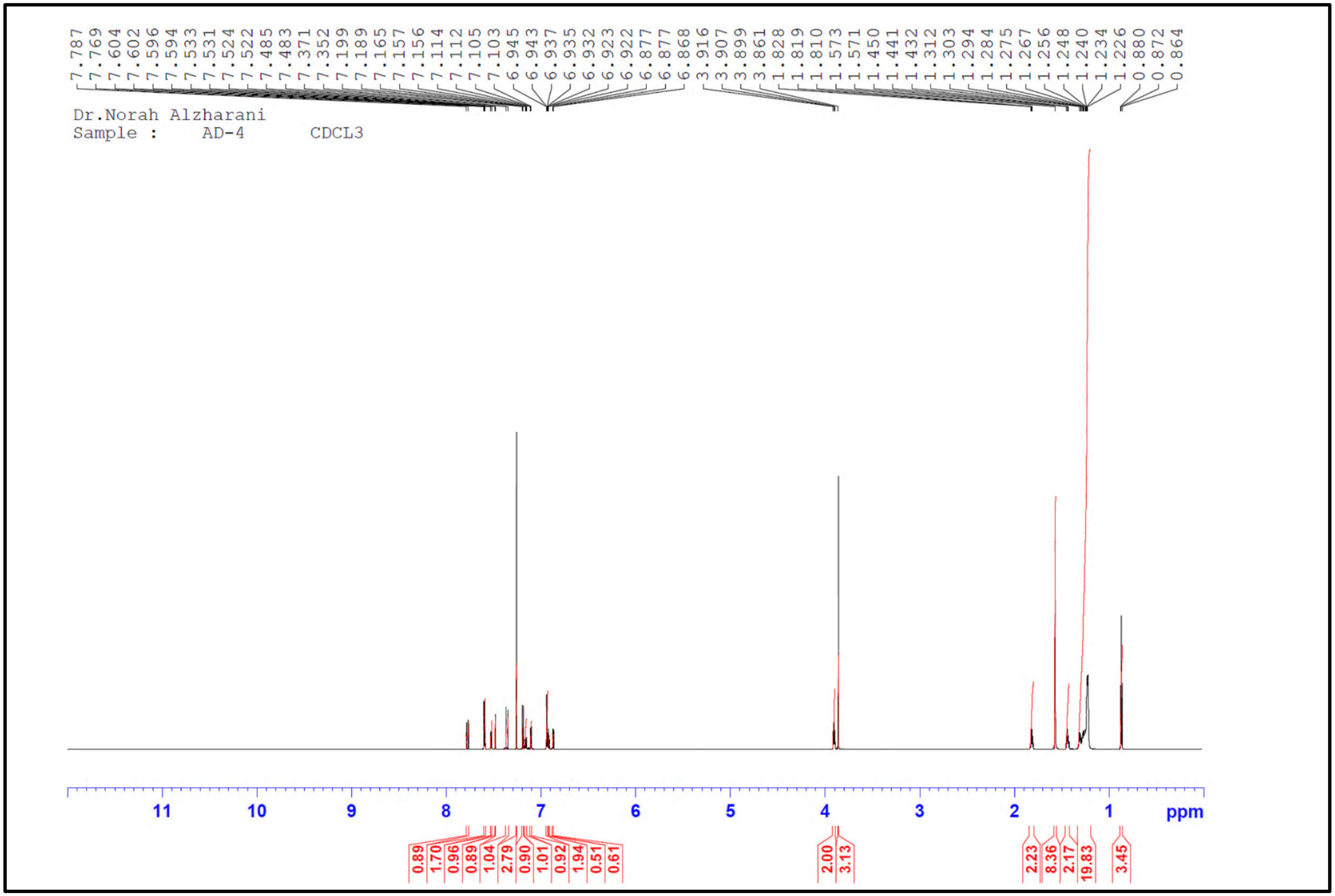

S16. ^{1}H NMR of (E)-1-(*10-dodecylphenothiazin-2-yl*)-3-(4-methoxyphenyl)prop-2-en-1-one (4c).

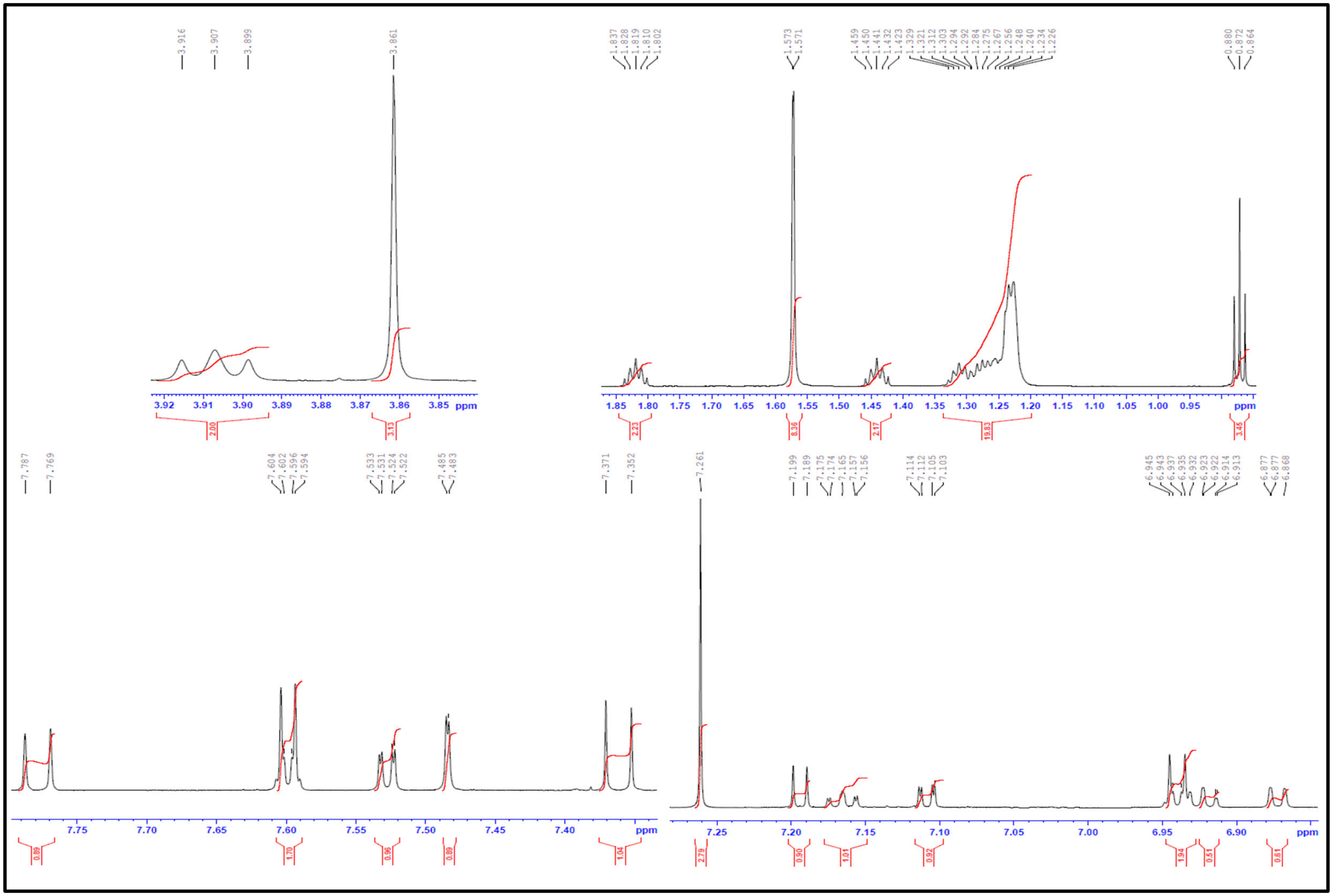

S17. ^{1}H NMR of (E)-1-(*10-dodecylphenothiazin-2-yl*)-3-(4-methoxyphenyl)prop-2-en-1-one (4c).

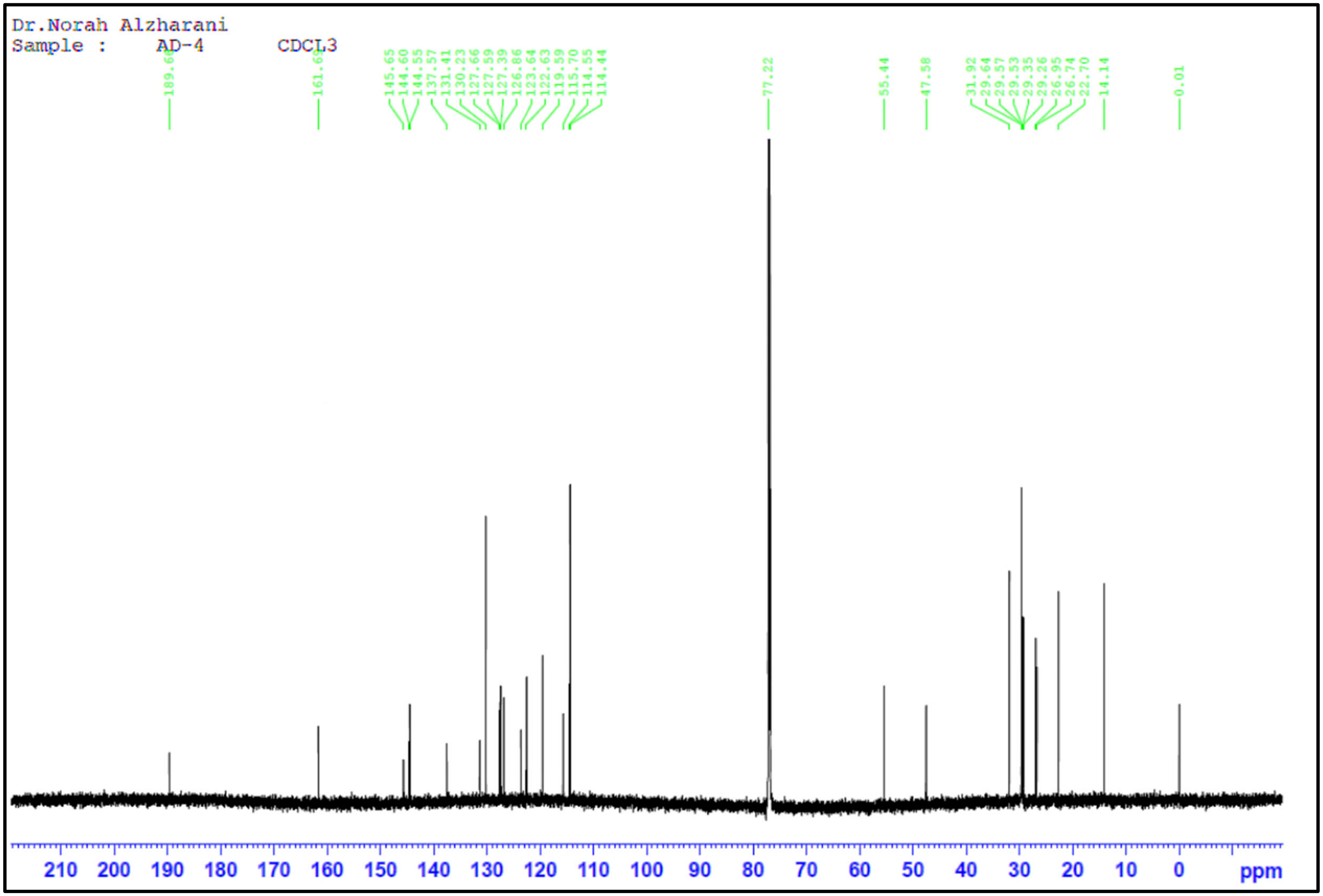

S18. ^{13}C NMR of (E)-1-(*10-dodecylphenothiazin-2-yl*)-3-(4-methoxyphenyl)prop-2-en-1-one (4c).

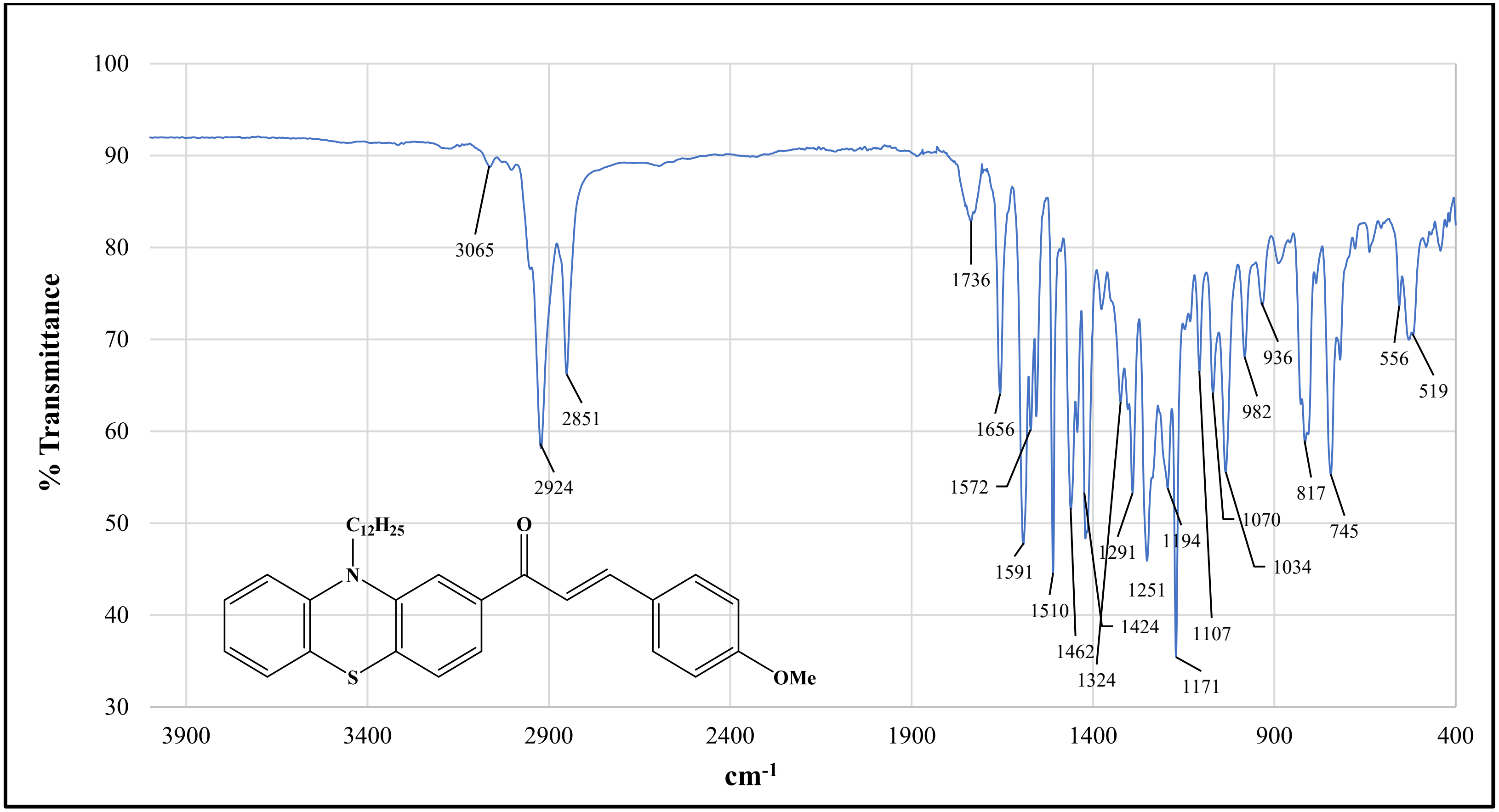

S19. IR of (E)-1-(*10-dodecylphenothiazin-2-yl*)-3-(4-methoxyphenyl)prop-2-en-1-one (4c).

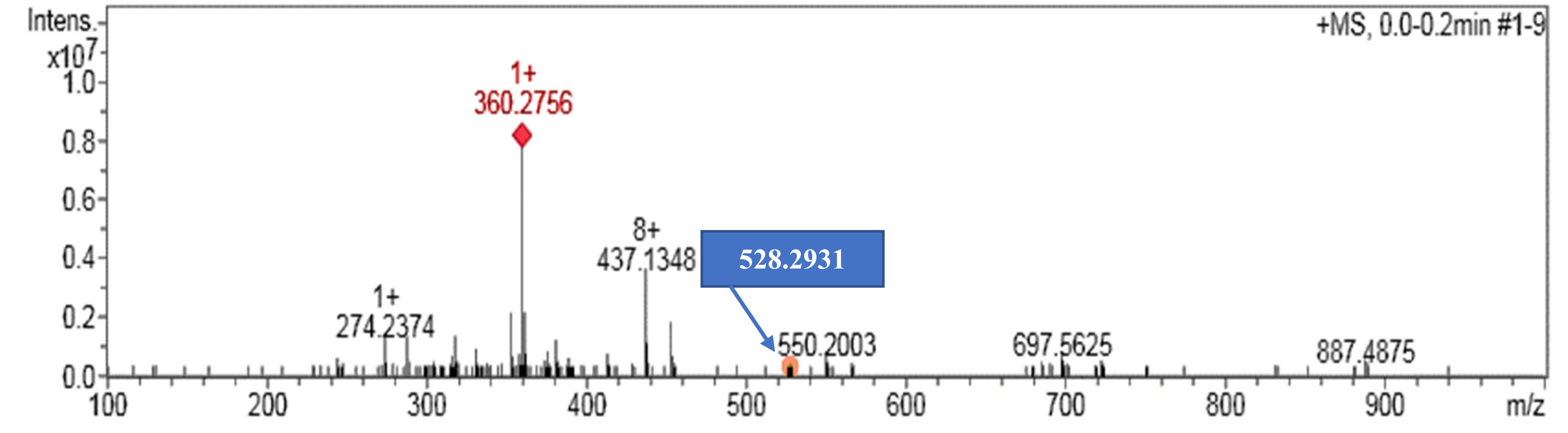

Meas. m/z	#	Ion Formula	m/z	err [ppm]	Mean err [ppm]	rdb	N-Rule	e⁻ Conf	mSigma	Std I	Std Mean m/z	Std I VarNorm	Std m/z Diff	Std Comb Dev
527.785139	1	C34H42NO2S	528.293077	961.5	961.2	14.5	ok	even	97.3	137.5	n.a.	n.a.	n.a.	n.a.

S20. MS of (E)-1-(*10-dodecylphenothiazin-2-yl*)-3-(4-methoxyphenyl)prop-2-en-1-one (4c).

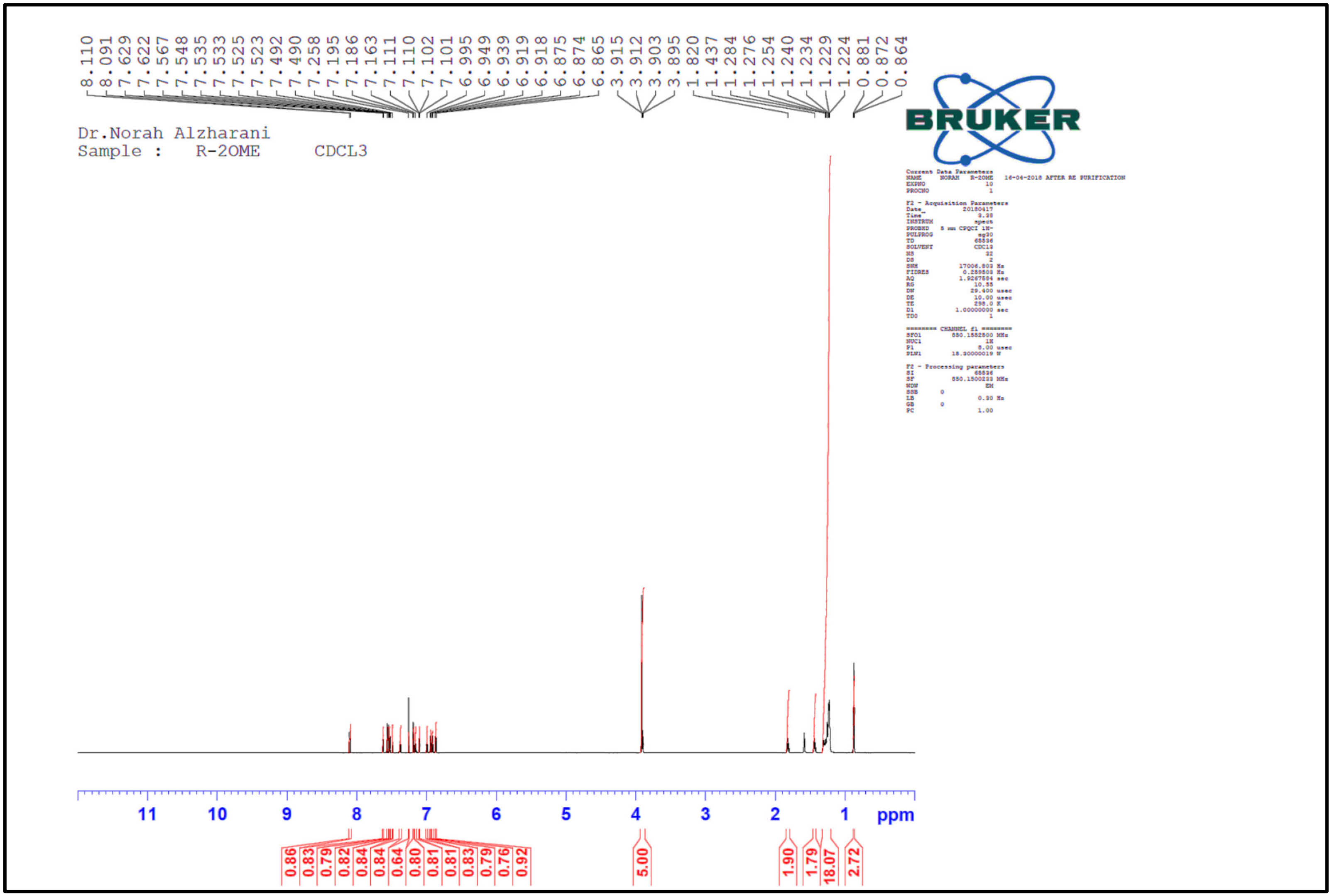

S21. [1]**H NMR of 1-(*10-dodecylphenothiazin-2-yl*)-3-(2-methoxy-phenyl)-propenone (4d).**

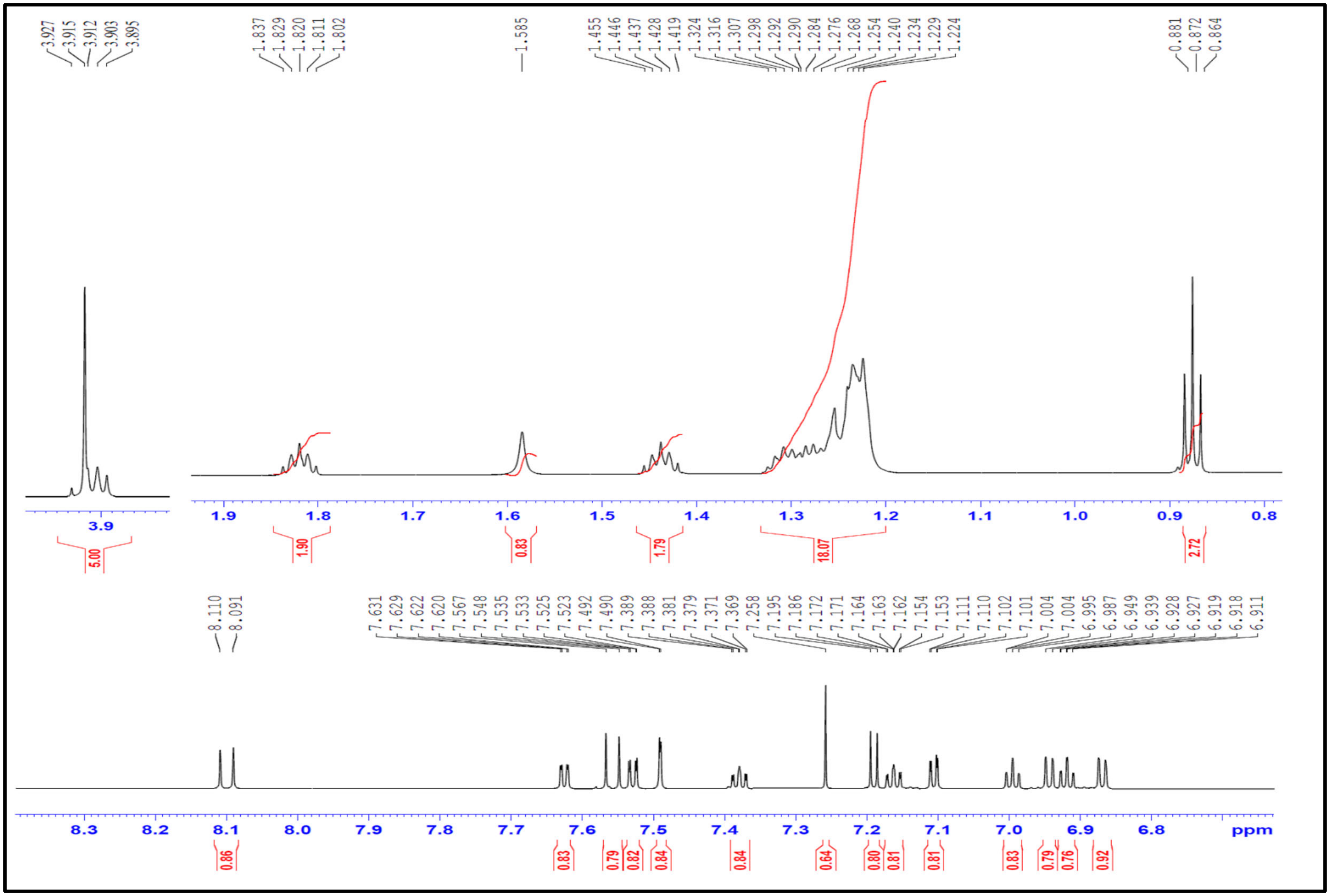

S22. ^{1}H NMR of 1-(*10-dodecylphenothiazin-2-yl*)-3-(2-methoxy-phenyl)-propenone (4d).

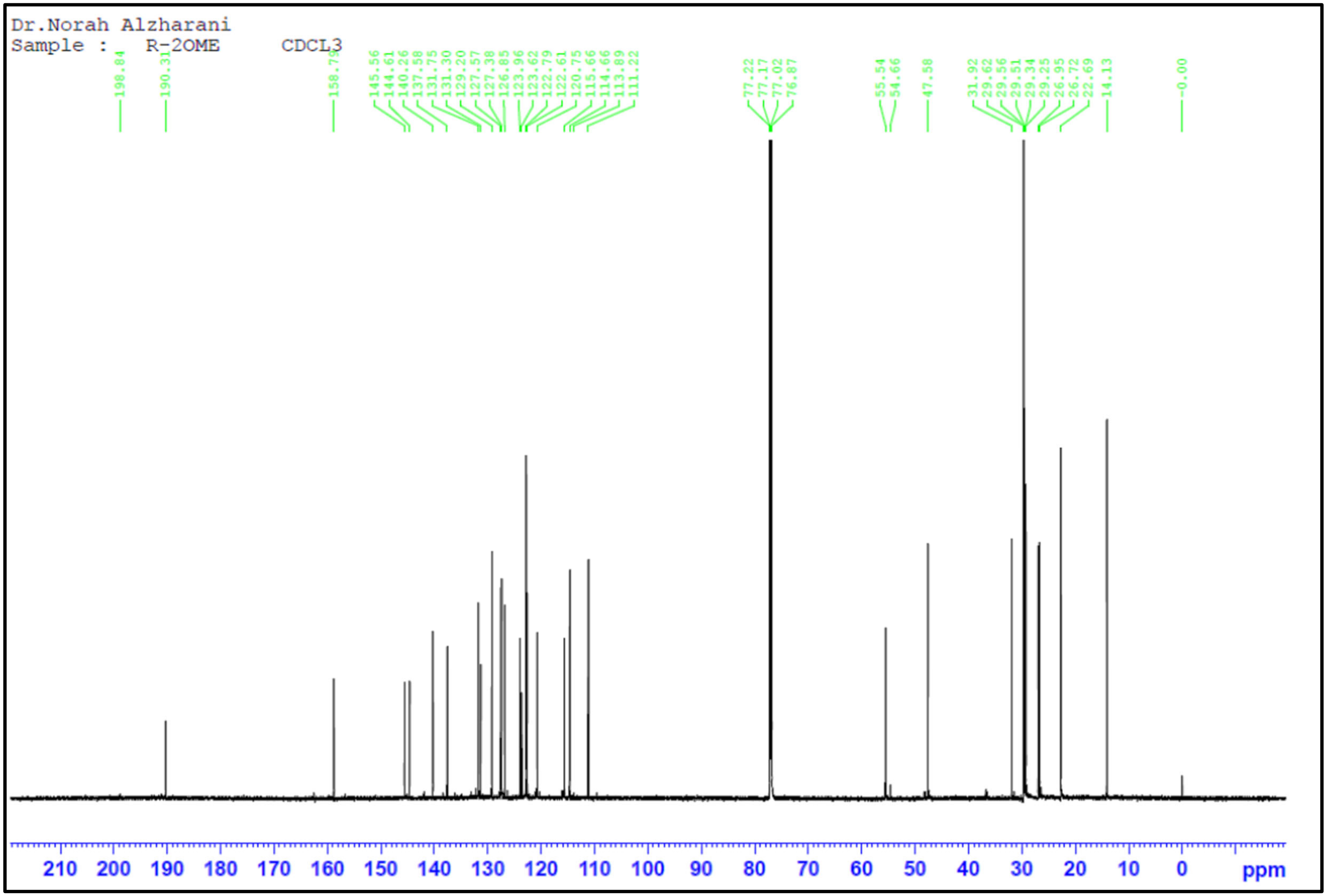

S23. ^{13}C NMR of 1-(*10-dodecylphenothiazin-2-yl*)-3-(2-methoxy-phenyl)-propenone (4d).

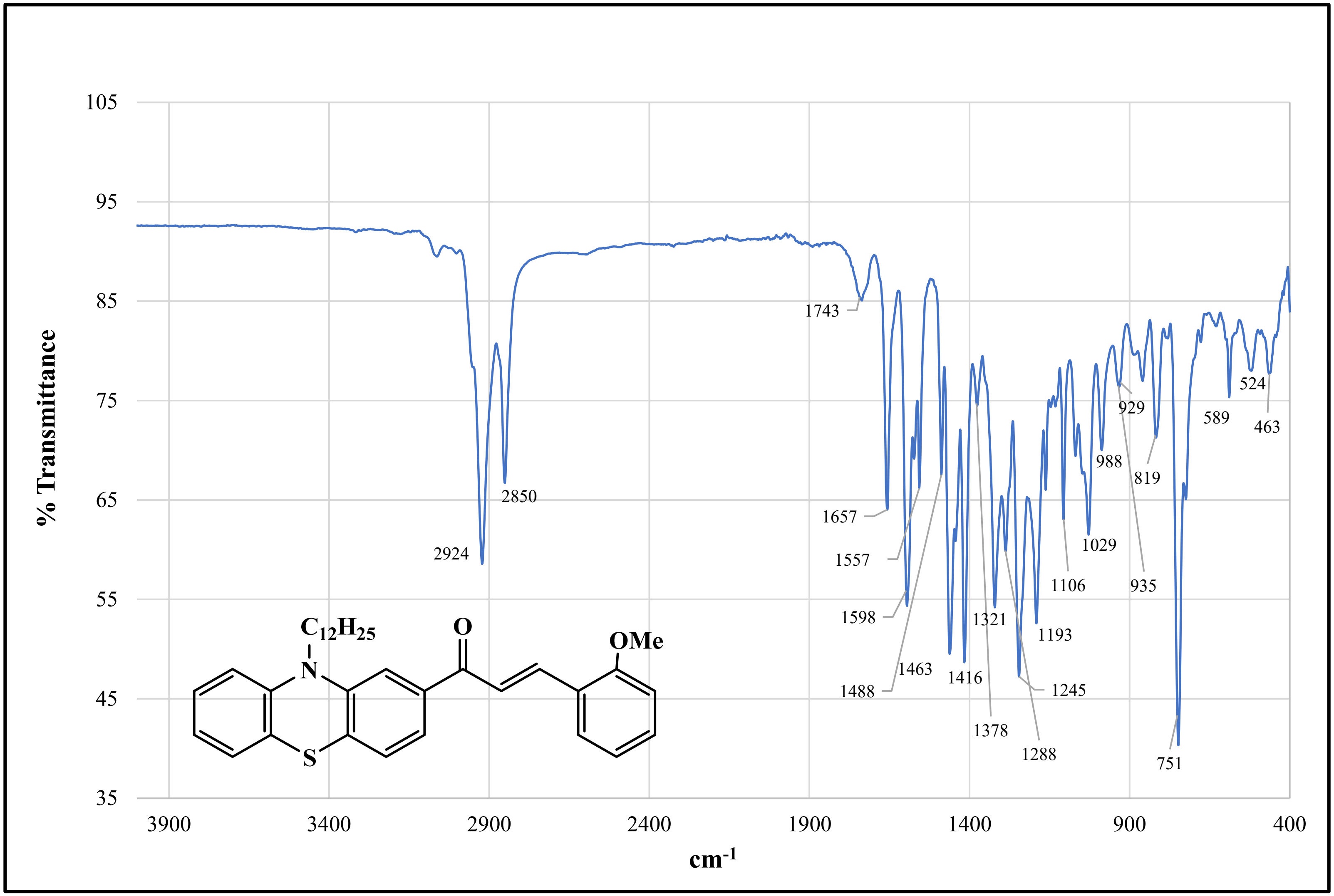

S24. IR of 1-(*10-dodecylphenothiazin-2-yl*)-3-(2-methoxy-phenyl)-propenone (4d).

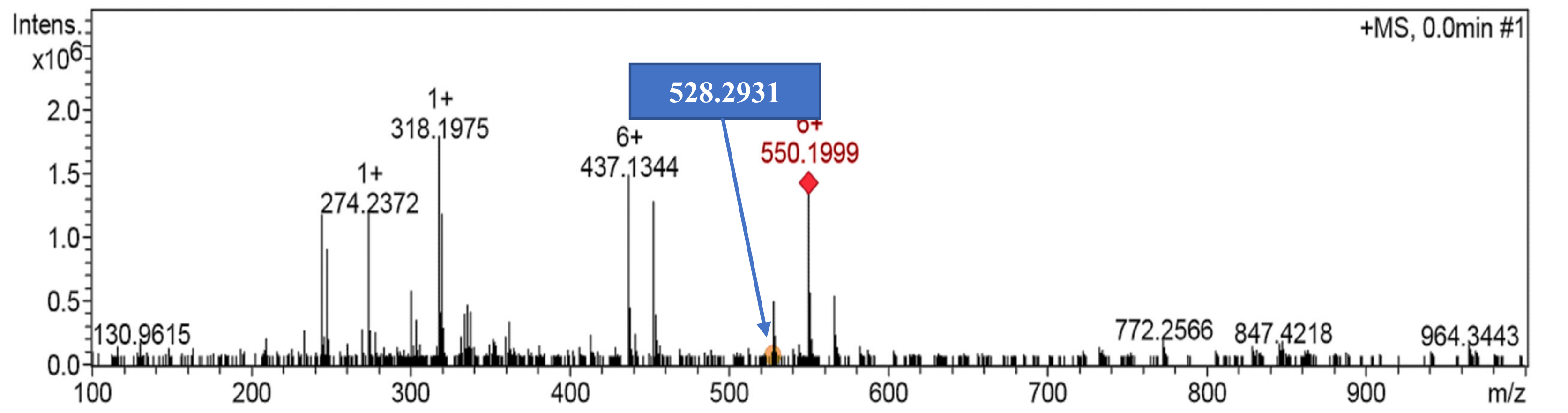

Meas. m/z	#	Ion Formula	m/z	err [ppm]	Mean err [ppm]	rdb	N-Rule	e⁻ Conf	mSigma	Std I	Std Mean m/z	Std I VarNorm	Std m/z Diff	Std Comb Dev
527.761676	1	C34H42NO2S	528.293077	1005.9	1031.0	14.5	ok	even	501.0	454.3	n.a.	n.a.	n.a.	n.a.

S25. MS of 1-(10-dodecylphenothiazin-2-yl**)-3-(2-methoxy-phenyl)-propenone (4d).**

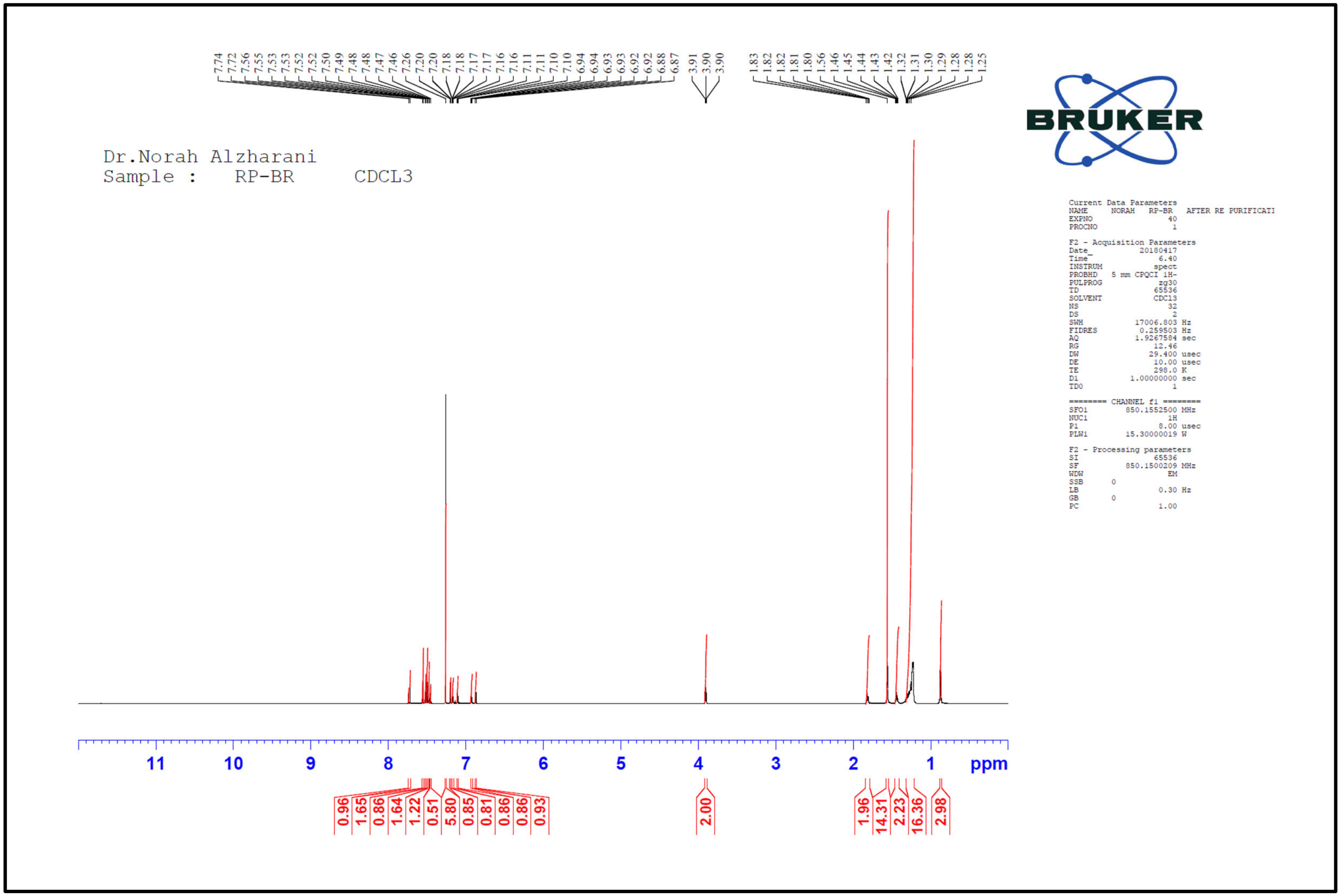

S26. ^{1}H NMR of 3-(4-Bromo-phenyl)-1-(*10-dodecylphenothiazin-2-yl*)-propenone (4e).

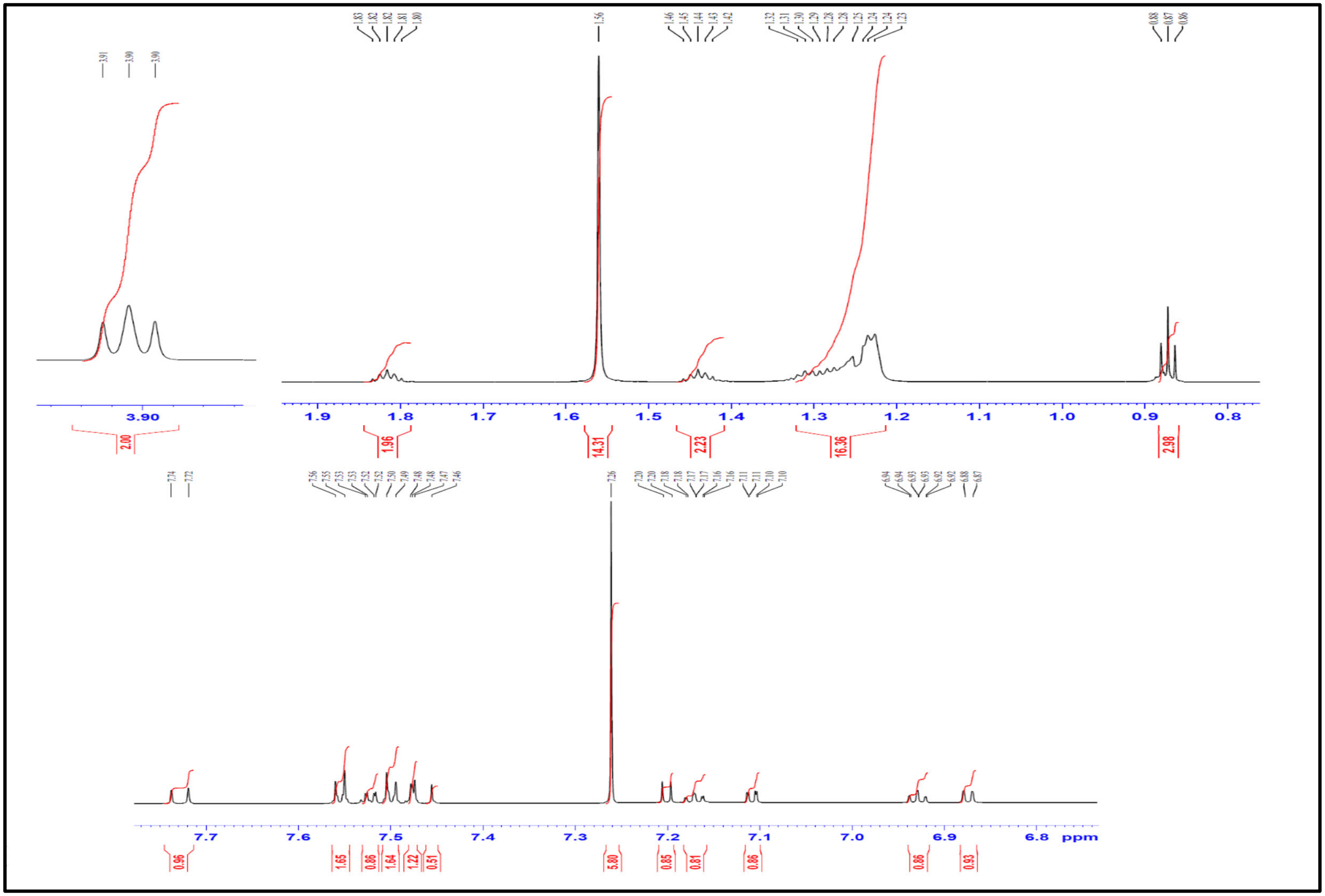

S27. ^{1}H NMR of 3-(4-Bromo-phenyl)-1-(*10-dodecylphenothiazin-2-yl*)-propenone (4e).

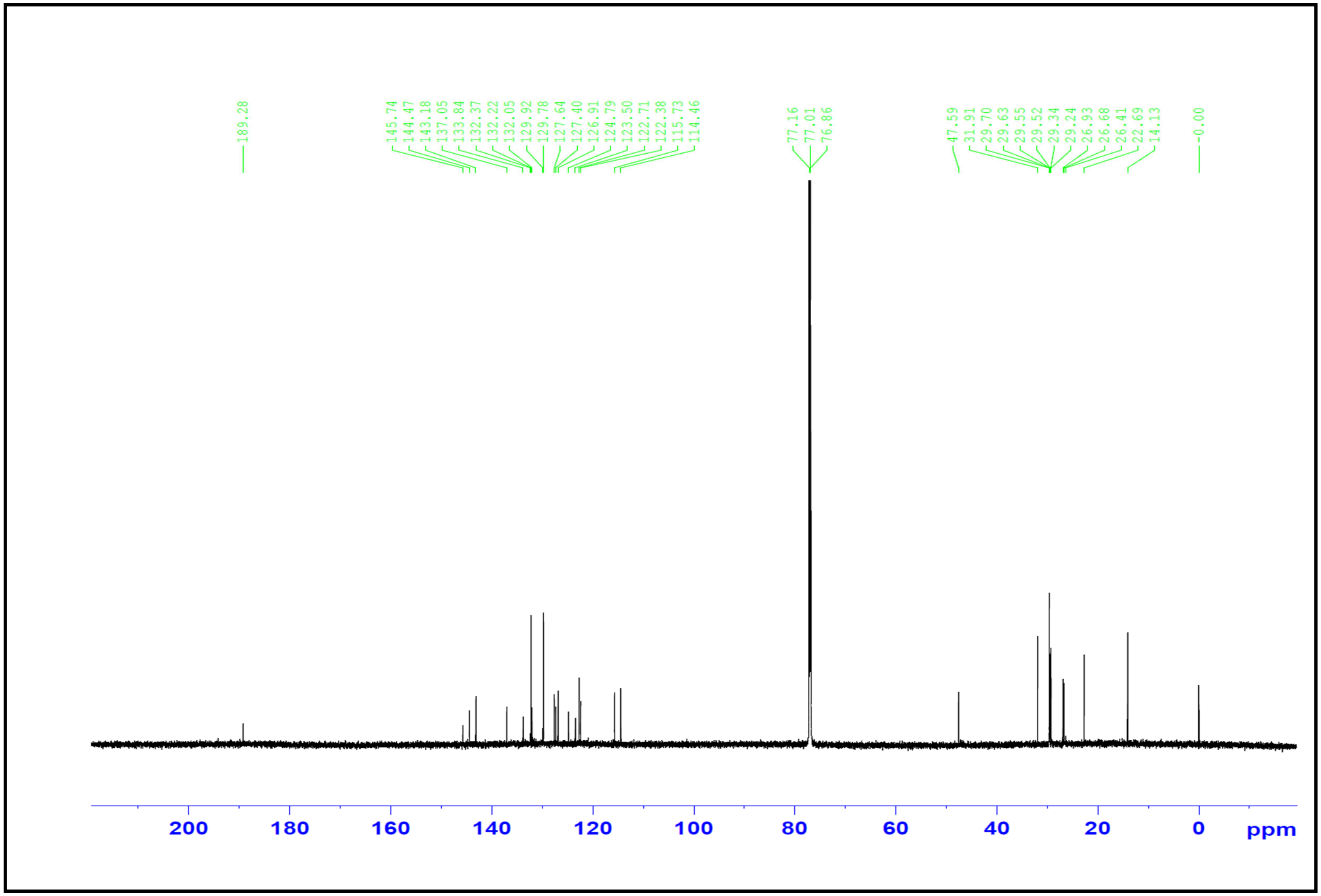

S28. ^{13}C NMR of 3-(4-Bromo-phenyl)-1-(*10-dodecylphenothiazin-2-yl*)-propenone (4e).

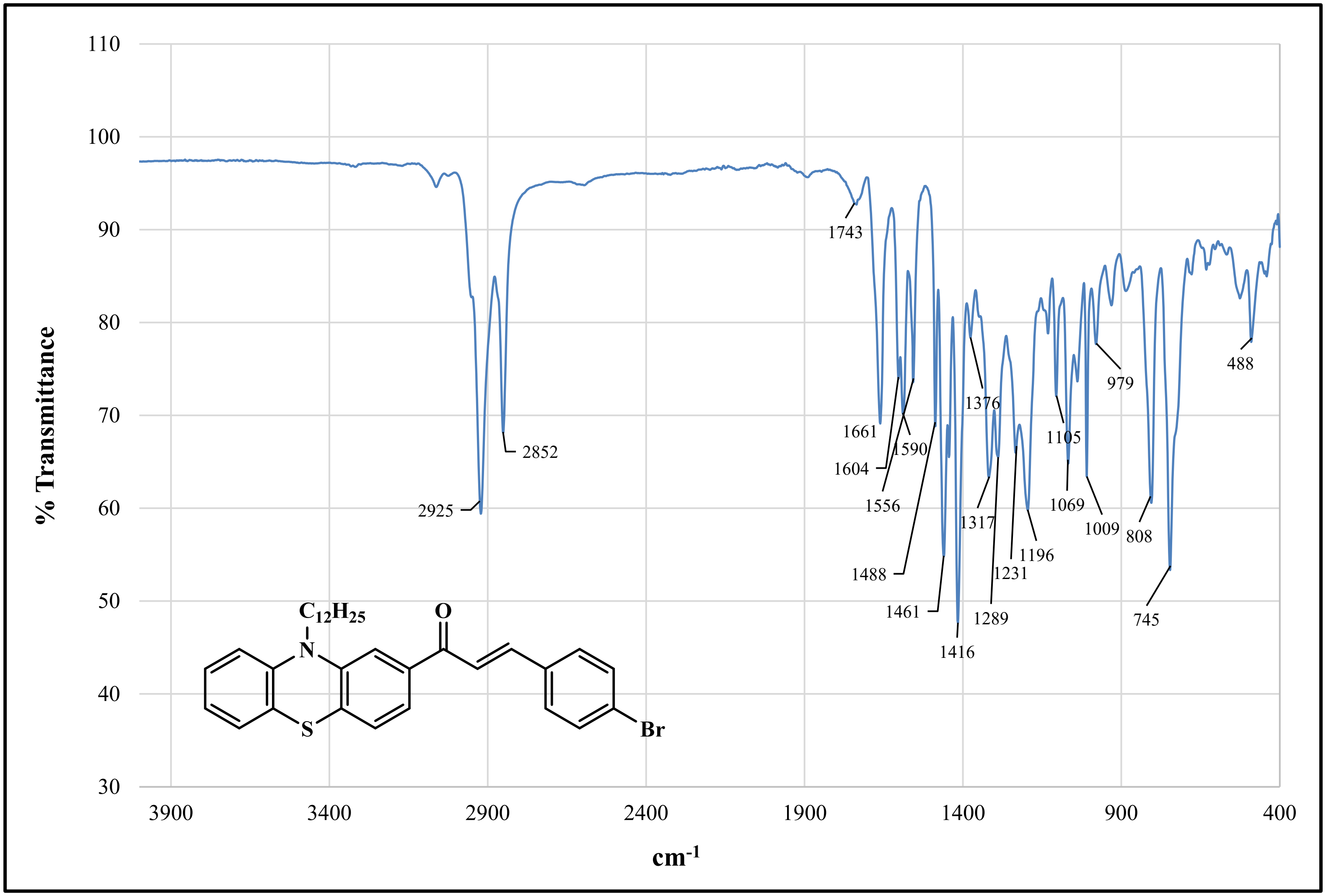

S29. IR of 3-(4-Bromo-phenyl)-1-(*10-dodecylphenothiazin-2-yl*)-propenone (4e).

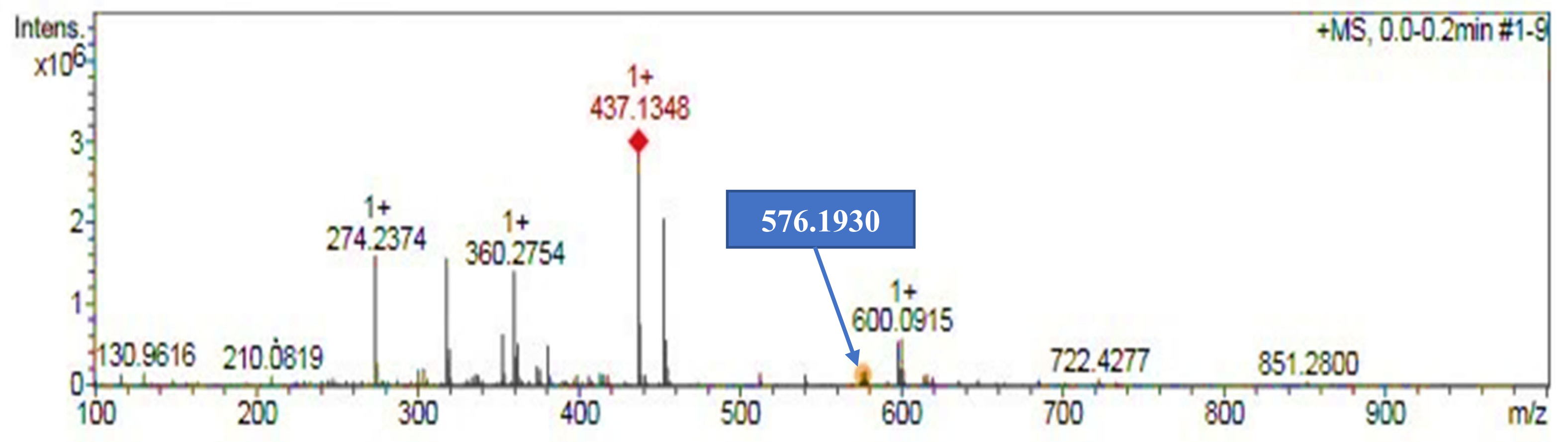

Meas. m/z	#	Ion Formula	m/z	err [ppm]	Mean err [ppm]	rdb	N-Rule	e⁻ Conf	mSigma	Std I	Std Mean m/z	Std I VarNorm	Std m/z Diff	Std Comb Dev
576.626210	1	C33H39BrNOS	576.193024	-751.8	-733.3	14.5	ok	even	403.8	329.8	n.a.	n.a.	n.a.	n.a.

S30. MS of 3-(4-Bromo-phenyl)-1-(*10-dodecylphenothiazin-2-yl*)-propenone (4e).

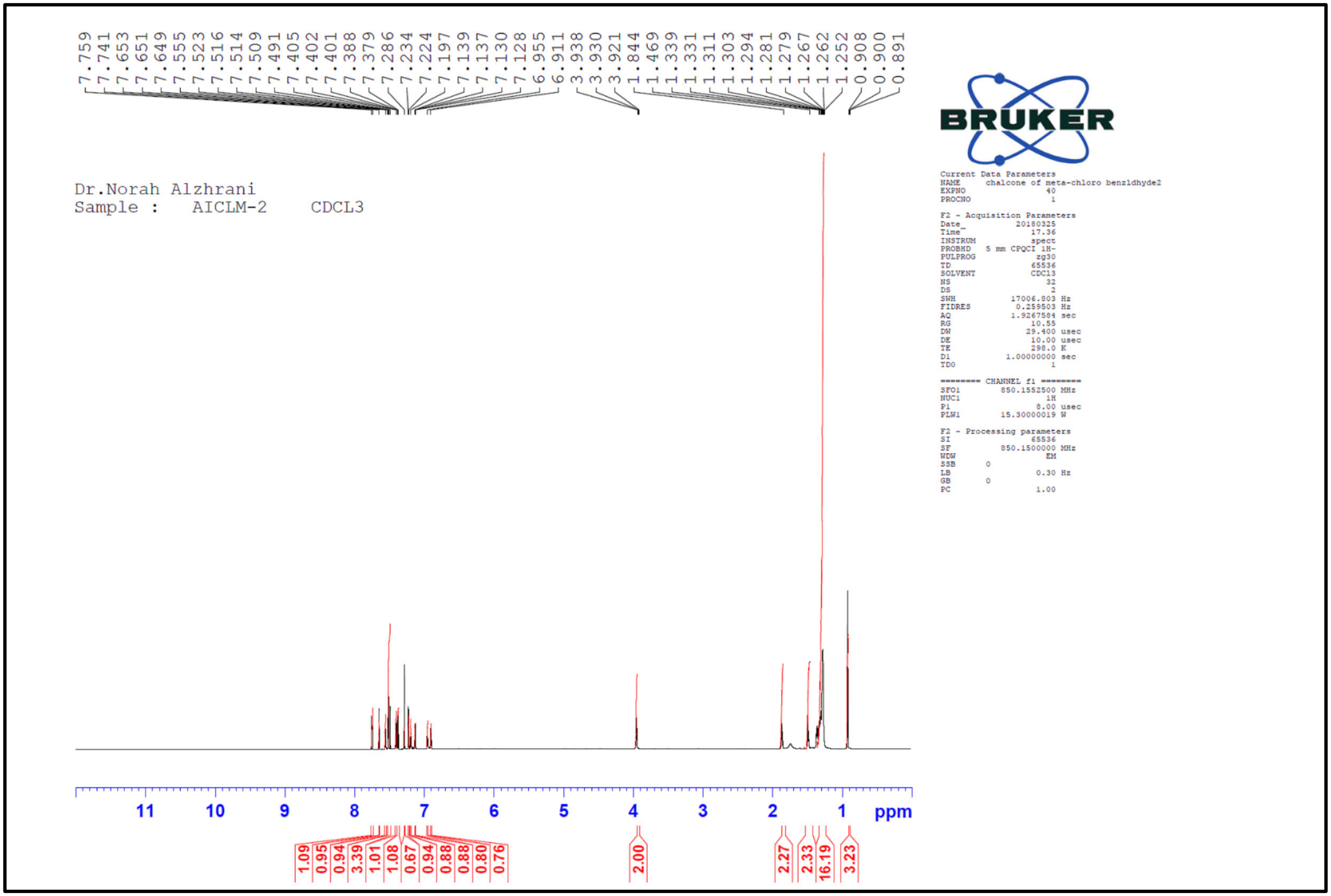

S31. ^{1}H NMR of 3-(3-Chloro-phenyl)-1-(*10-dodecylphenothiazin-2-yl*)-propenone (4f).

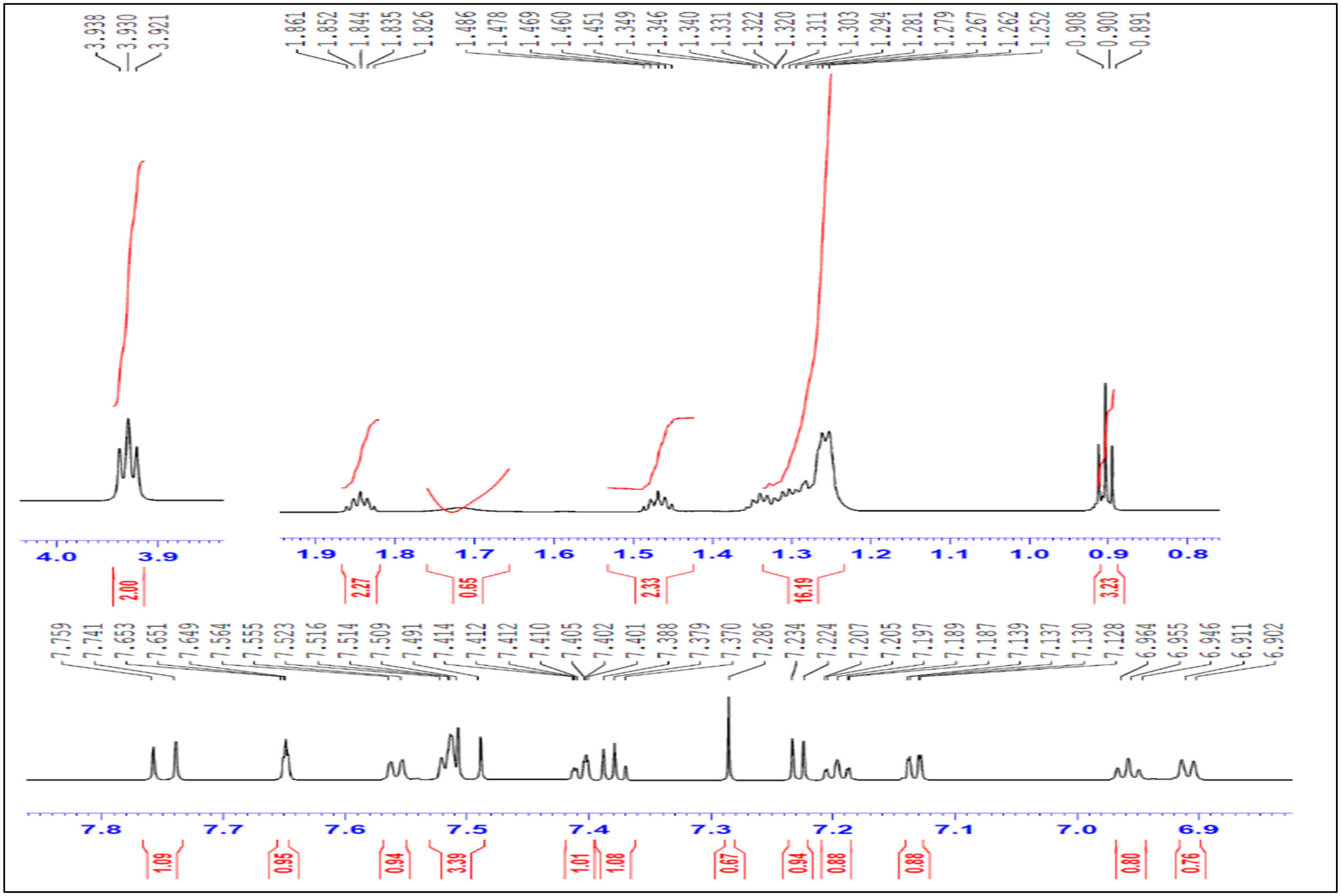

S32. ^{1}H NMR of 3-(3-Chloro-phenyl)-1-(*10-dodecylphenothiazin-2-yl*)-propenone (4f).

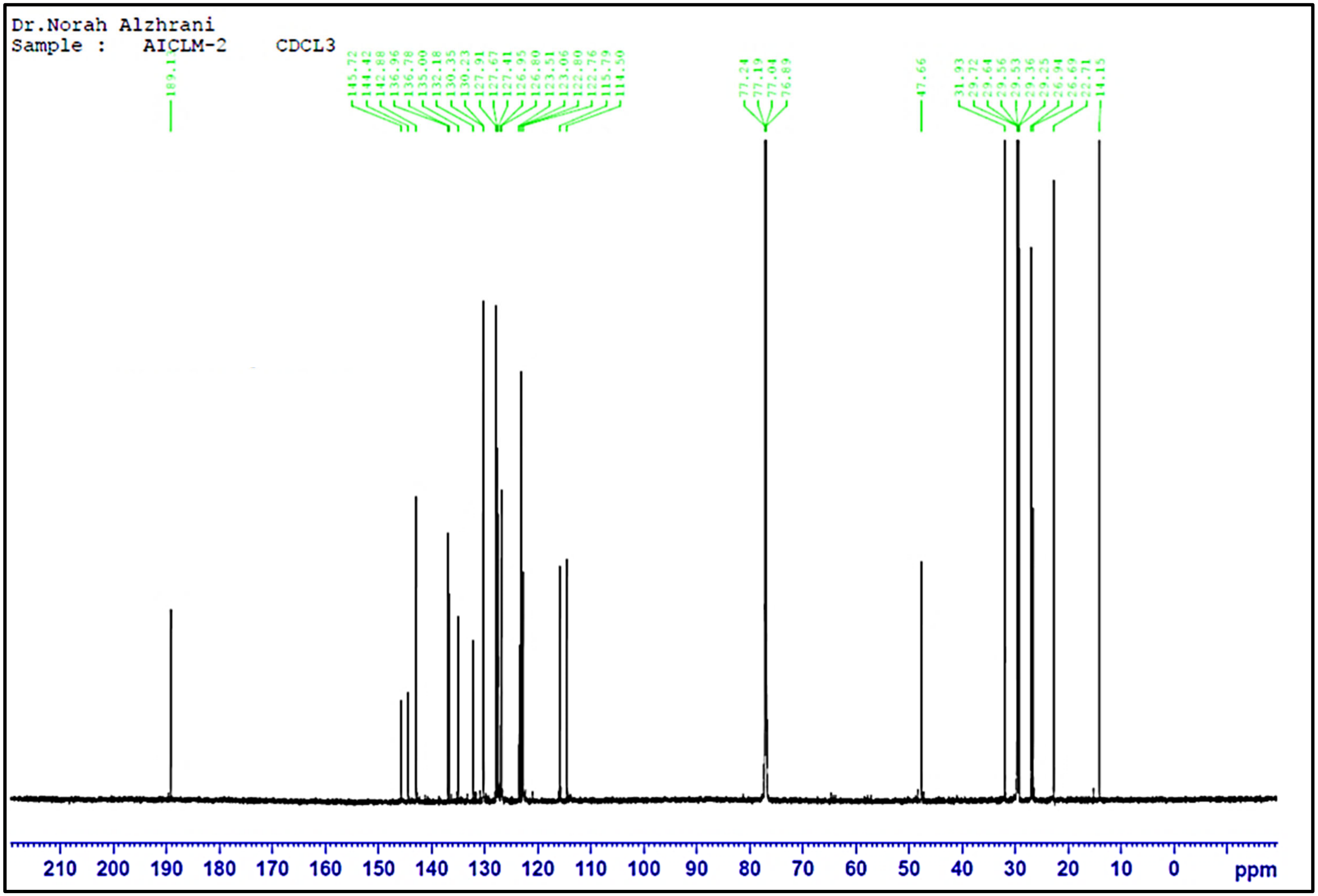

S33. ^{13}C NMR of 3-(3-Chloro-phenyl)-1-(*10-dodecylphenothiazin-2-yl*)-propenone (4f).

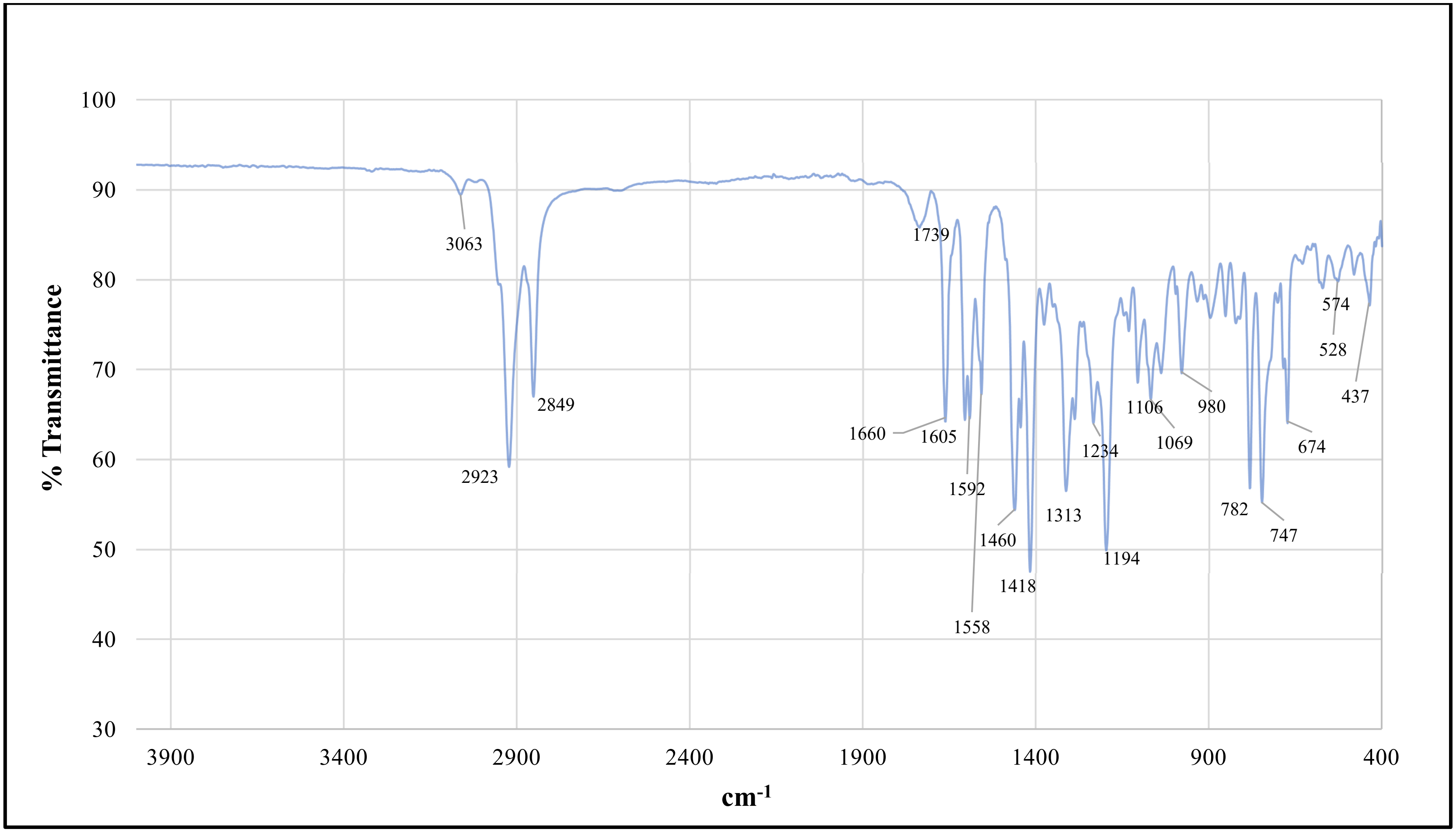

S34. IR of 3-(3-Chloro-phenyl)-1-(*10-dodecylphenothiazin-2-yl*)-propenone (4f).

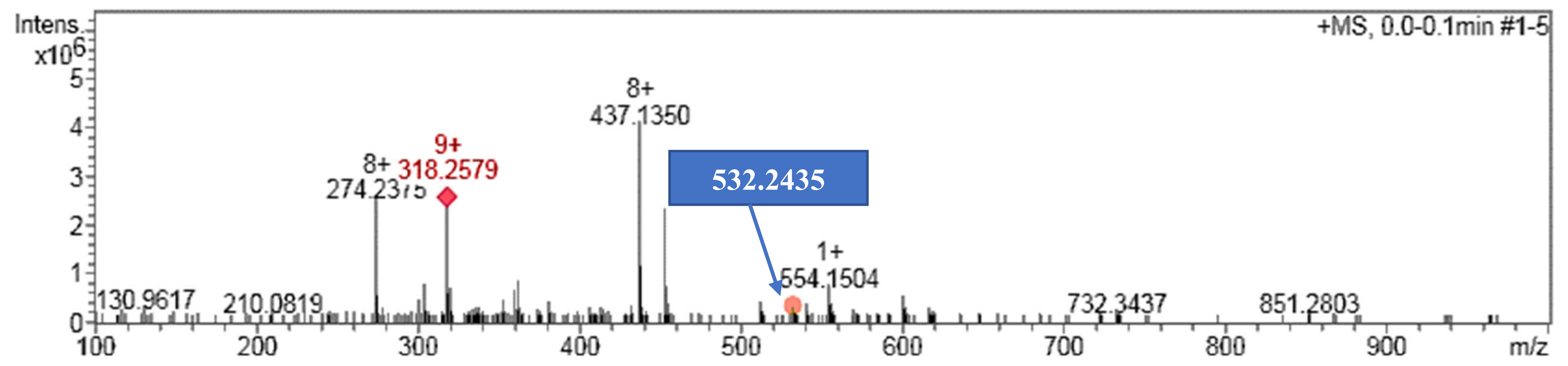

Meas. m/z	#	Ion Formula	m/z	err [ppm]	Mean err [ppm]	rdb	N-Rule	e⁻ Conf	mSigma	Std I	Std Mean m/z	Std I VarNorm	Std m/z Diff	Std Comb Dev
532.171118	1	C33H39ClNOS	532.243540	136.1	137.4	14.5	ok	even	23.1	26.1	n.a.	n.a.	n.a.	n.a.

S35. MS of 3-(3-Chloro-phenyl)-1-(*10-dodecylphenothiazin-2-yl*)-propenone (4f).

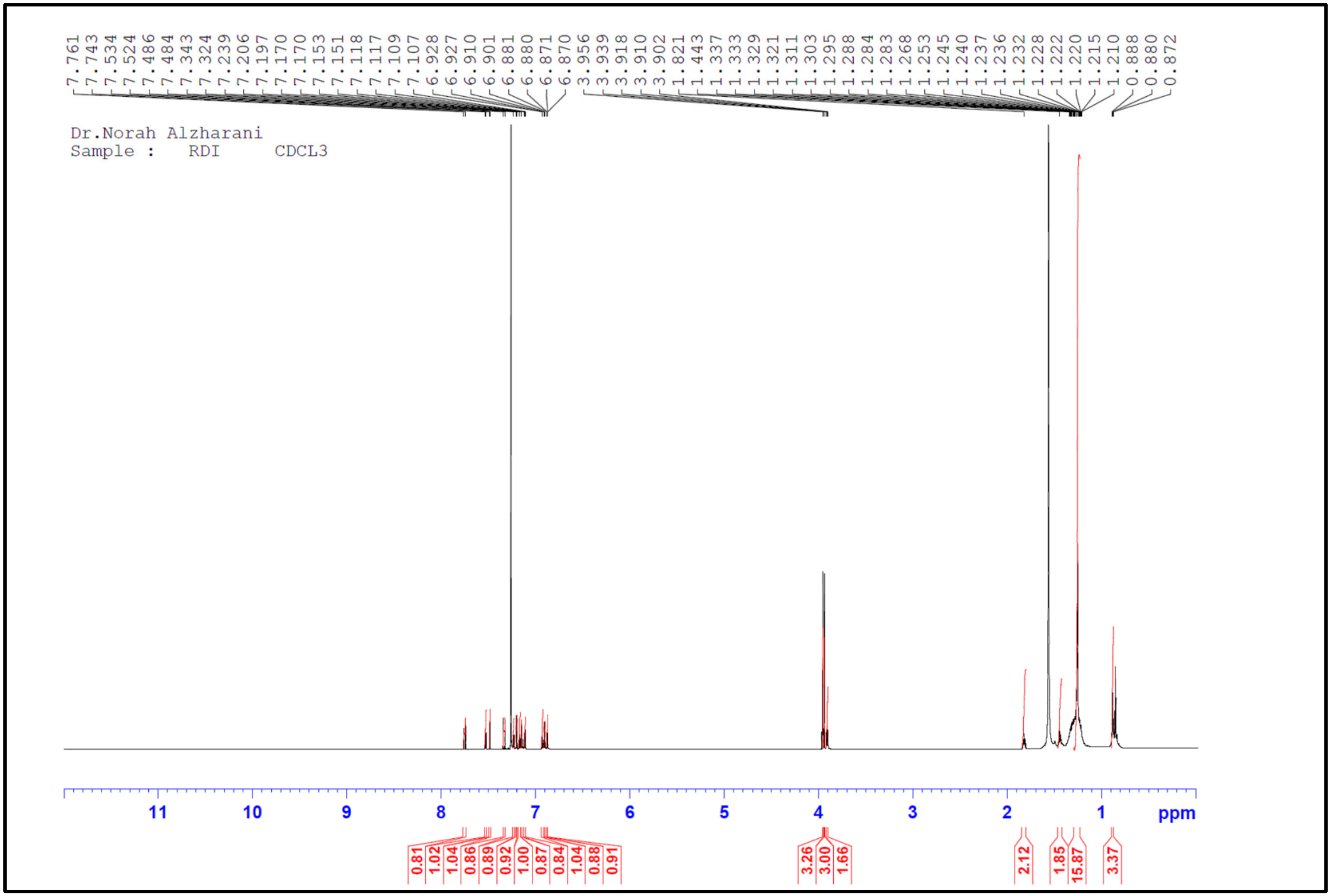

S36. ^{1}H NMR of 3-(3,4-Dimethoxy-phenyl)-1-(*10-dodecylphenothiazin-2-yl*)-propenone (4g).

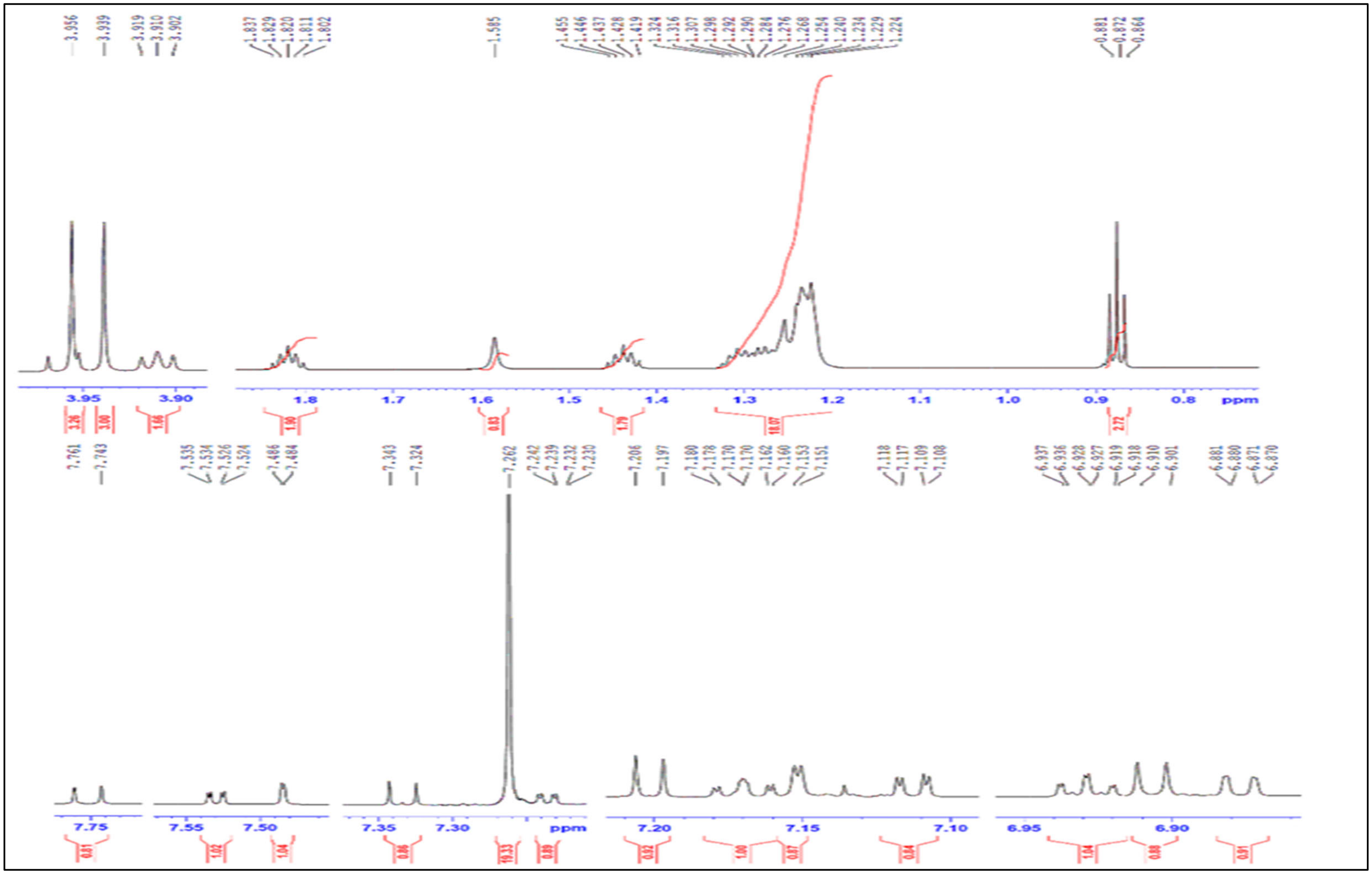

S37. ^{1}H NMR of 3-(3,4-Dimethoxy-phenyl)-1-(*10-dodecylphenothiazin-2-yl*)-propenone (4g).

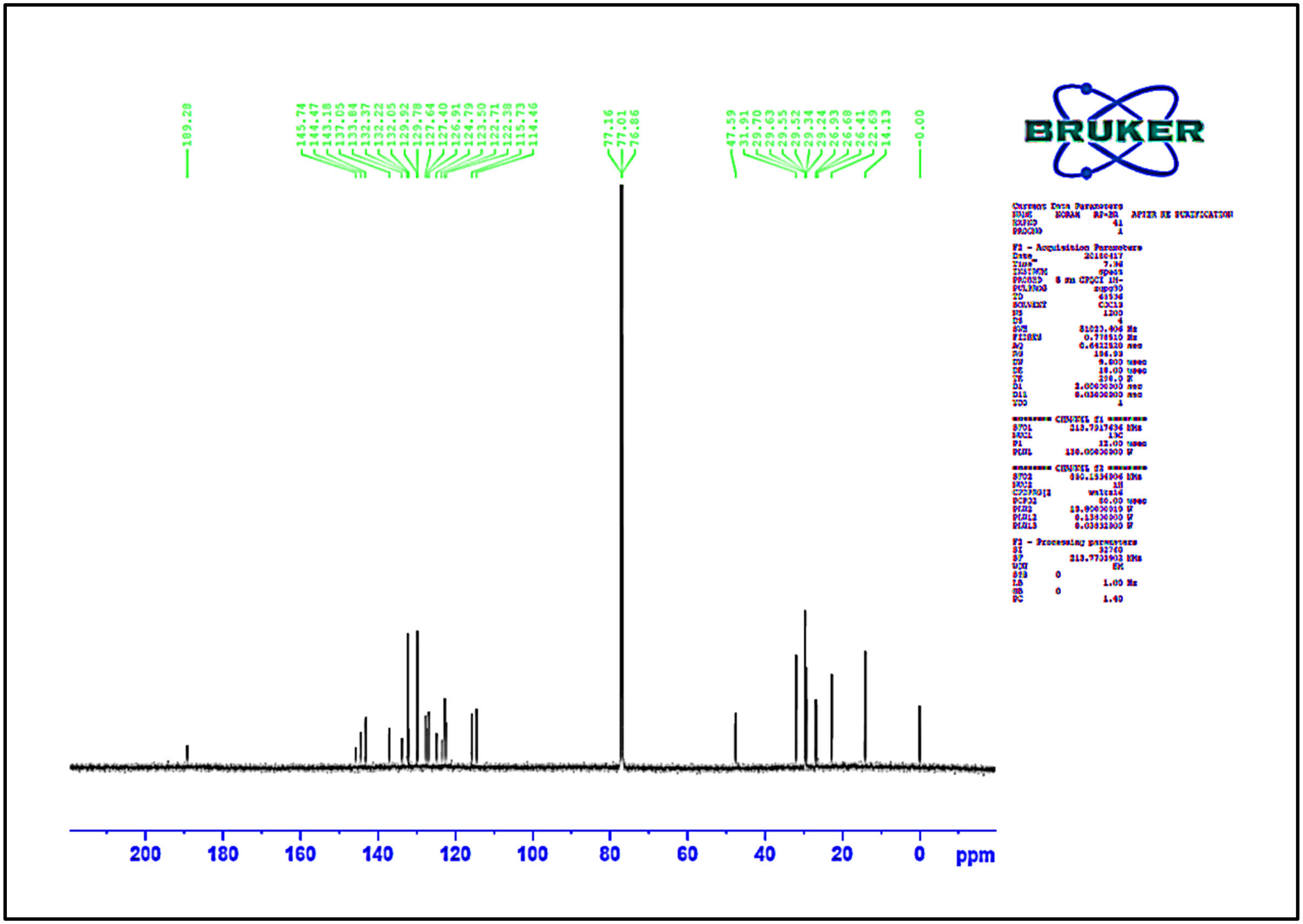

S38. ^{13}C NMR of 3-(3,4-Dimethoxy-phenyl)-1-(*10-dodecylphenothiazin-2-yl*)-propenone (4g).

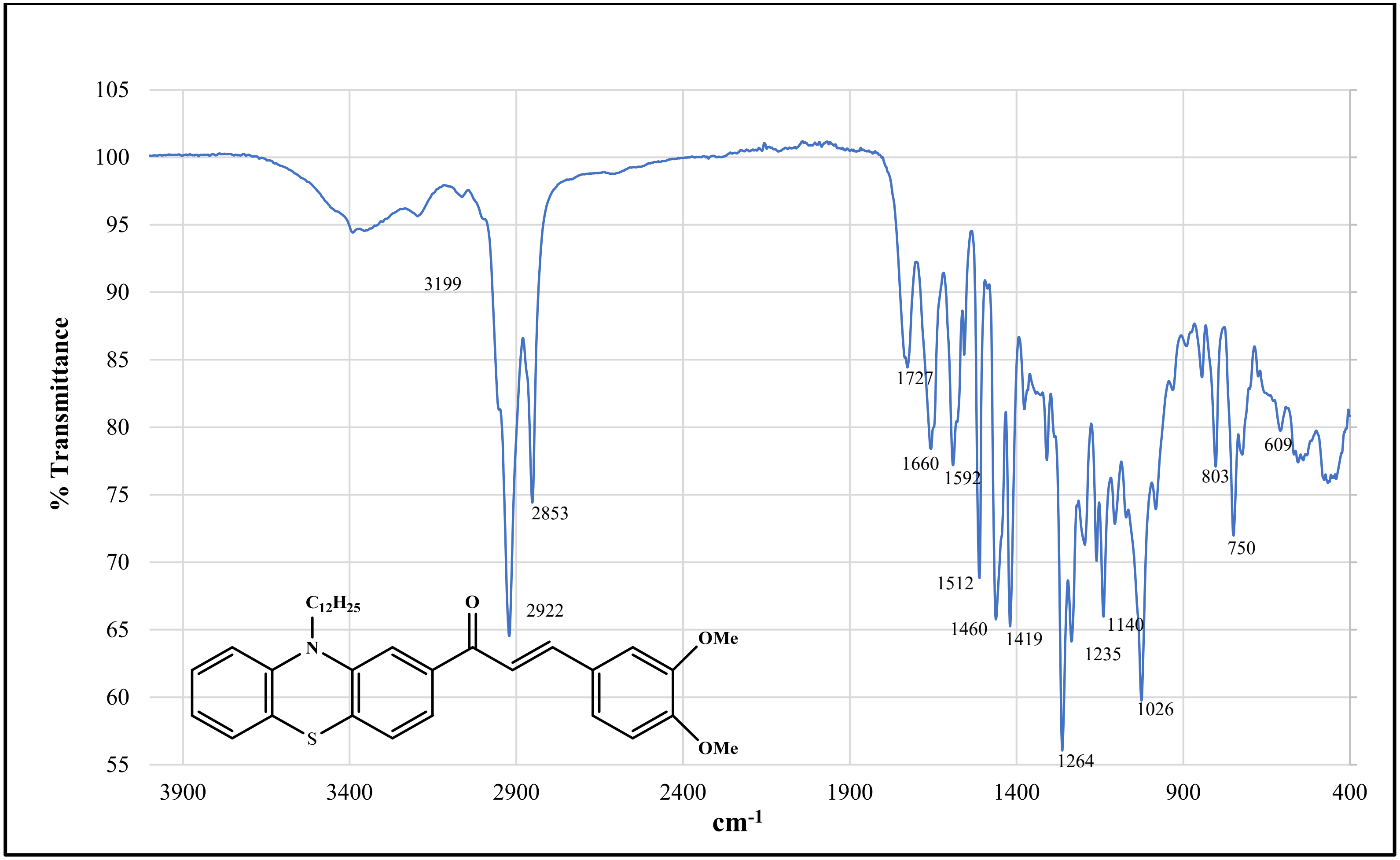

S39. IR of 3-(3,4-Dimethoxy-phenyl)-1-(*10-dodecylphenothiazin-2-yl*)-propenone (4g).

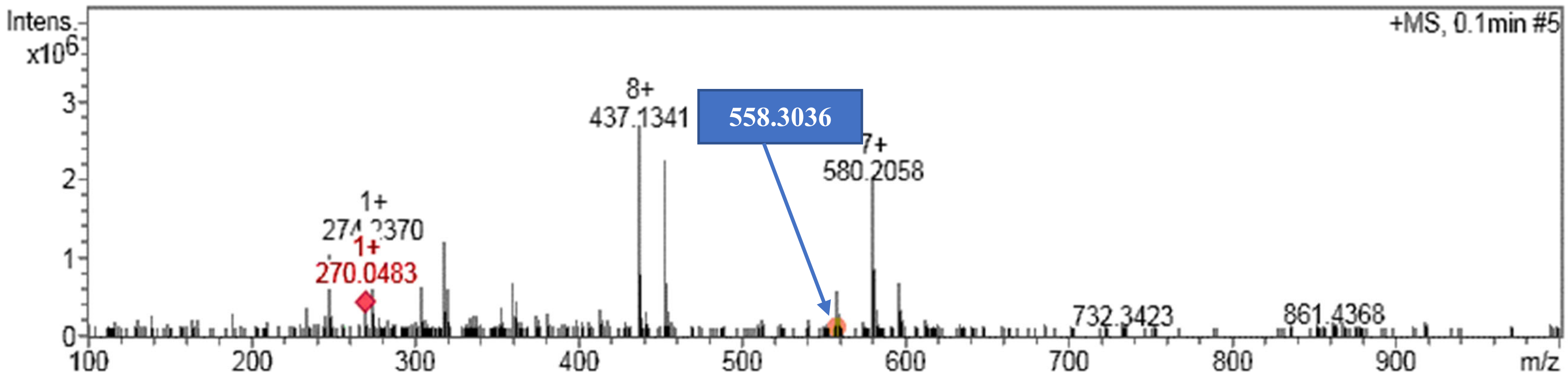

Meas. m/z	#	Ion Formula	m/z	err [ppm]	Mean err [ppm]	rdb	N-Rule	e⁻ Conf	mSigma	Std I	Std Mean m/z	Std I VarNorm	Std m/z Diff	Std Comb Dev
557.814434	1	C35H44NO3S	558.303642	876.2	877.2	14.5	ok	even	664.5	533.5	n.a.	n.a.	n.a.	n.a.

S40. MS of 3-(3,4-Dimethoxy-phenyl)-1-(*10-dodecylphenothiazin-2-yl*)-propenone (4g).

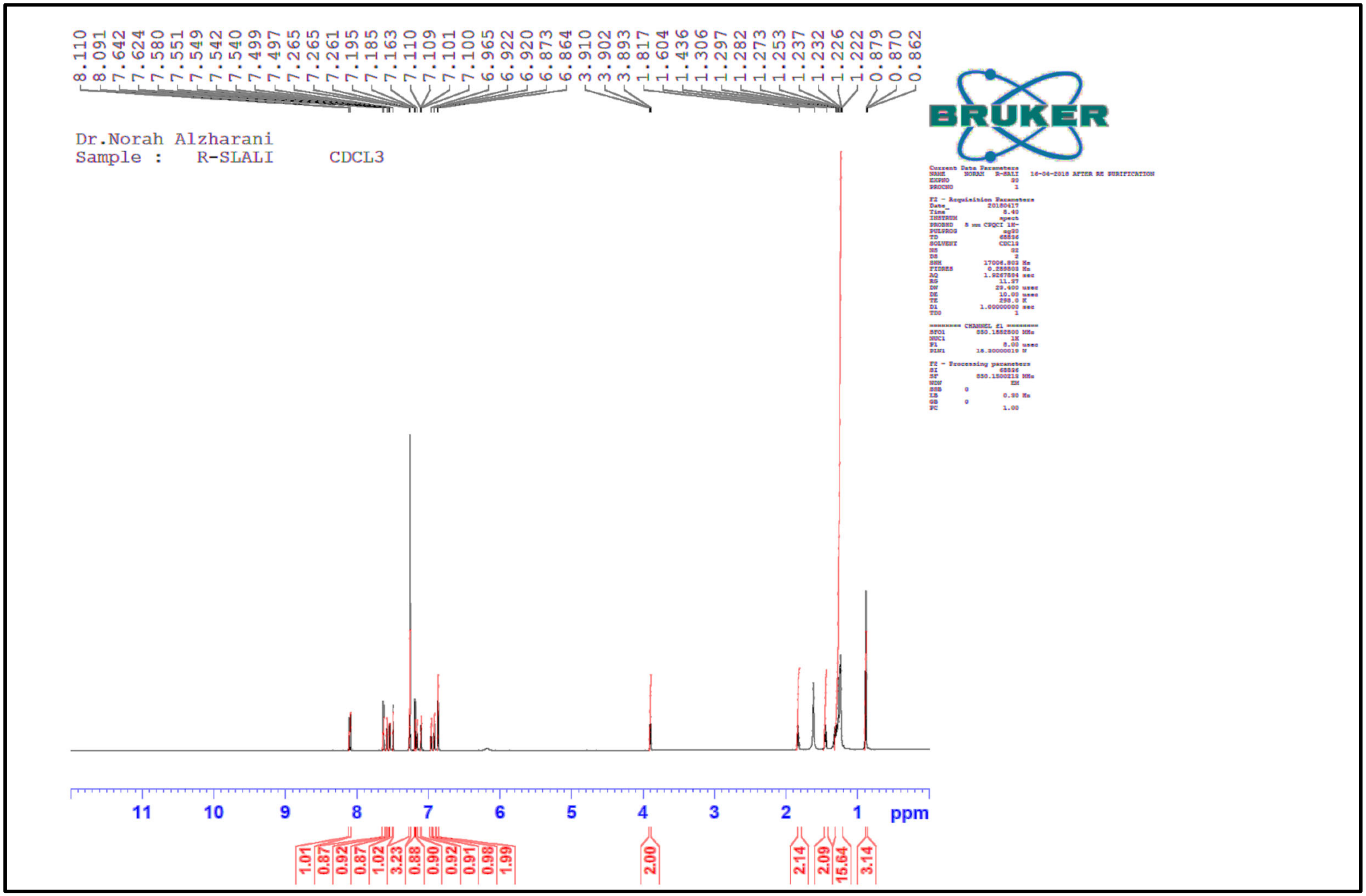

S41. [1]**H NMR of (E)-1-(***10-dodecylphenothiazin-2-yl***)-3-(2-hydroxyphenyl)prop-2-en-1-one (4h).**

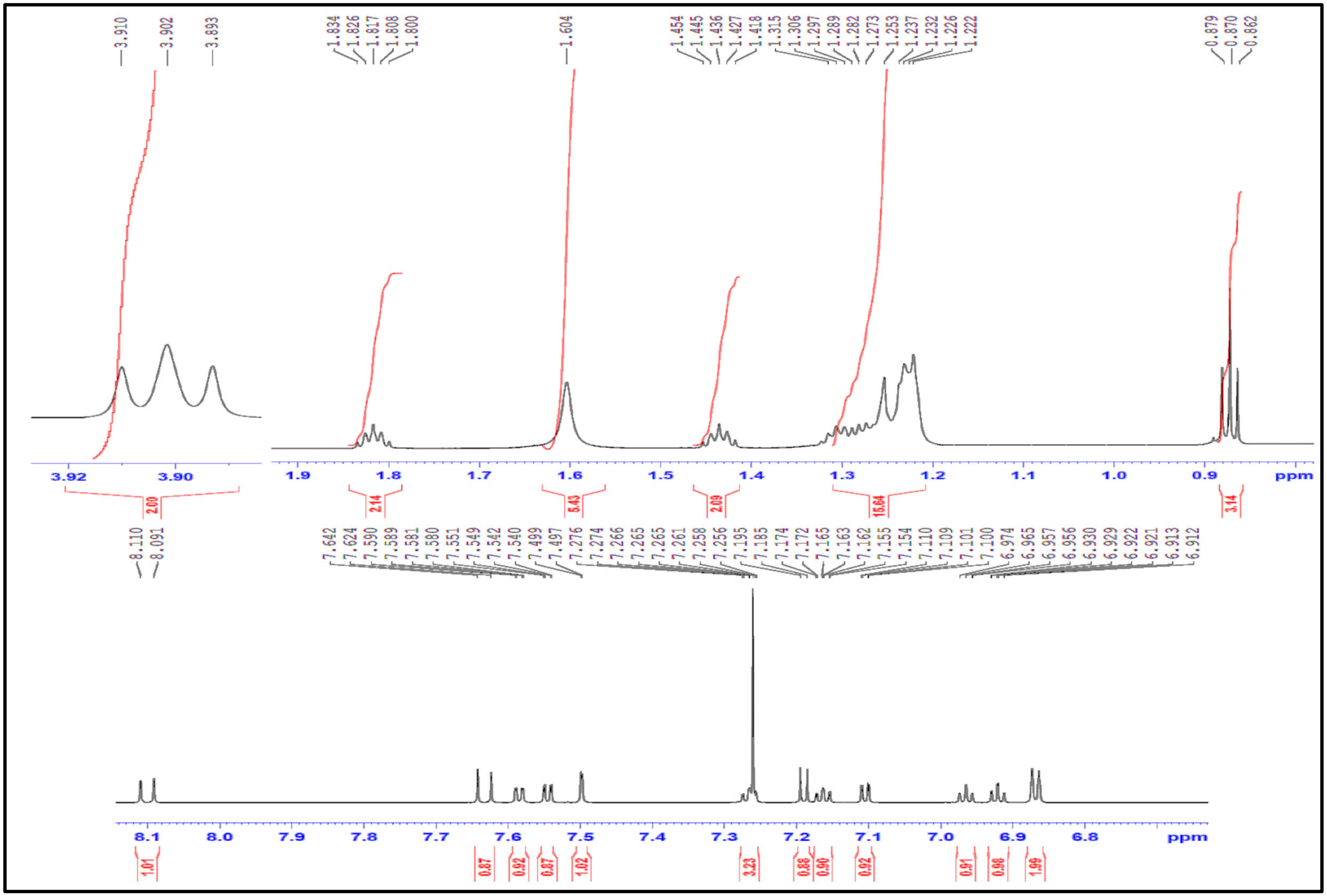

S42. ^{1}H NMR of (E)-1-(*10-dodecylphenothiazin-2-yl*)-3-(2-hydroxyphenyl)prop-2-en-1-one (4h).

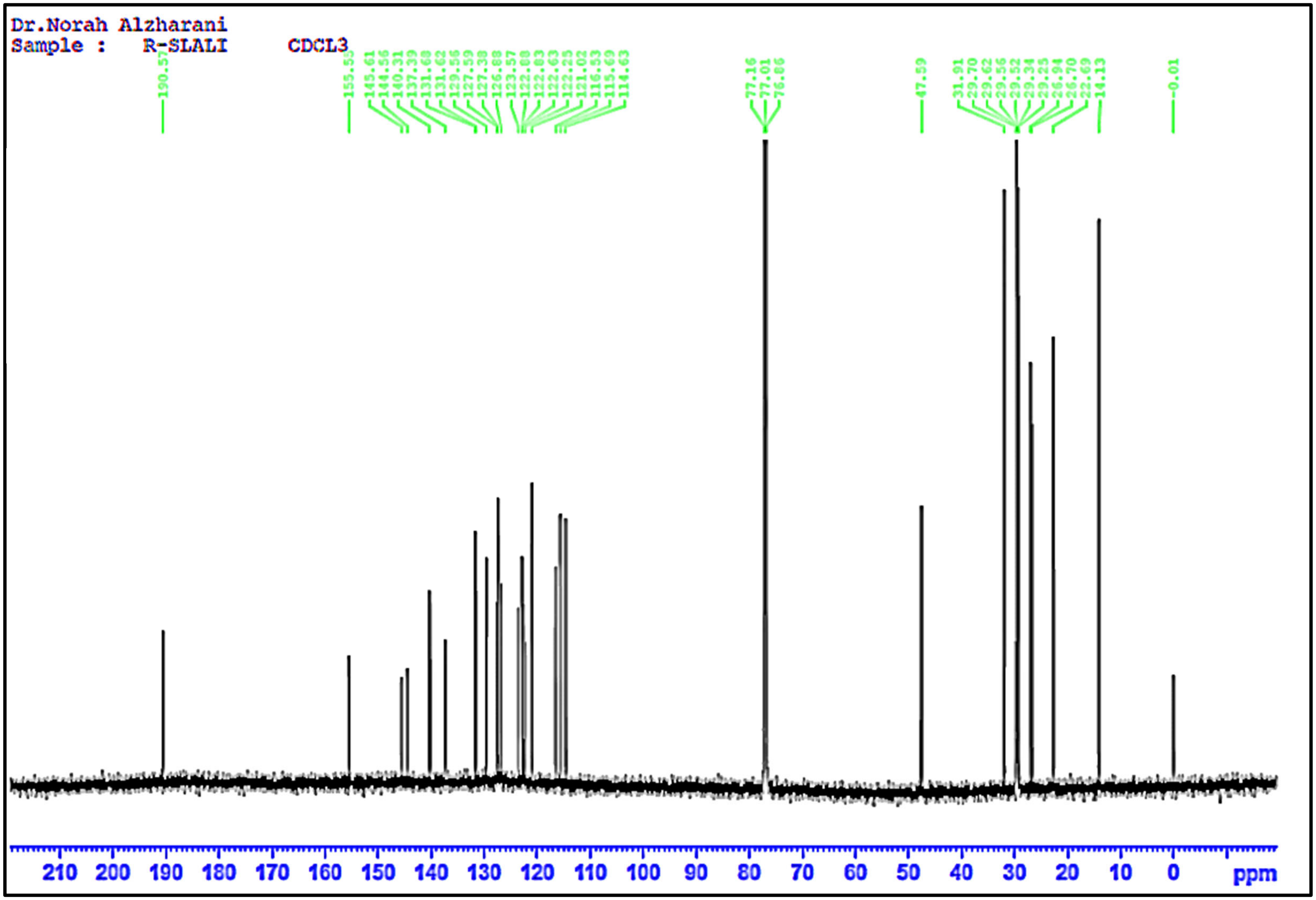

S43. ^{13}C NMR of (E)-1-(*10-dodecylphenothiazin-2-yl*)-3-(2-hydroxyphenyl)prop-2-en-1-one (4h).

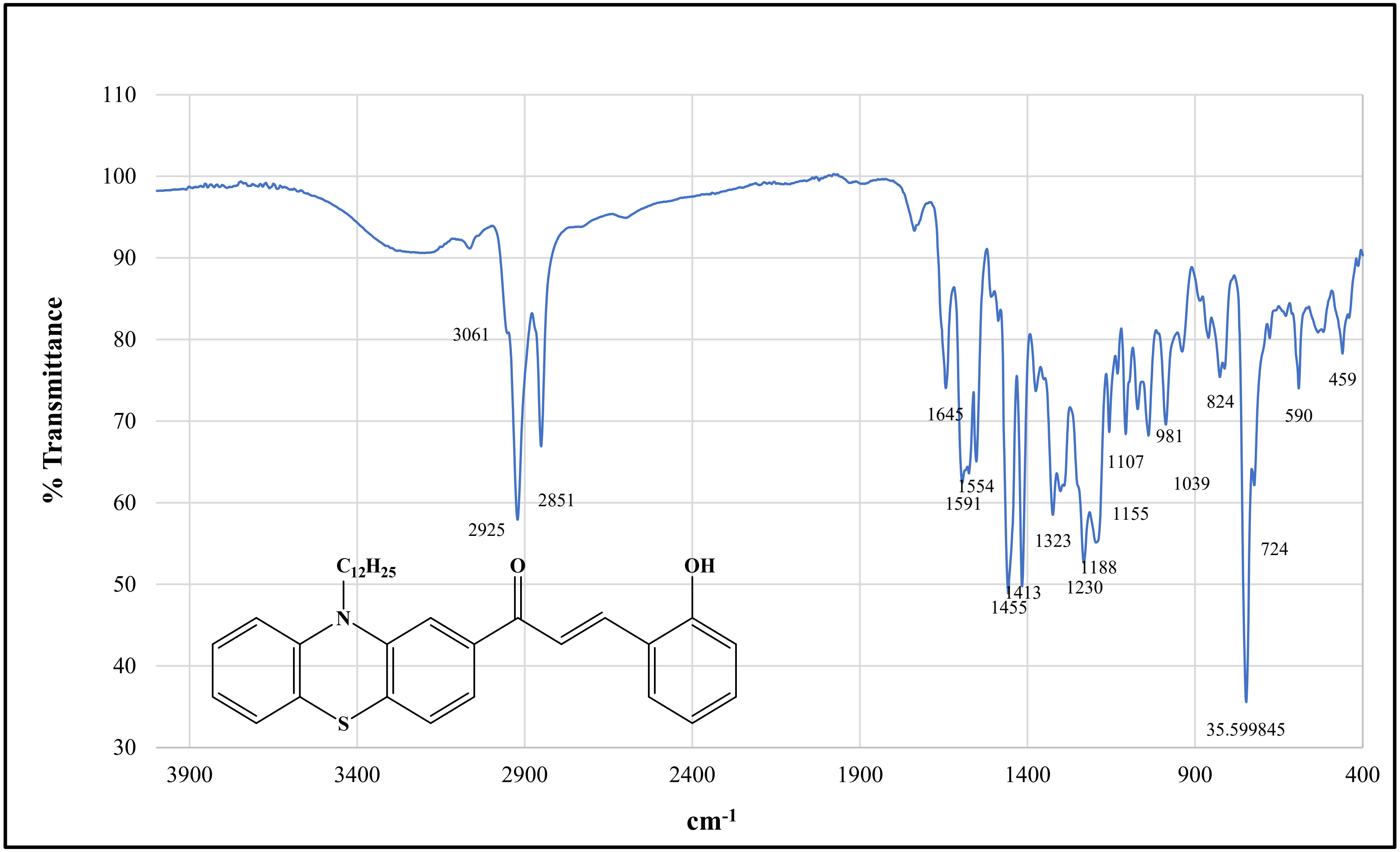

S44. IR of (E)-1-(*10-dodecylphenothiazin-2-yl*)-3-(2-hydroxyphenyl)prop-2-en-1-one (4h).

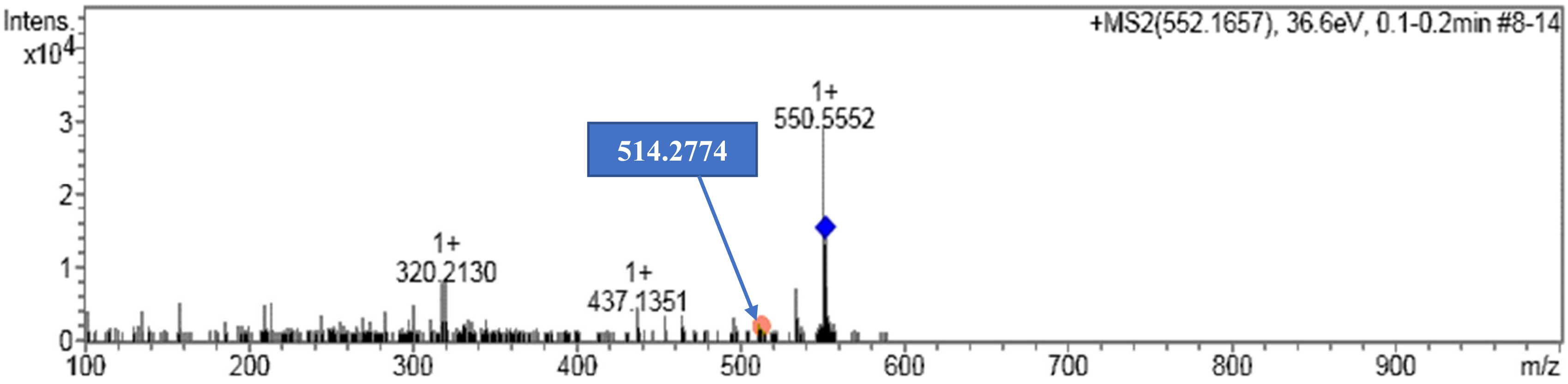

Meas. m/z	#	Ion Formula	m/z	err [ppm]	Mean err [ppm]	rdb	N-Rule	e⁻ Conf	mSigma	Std I	Std Mean m/z	Std I VarNorm	Std m/z Diff	Std Comb Dev
513.202286	1	C33H40NO2S	514.277427	2090.6	2091.4	14.5	ok	even	59.0	84.7	n.a.	n.a.	n.a.	n.a.

S45. MS of (E)-1-(*10-dodecylphenothiazin-2-yl*)-3-(2-hydroxyphenyl)prop-2-en-1-one (4h).

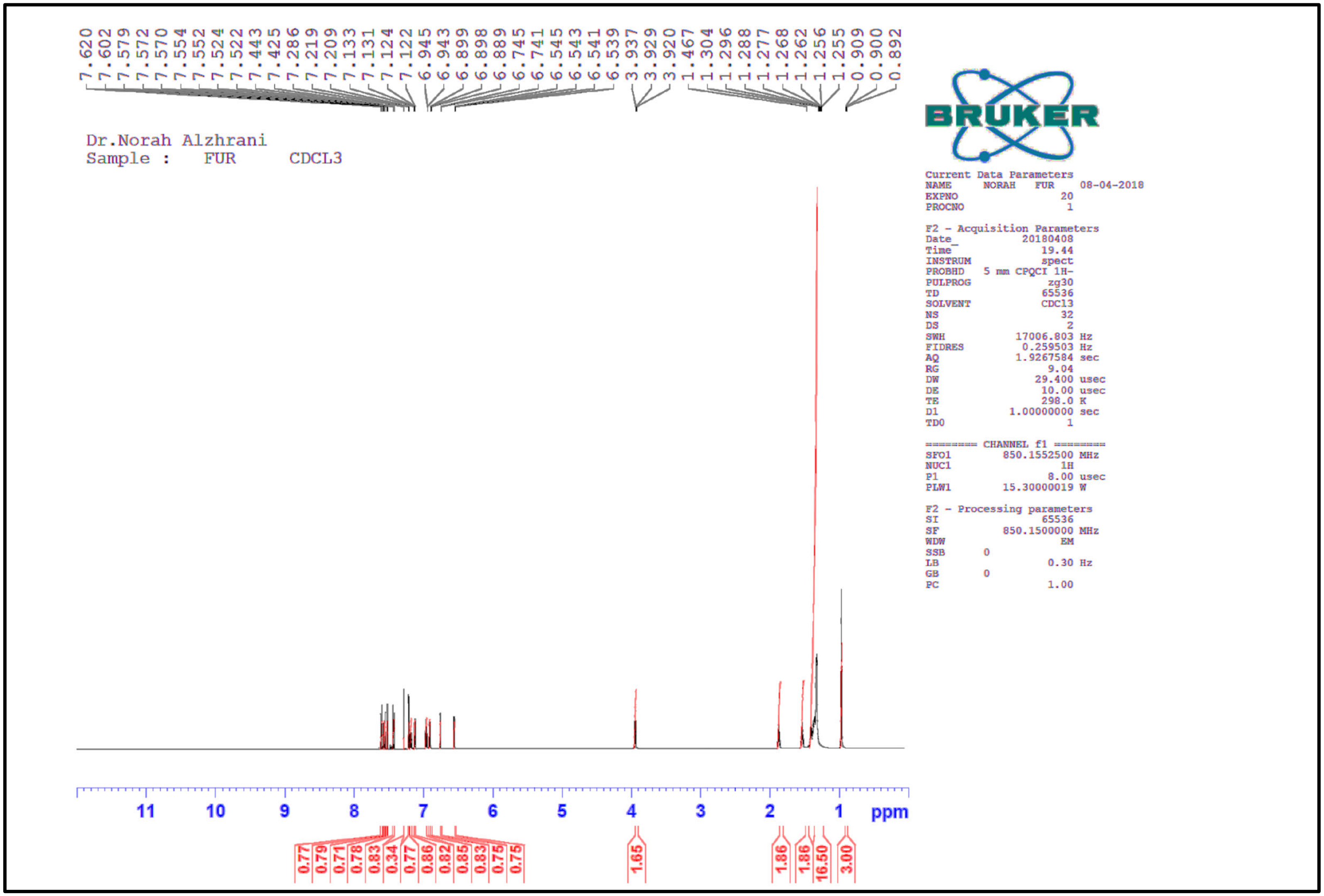

S46. ^{1}H NMR of (E)-1-(*10-dodecylphenothiazin-2-yl*)-3-(furan-2-yl)prop-2-en-1-one (4i).

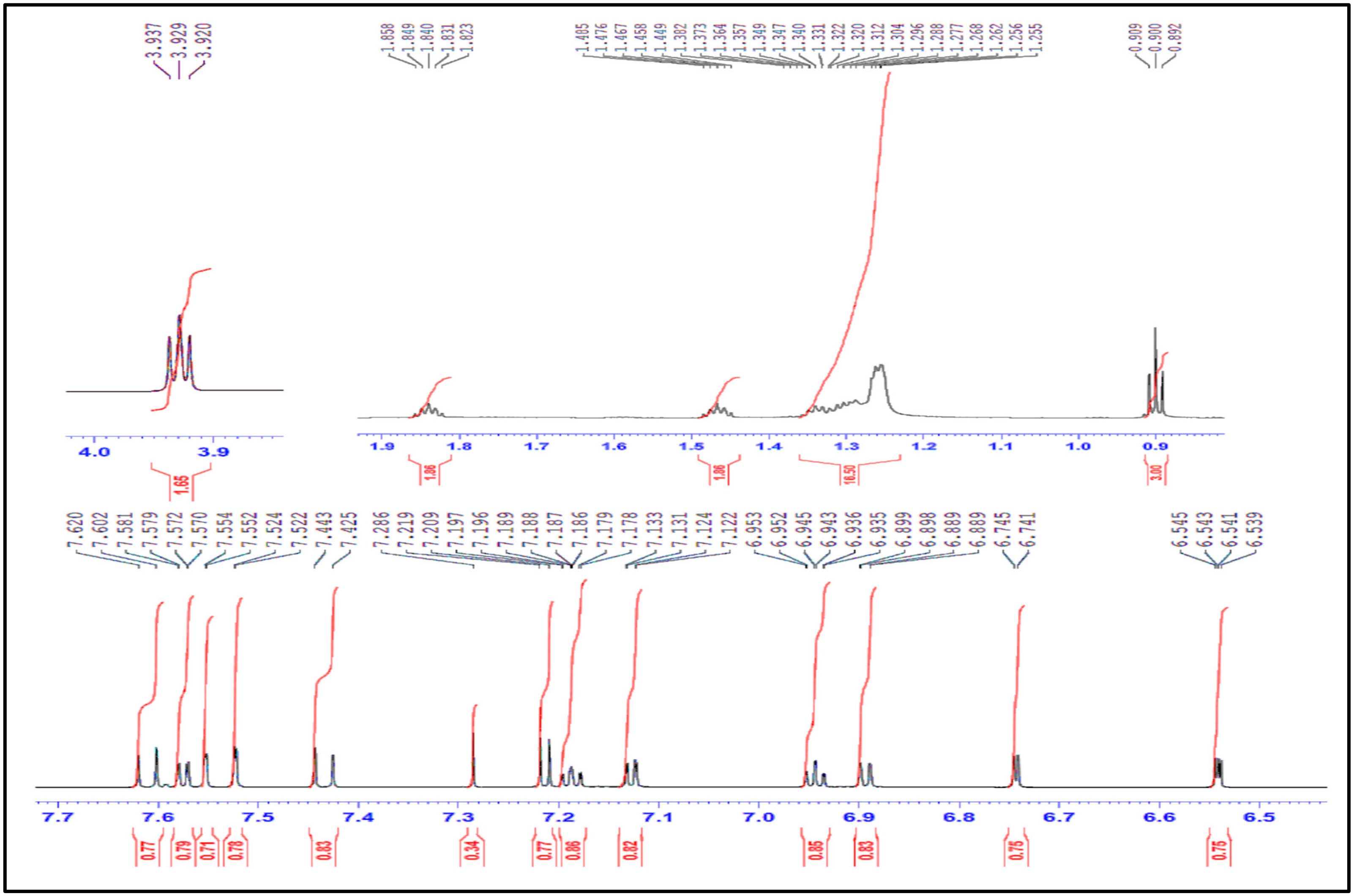

S47. ¹H NMR of (E)-1-(*10-dodecylphenothiazin-2-yl*)-3-(furan-2-yl)prop-2-en-1-one (4i).

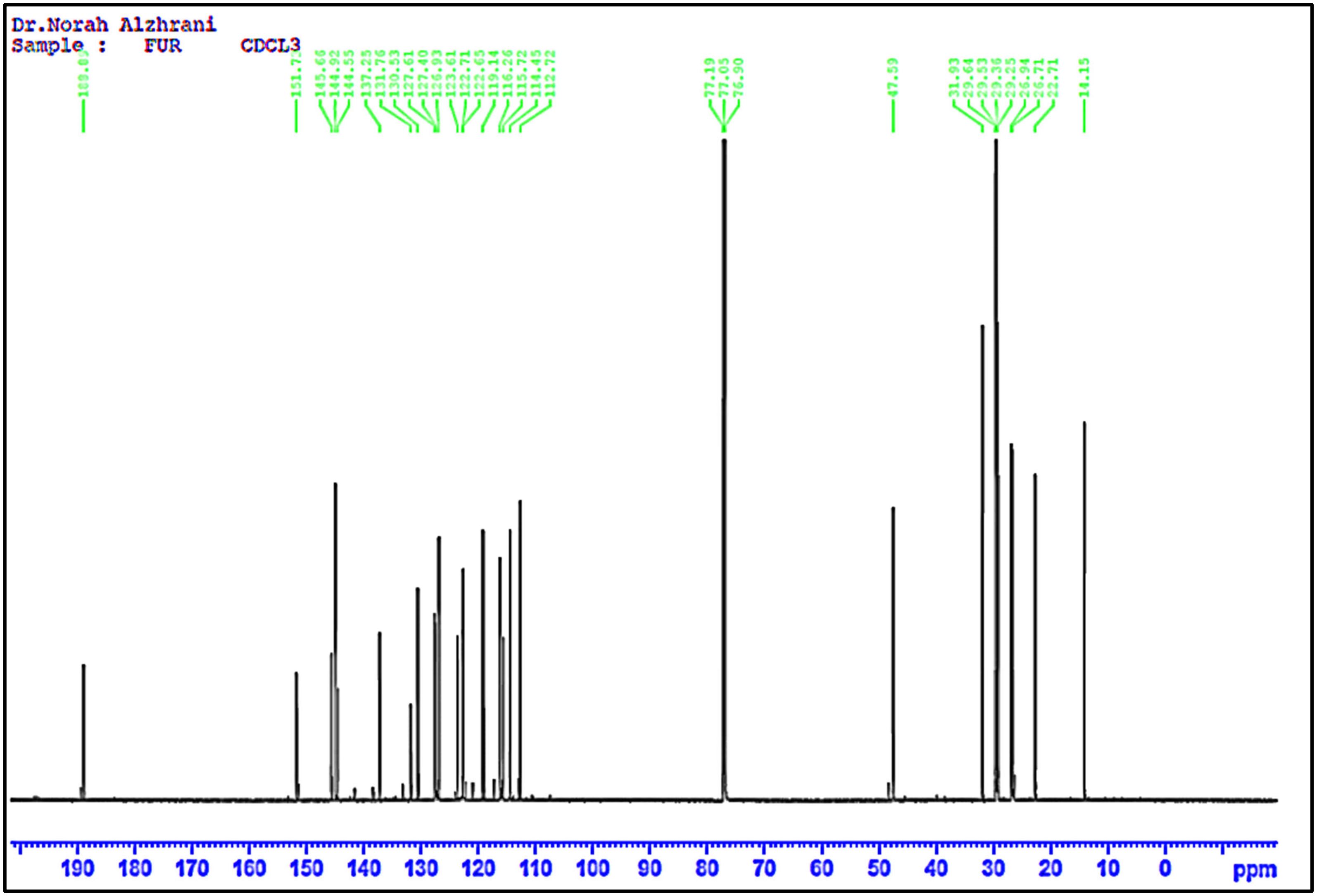

S48. ^{13}C NMR of (E)-1-(*10-dodecylphenothiazin-2-yl*)-3-(furan-2-yl)prop-2-en-1-one (4i).

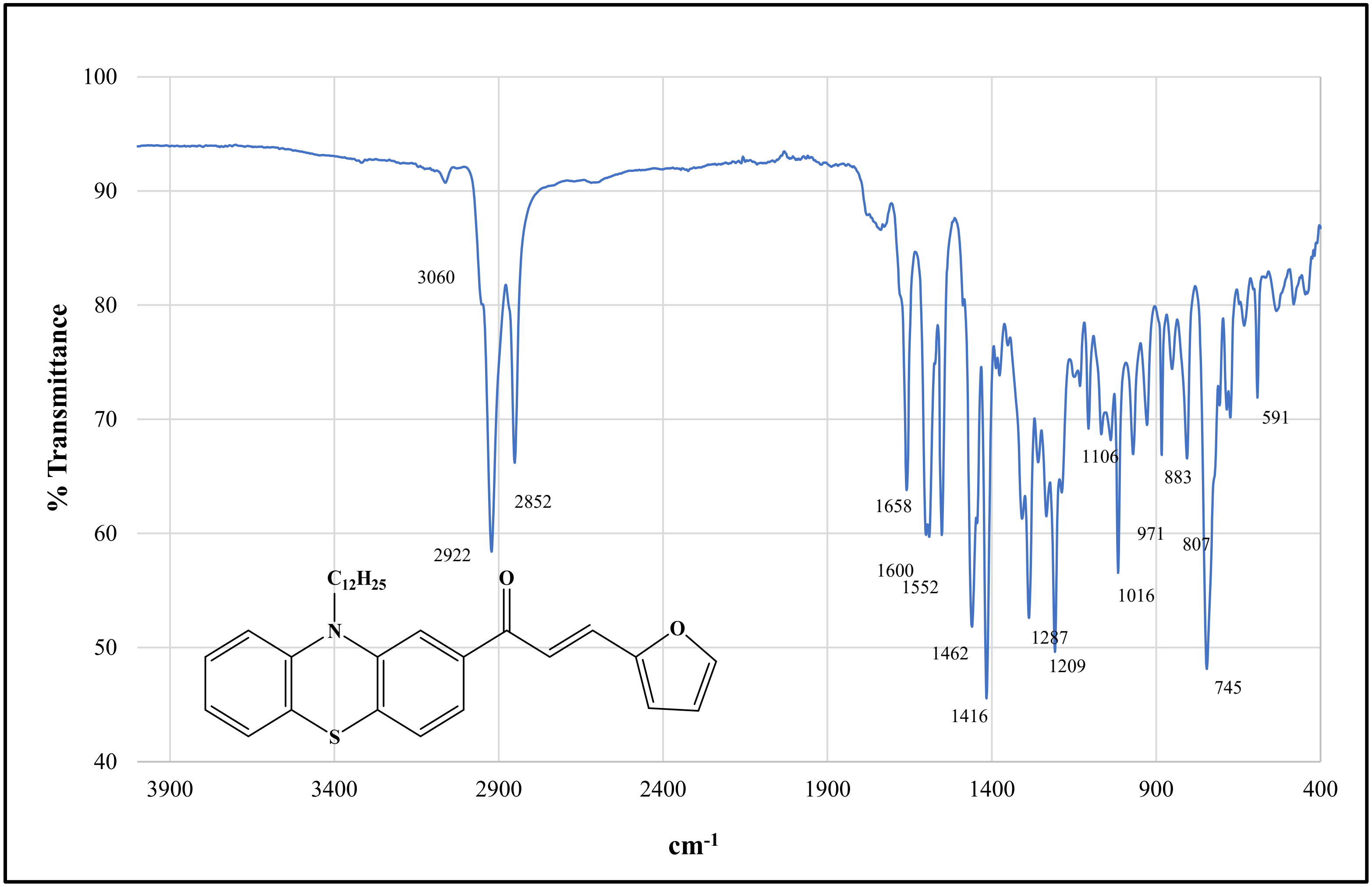

S49. IR of (E)-1-(*10-dodecylphenothiazin-2-yl*)-3-(furan-2-yl)prop-2-en-1-one (4i).

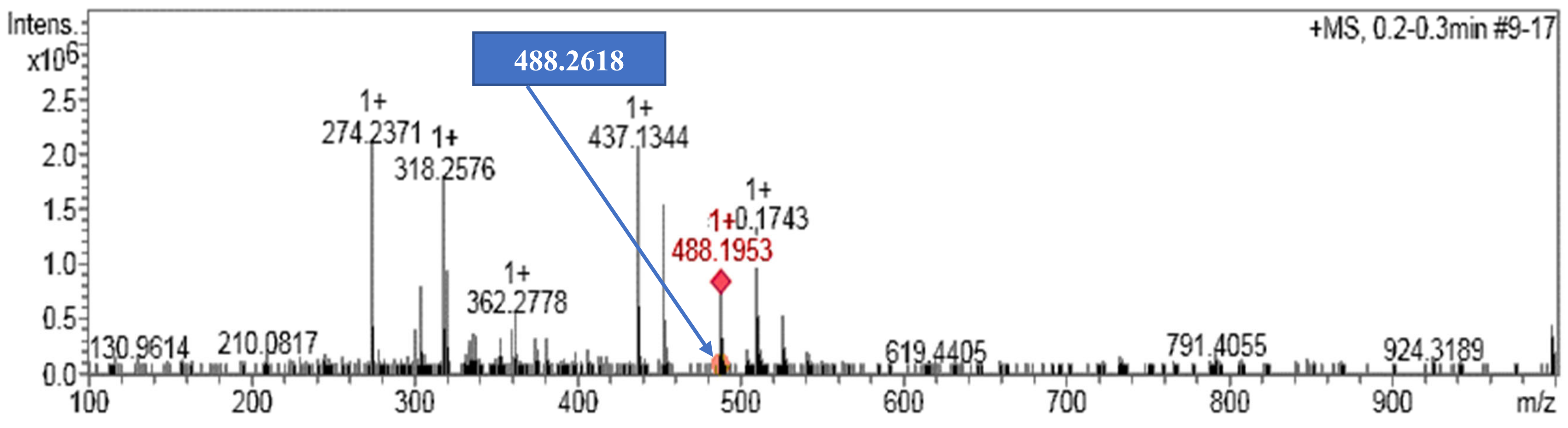

Meas. m/z	#	Ion Formula	m/z	err [ppm]	Mean err [ppm]	rdb	N-Rule	e⁻ Conf	mSigma	Std I	Std Mean m/z	Std I VarNorm	Std m/z Diff	Std Comb Dev
487.693749	1	C31H38NO2S	488.261777	1163.4	1149.9	13.5	ok	even	548.3	469.9	n.a.	n.a.	n.a.	n.a.

S50. MS of (E)-1-(*10-dodecylphenothiazin-2-yl*)-3-(furan-2-yl)prop-2-en-1-one (4i).

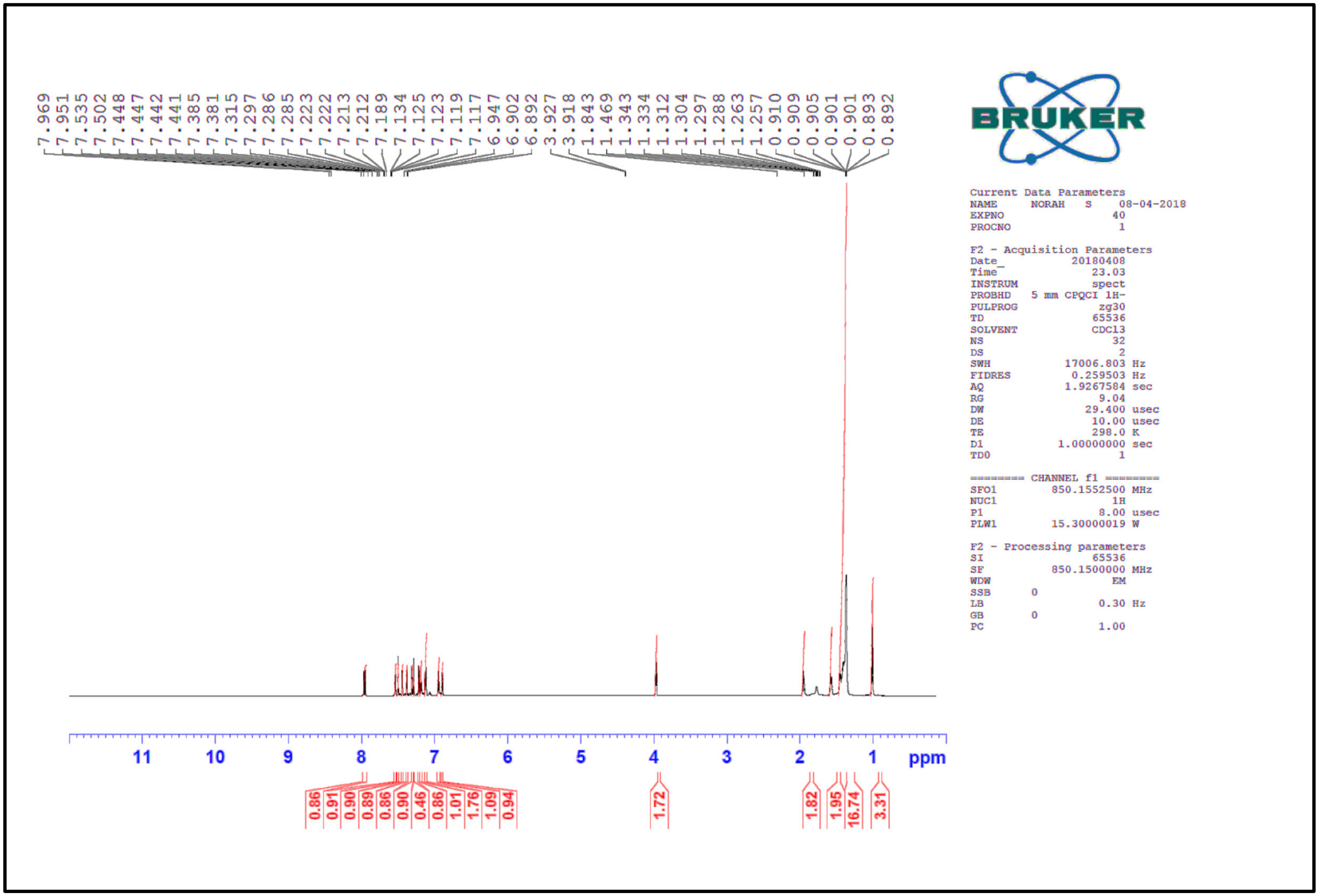

S51. ^{1}H NMR of (E)-1-(*10-dodecylphenothiazin-2-yl*)-3-(thiophen-3-yl)prop-2-en-1-one (4j).

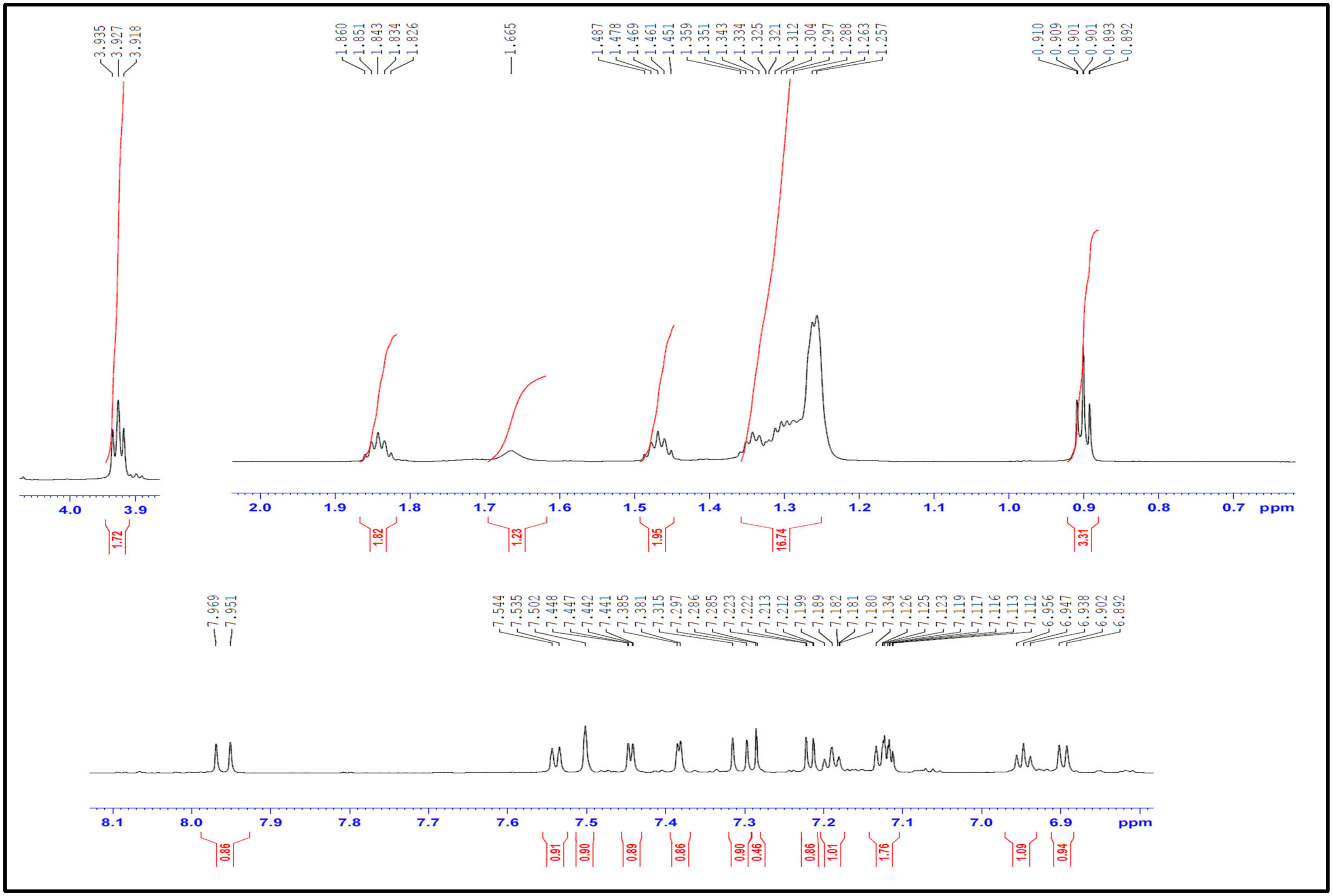

S52. ^{1}**H NMR of (E)-1-(*10-dodecylphenothiazin-2-yl*)-3-(thiophen-3-yl)prop-2-en-1-one (4j).**

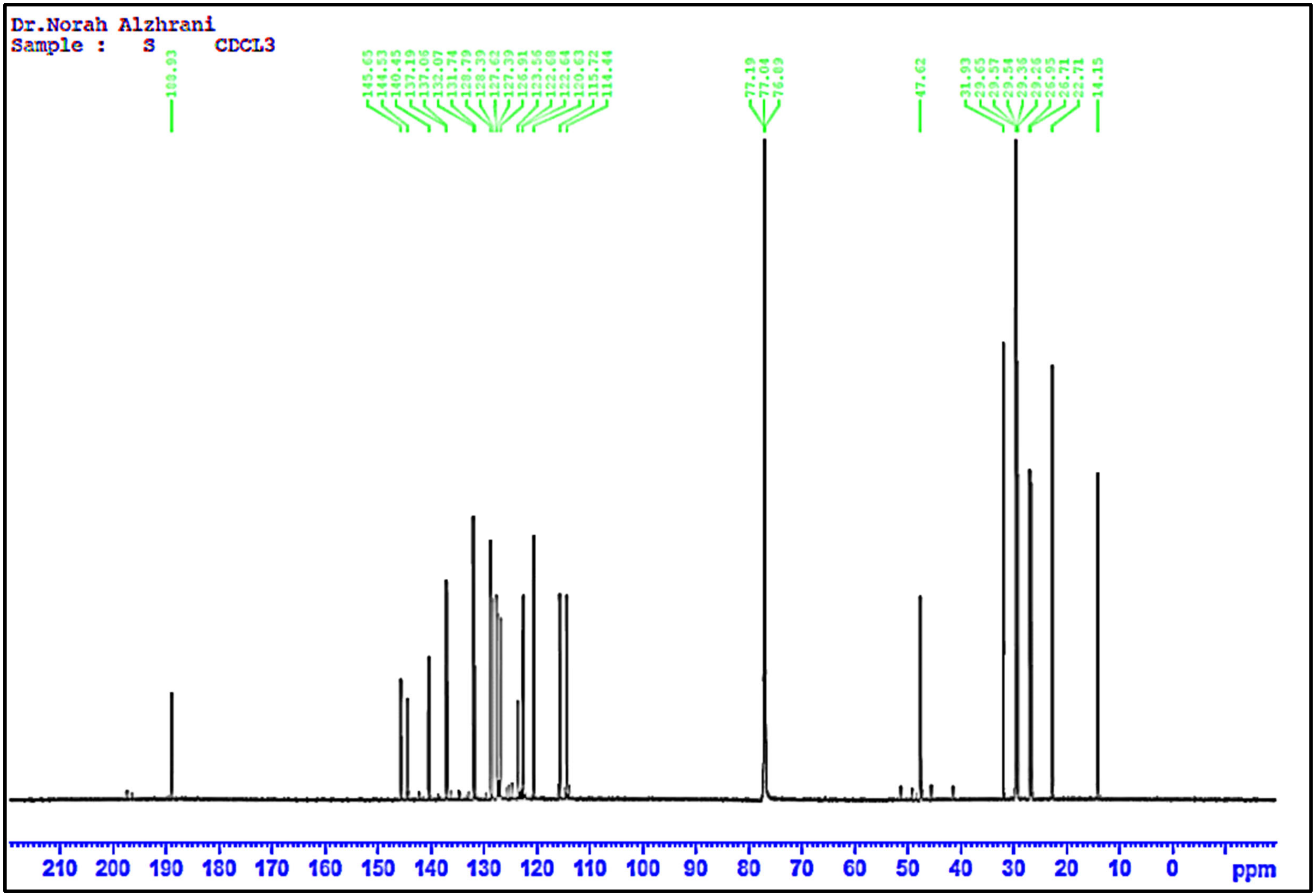

S53. ^{13}C NMR of (E)-1-(*10-dodecylphenothiazin-2-yl*)-3-(thiophen-3-yl)prop-2-en-1-one (4j).

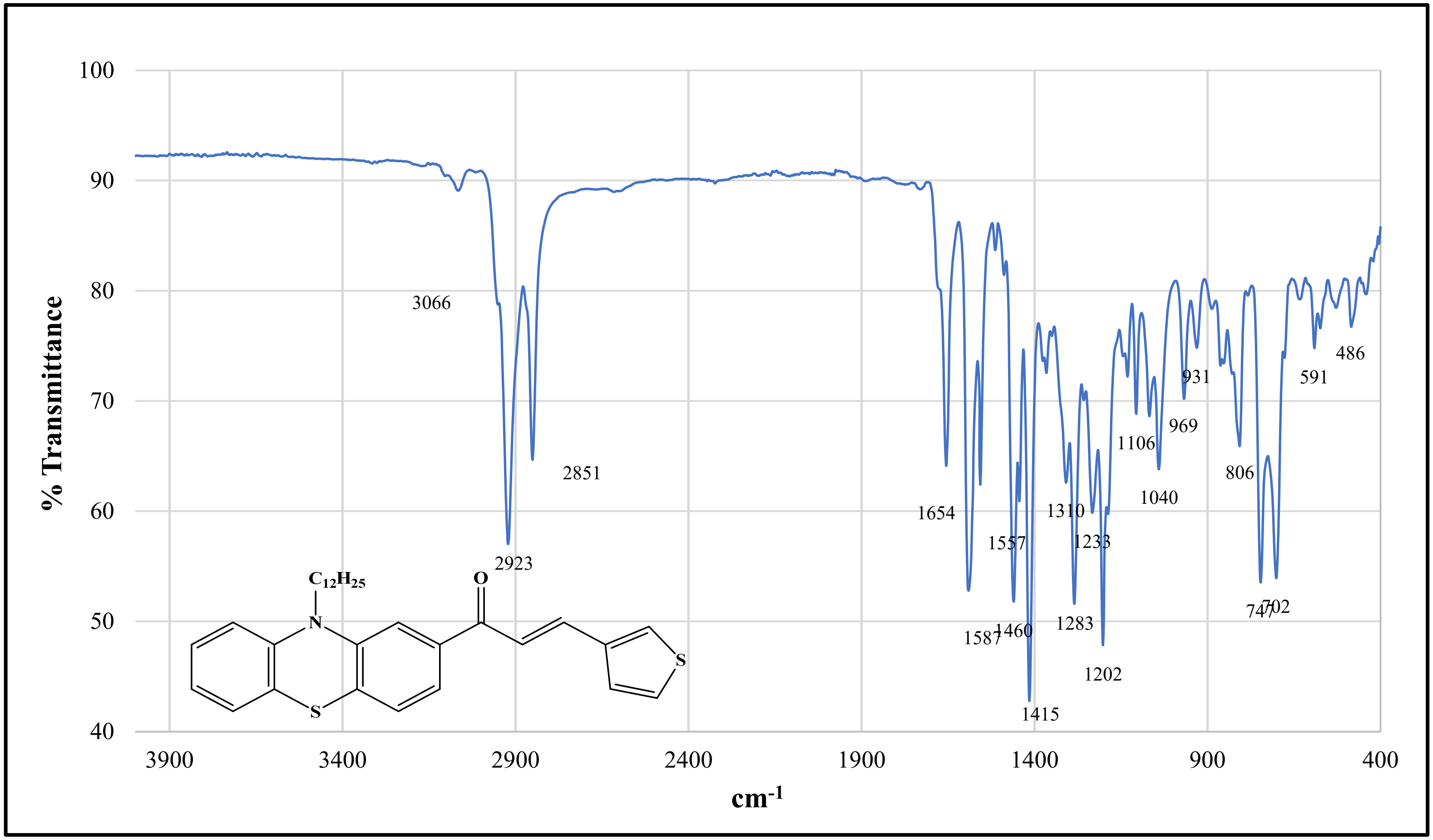

S54. IR of (E)-1-(*10-dodecylphenothiazin-2-yl*)-3-(thiophen-3-yl)prop-2-en-1-one (4j).

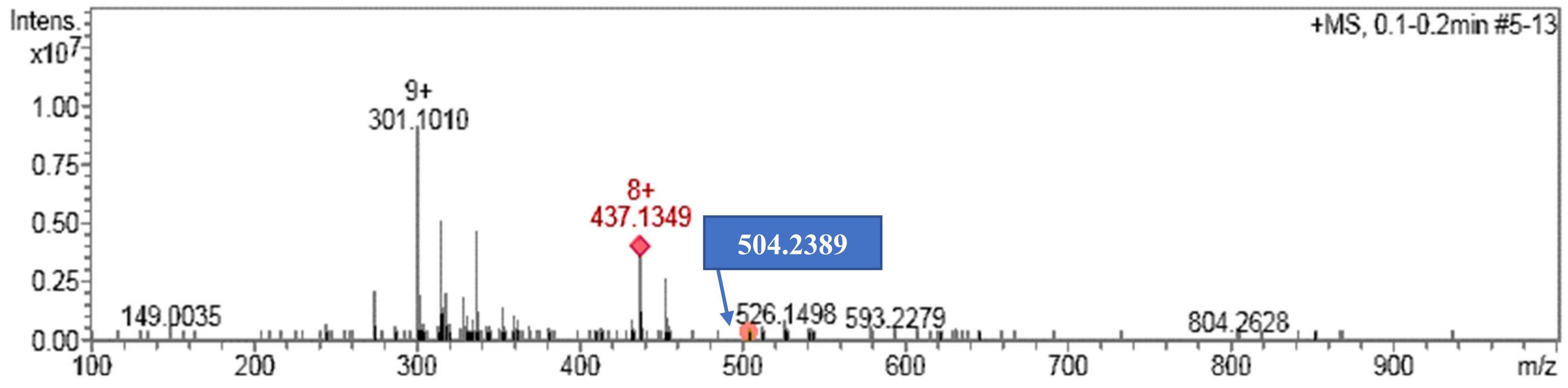

Meas. m/z	#	Ion Formula	m/z	err [ppm]	Mean err [ppm]	rdb	N-Rule	e⁻ Conf	mSigma	Std I	Std Mean m/z	Std I VarNorm	Std m/z Diff	Std Comb Dev
503.749784	1	C31H38NOS2	504.238933	970.1	984.5	13.5	ok	even	714.9	547.6	n.a.	n.a.	n.a.	n.a.

S55. MS of (E)-1-(*10-dodecylphenothiazin-2-yl*)-3-(thiophen-3-yl)prop-2-en-1-one (4j).

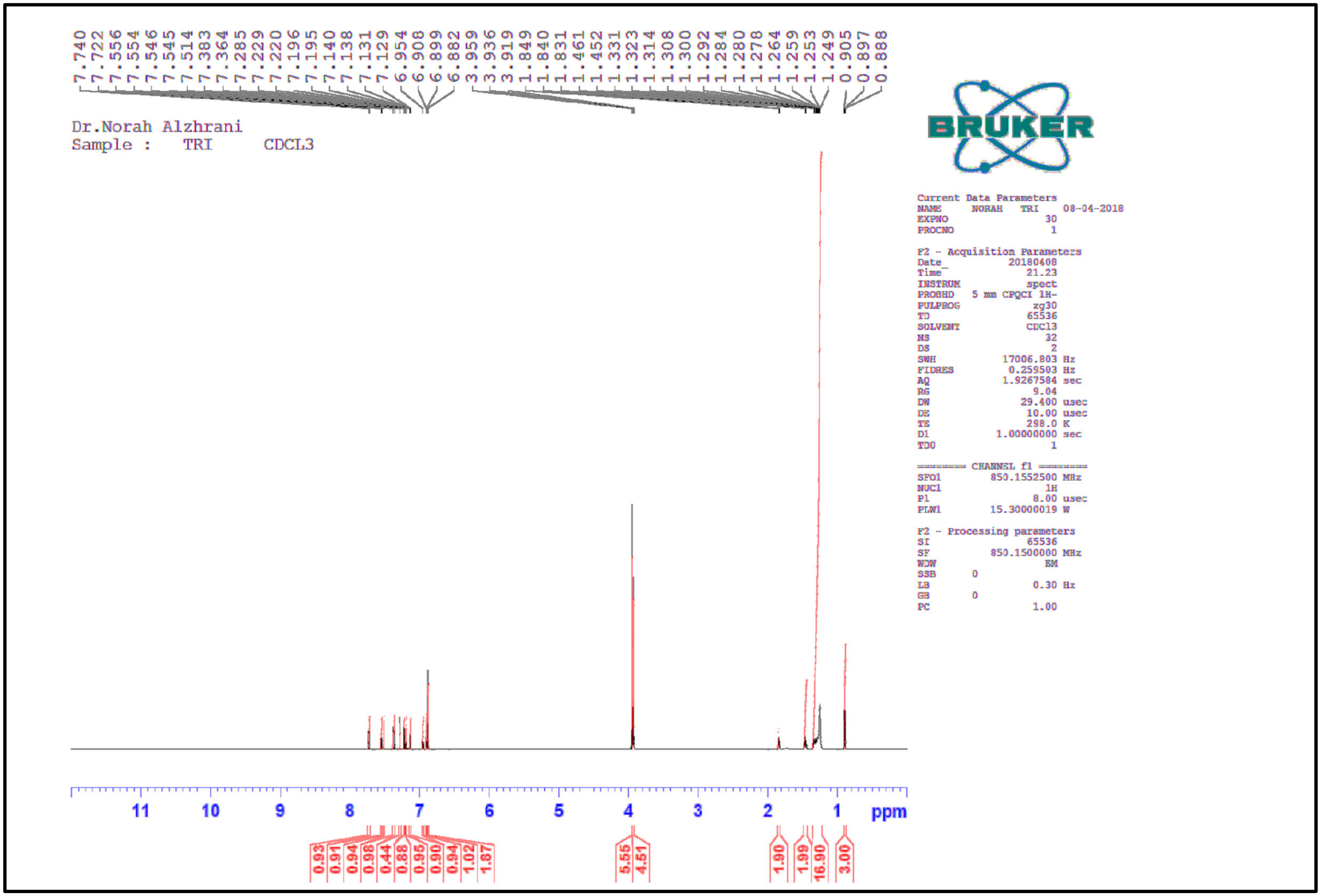

S56. ^{1}H NMR of (E)-1-(*10-dodecylphenothiazin-2-yl*)-3-(3,4,5-trimethoxyphenyl)prop-2-en-1-on (4k).

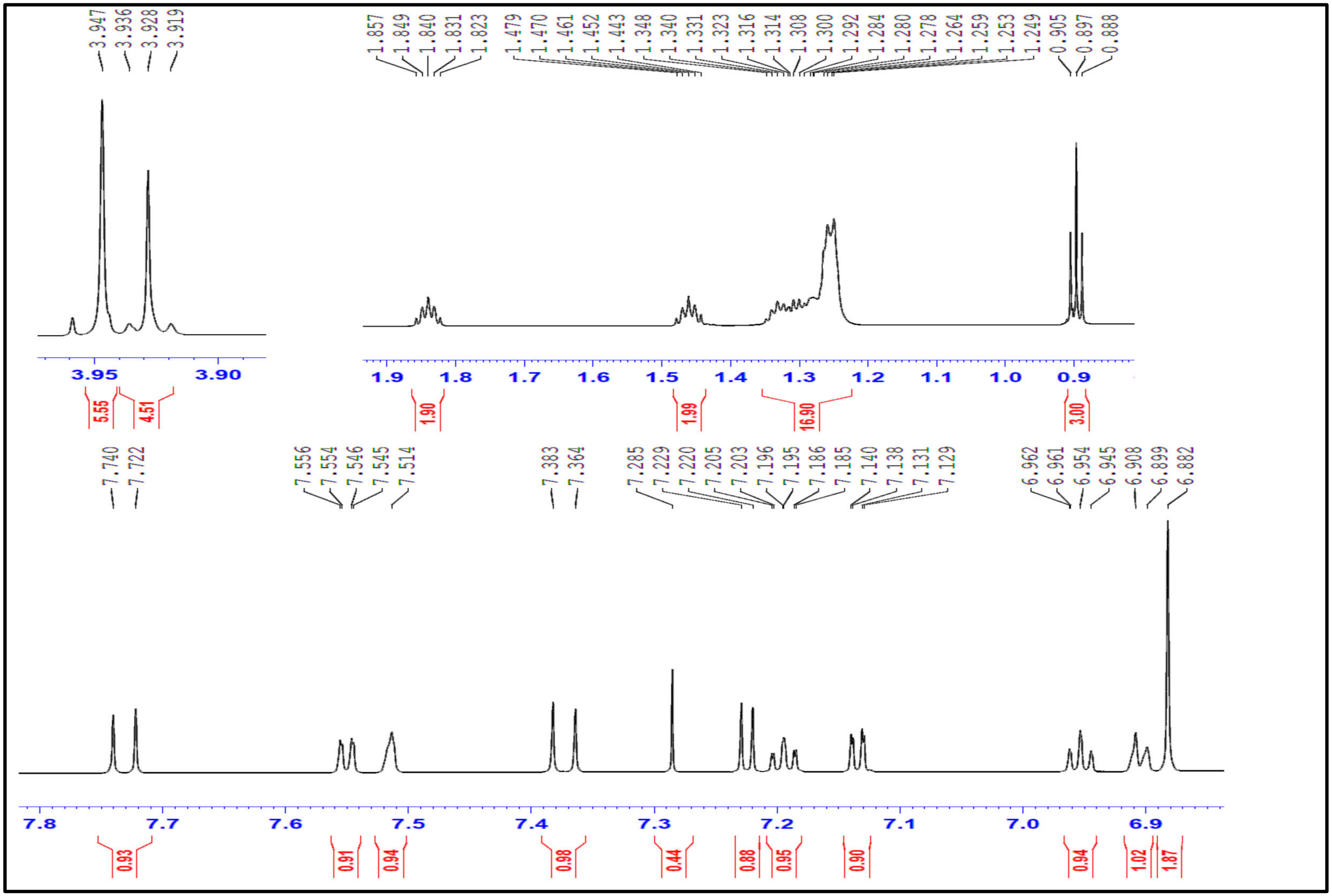

S57. ^{1}H NMR of (E)-1-(*10-dodecylphenothiazin-2-yl*)-3-(3,4,5-trimethoxyphenyl)prop-2-en-1-on (4k).

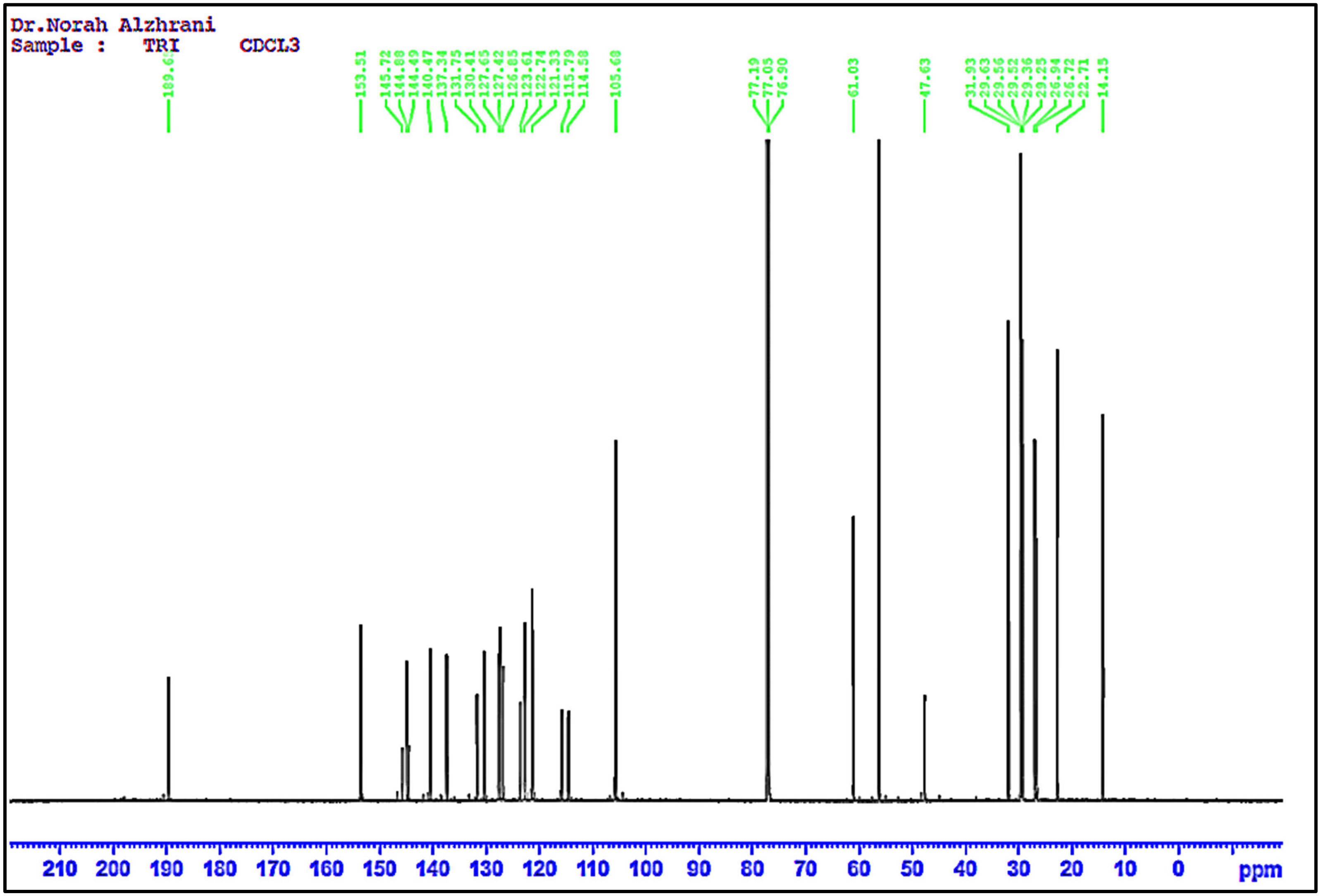

S58. ^{13}**C NMR of (E)-1-(*10-dodecylphenothiazin-2-yl*)-3-(3,4,5-trimethoxyphenyl)prop-2-en-1-on (4k).**

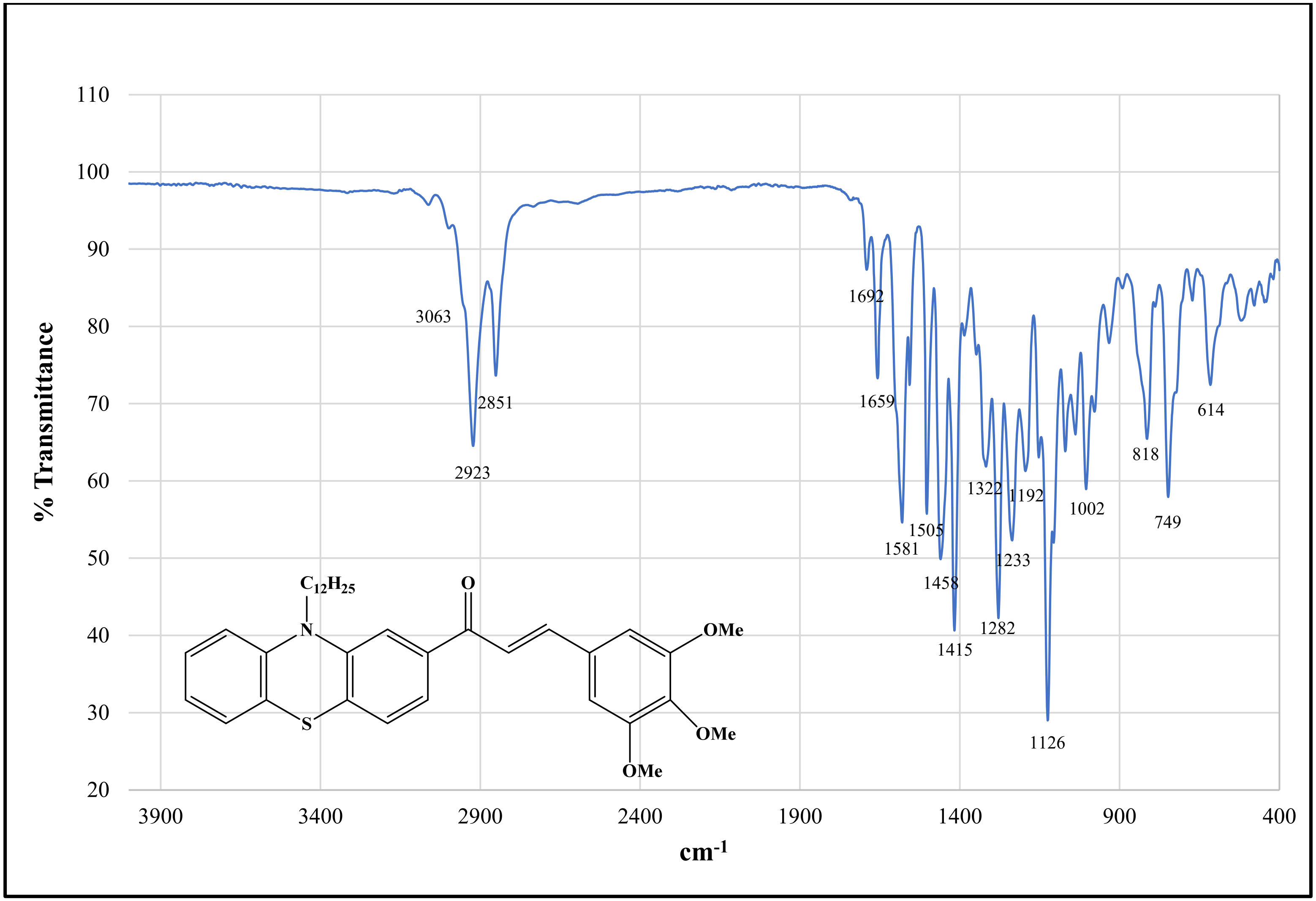

S59. IR of (E)-1-(*10-dodecylphenothiazin-2-yl*)-3-(3,4,5-trimethoxyphenyl)prop-2-en-1-on (4k).

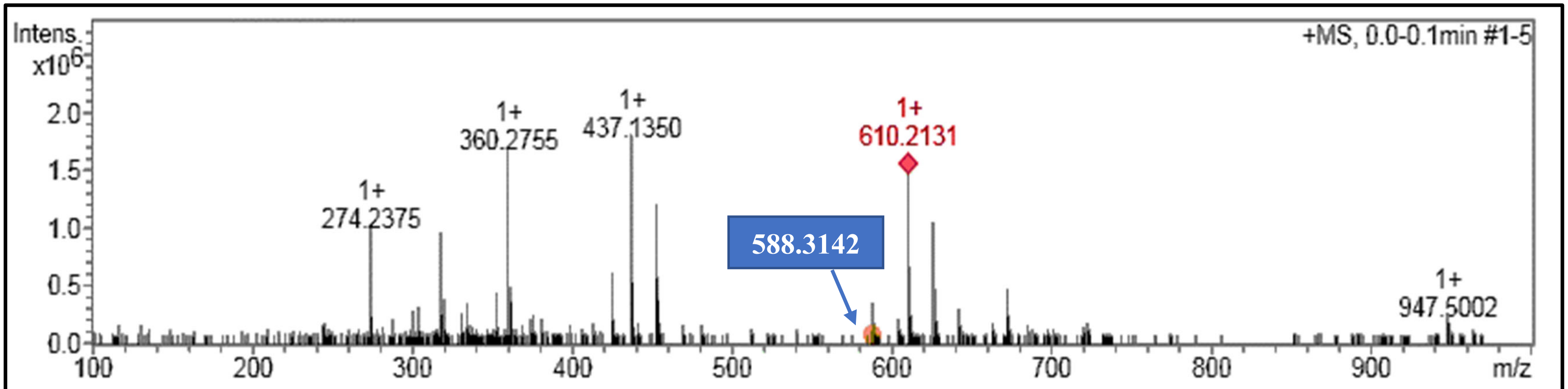

Meas. m/z	#	Ion Formula	m/z	err [ppm]	Mean err [ppm]	rdb	N-Rule	e⁻ Conf	mSigma	Std I	Std Mean m/z	Std I VarNorm	Std m/z Diff	Std Comb Dev
587.829883	1	C36H46NO4S	588.314206	823.2	849.5	14.5	ok	even	619.9	575.7	n.a.	n.a.	n.a.	n.a.

S60. MS of (E)-1-(*10-dodecylphenothiazin-2-yl*)-3-(3,4,5-trimethoxyphenyl)prop-2-en-1-on (4k).

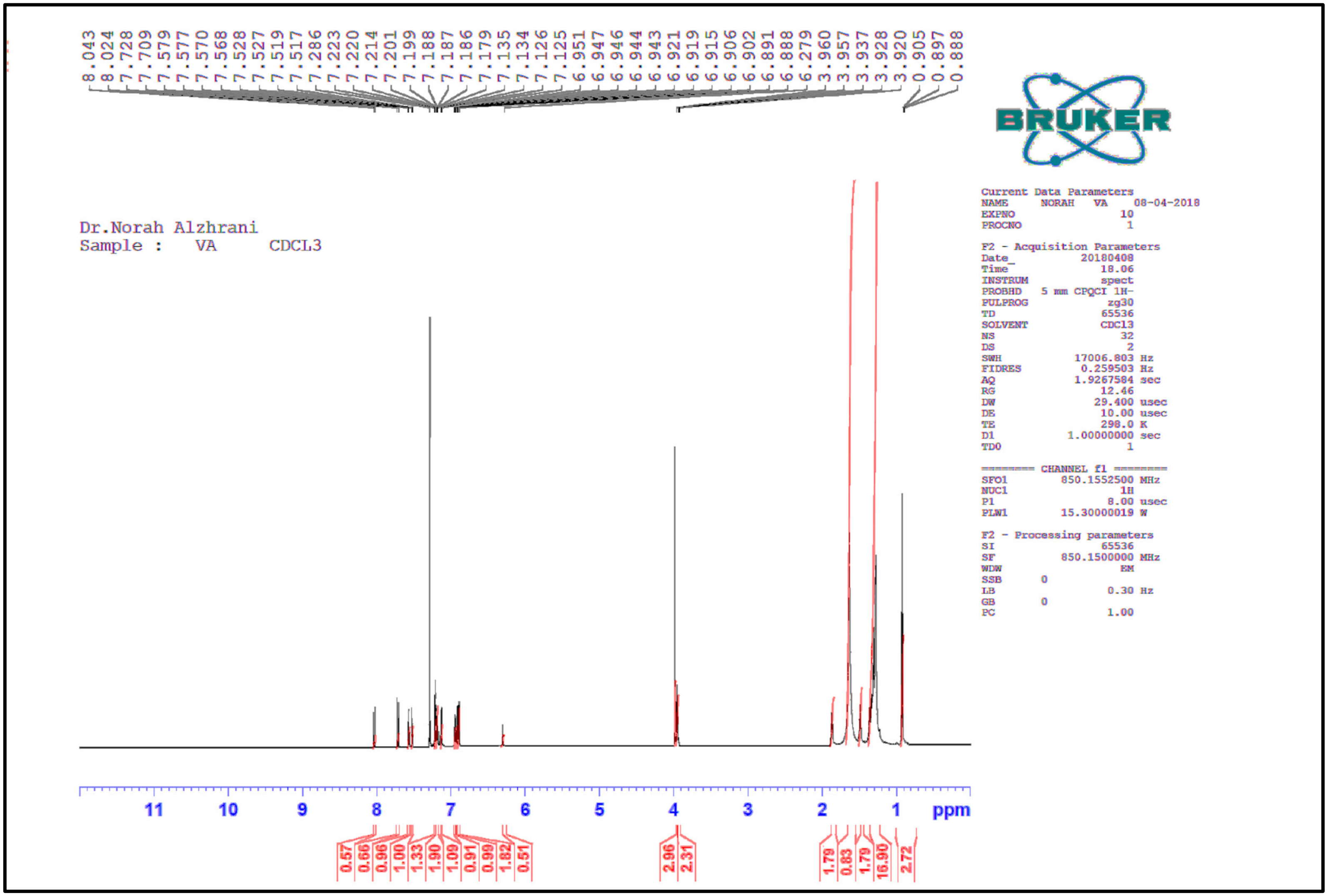

S61. ^{1}H NMR of (E)-1-(*10-dodecylphenothiazin-2-yl*)-3-(3-methoxyphenyl)prop-2-en-1-one (4l).

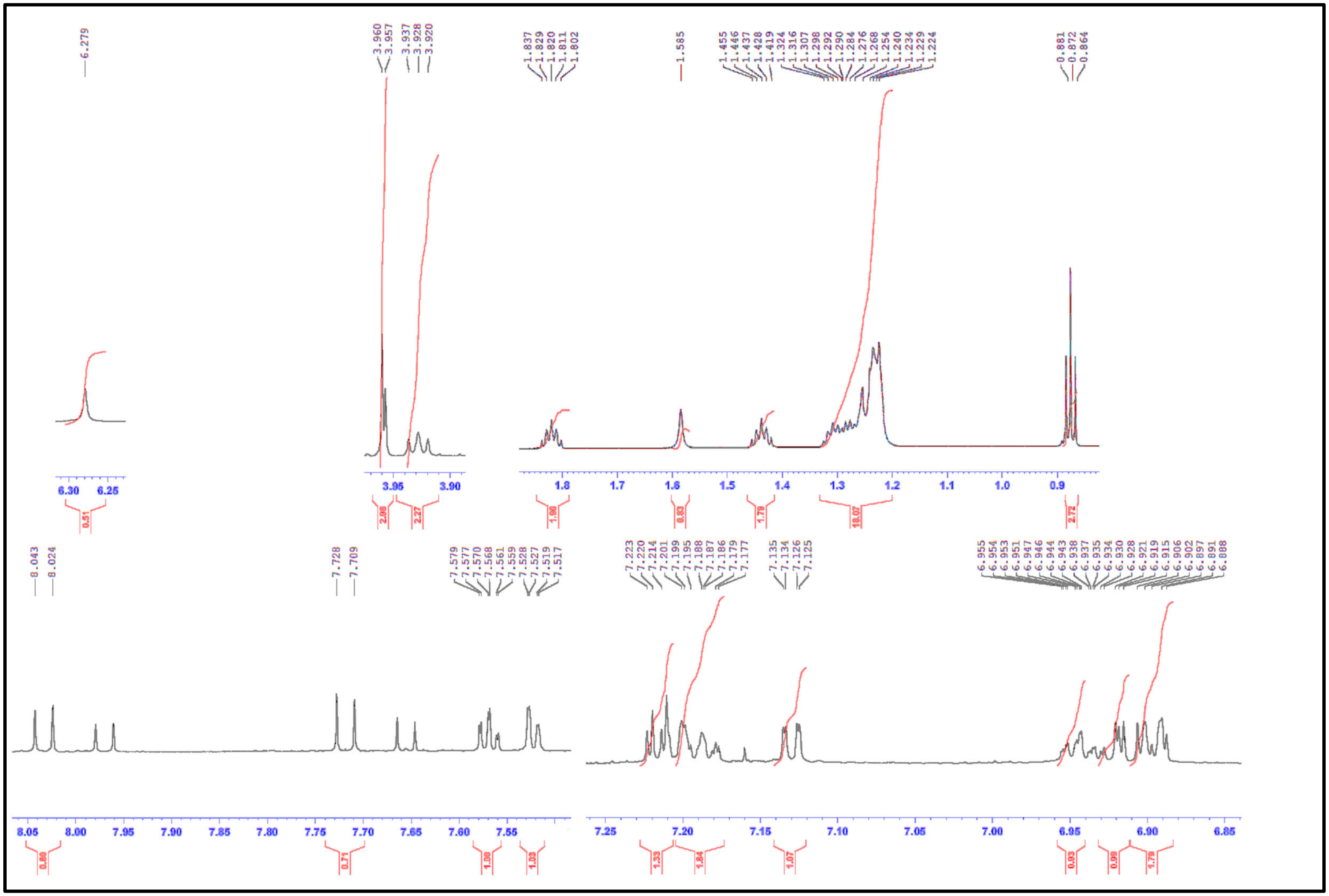

S62. ^{1}H NMR of (E)-1-(*10-dodecylphenothiazin-2-yl*)-3-(3-methoxyphenyl)prop-2-en-1-one (4l).

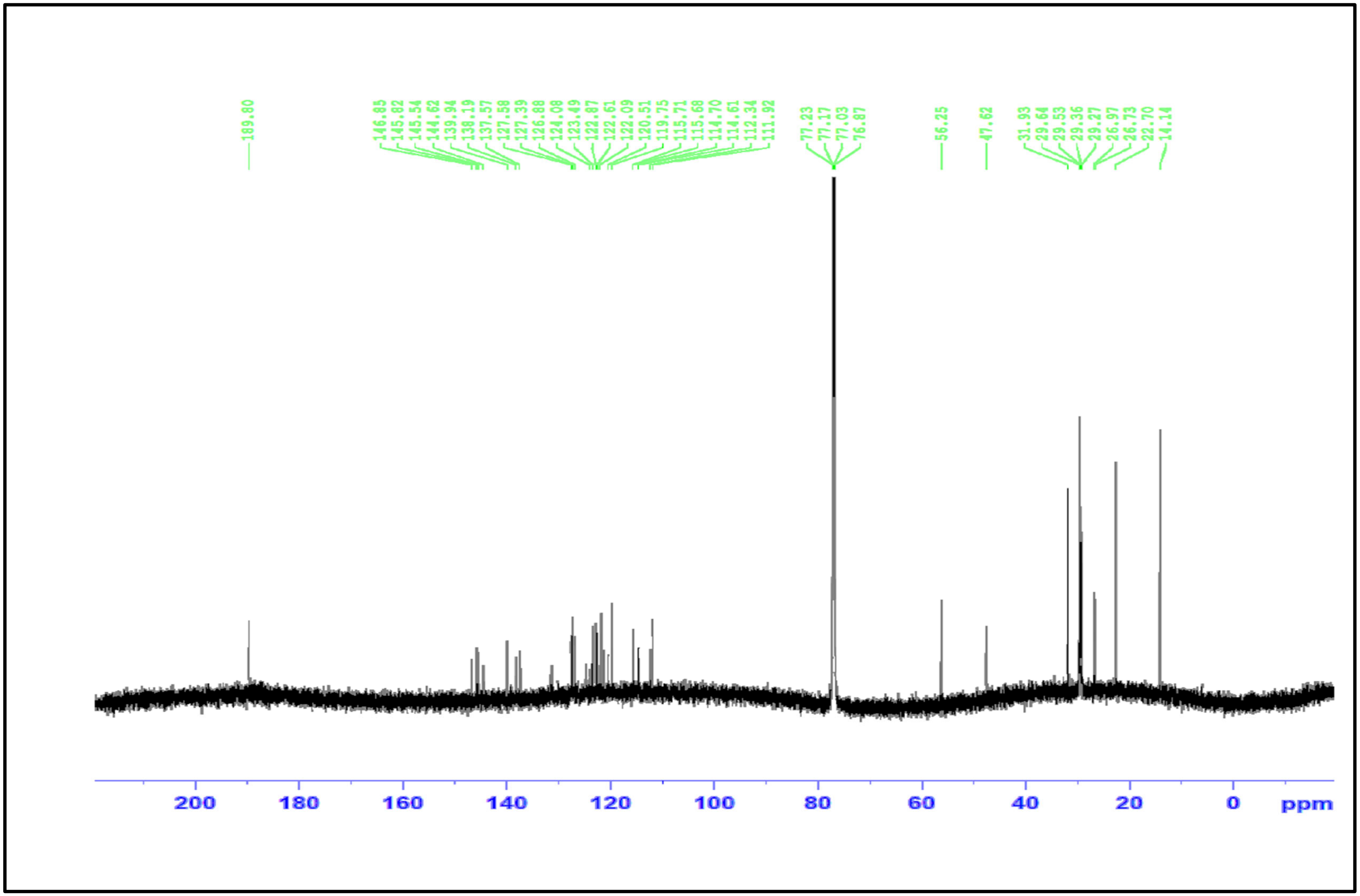

S63. ^{13}C NMR of (E)-1-(*10-dodecylphenothiazin-2-yl*)-3-(3-methoxyphenyl)prop-2-en-1-one (4l).

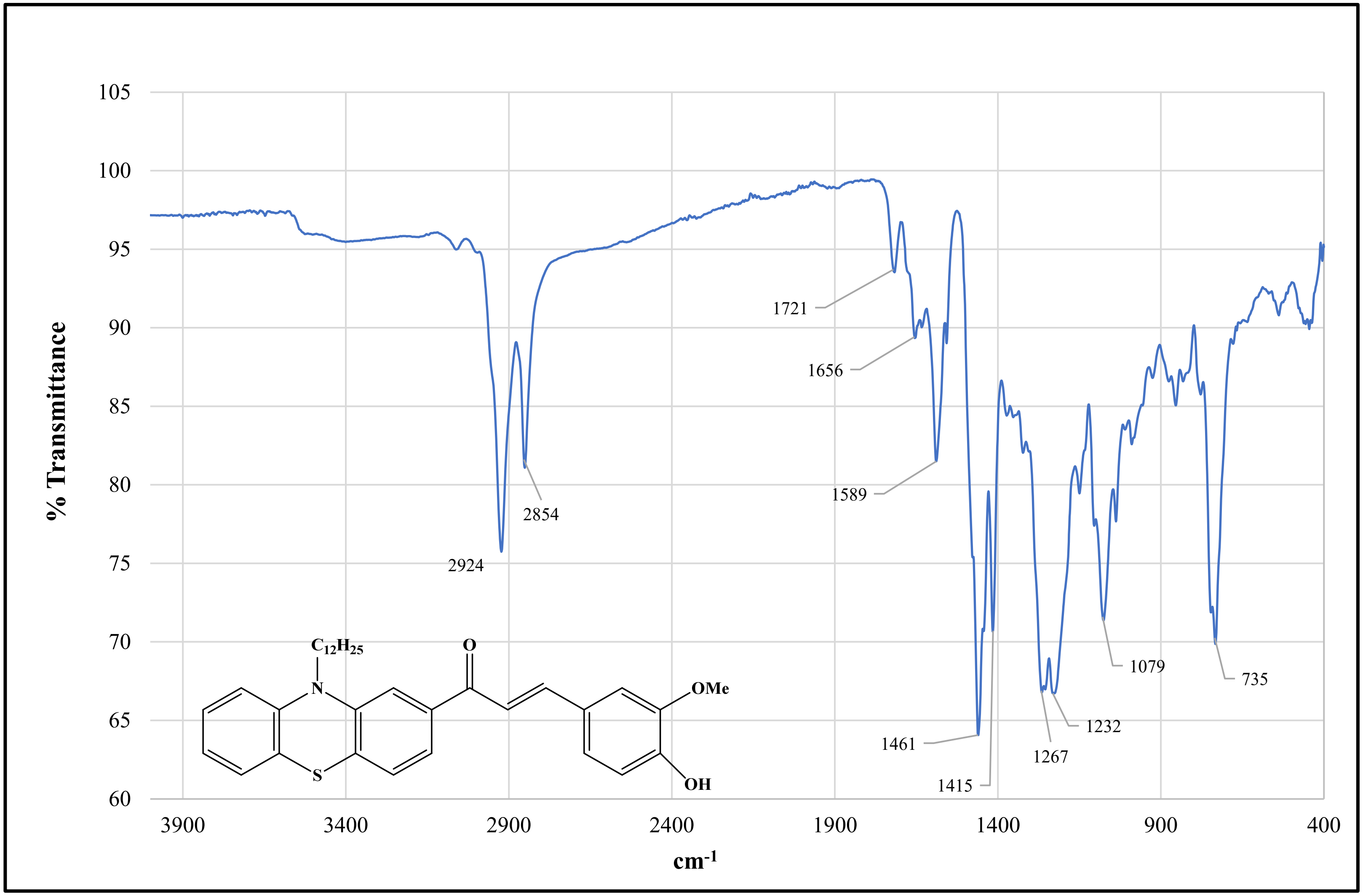

S64. IR of (E)-1-(*10-dodecylphenothiazin-2-yl*)-3-(3-methoxyphenyl)prop-2-en-1-one (4l).

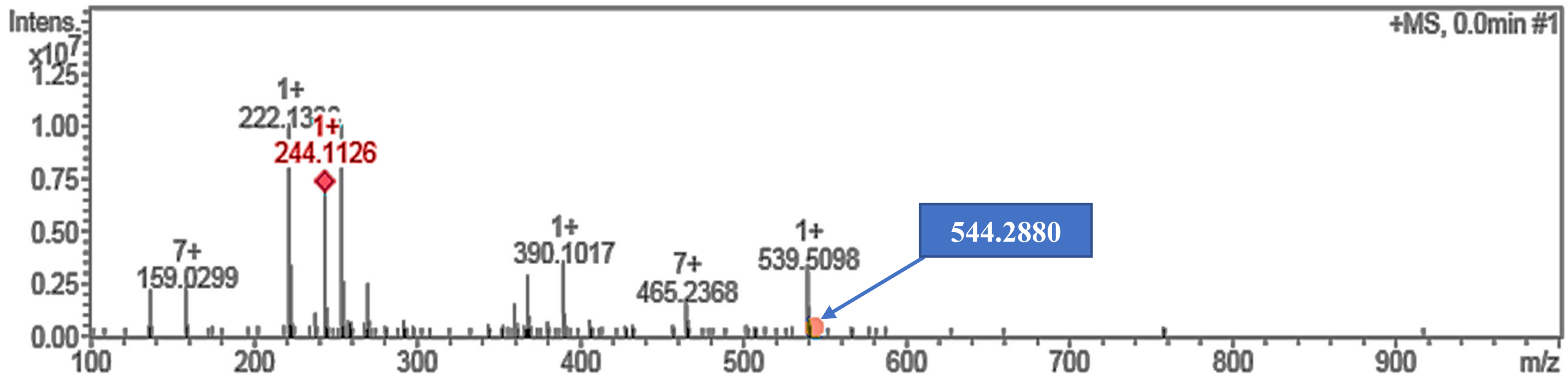

Meas. m/z	#	Ion Formula	m/z	err [ppm]	Mean err [ppm]	rdb	N-Rule	e⁻ Conf	mSigma	Std I	Std Mean m/z	Std I VarNorm	Std m/z Diff	Std Comb Dev
543.780272	1	C34H42NO3S	544.287992	932.8	944.3	14.5	ok	even	361.4	343.8	n.a.	n.a.	n.a.	n.a.

S65. MS of (E)-1-(*10-dodecylphenothiazin-2-yl*)-3-(3-methoxyphenyl)prop-2-en-1-one (4l).

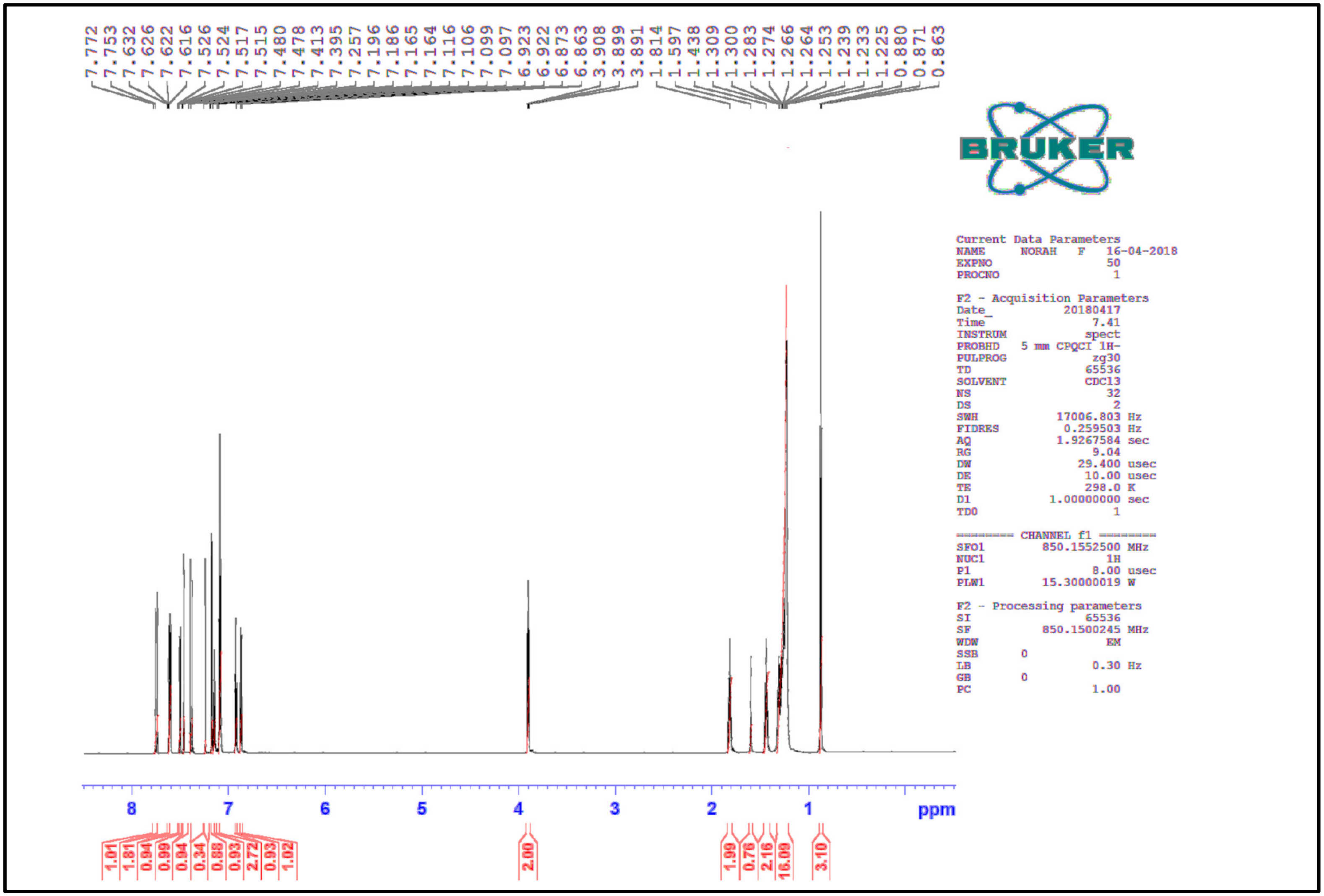

S66. ^{1}H NMR of (E)-1-(*10-dodecylphenothiazin-2-yl*)-3-(4-fluorophenyl)prop-2-en-1-on (4m).

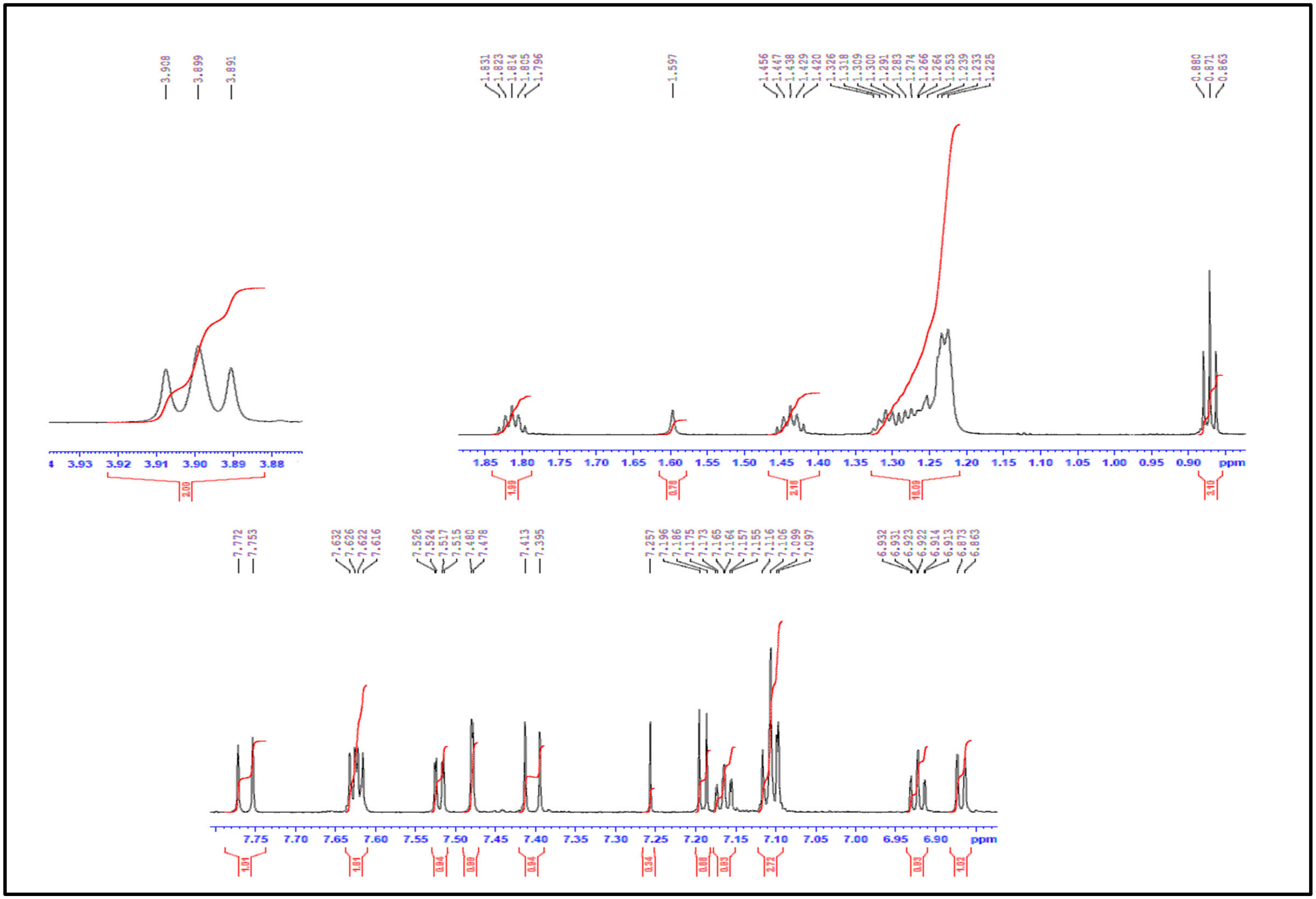

S67. ^{1}H NMR of (E)-1-(*10-dodecylphenothiazin-2-yl*)-3-(4-fluorophenyl)prop-2-en-1-on (4m).

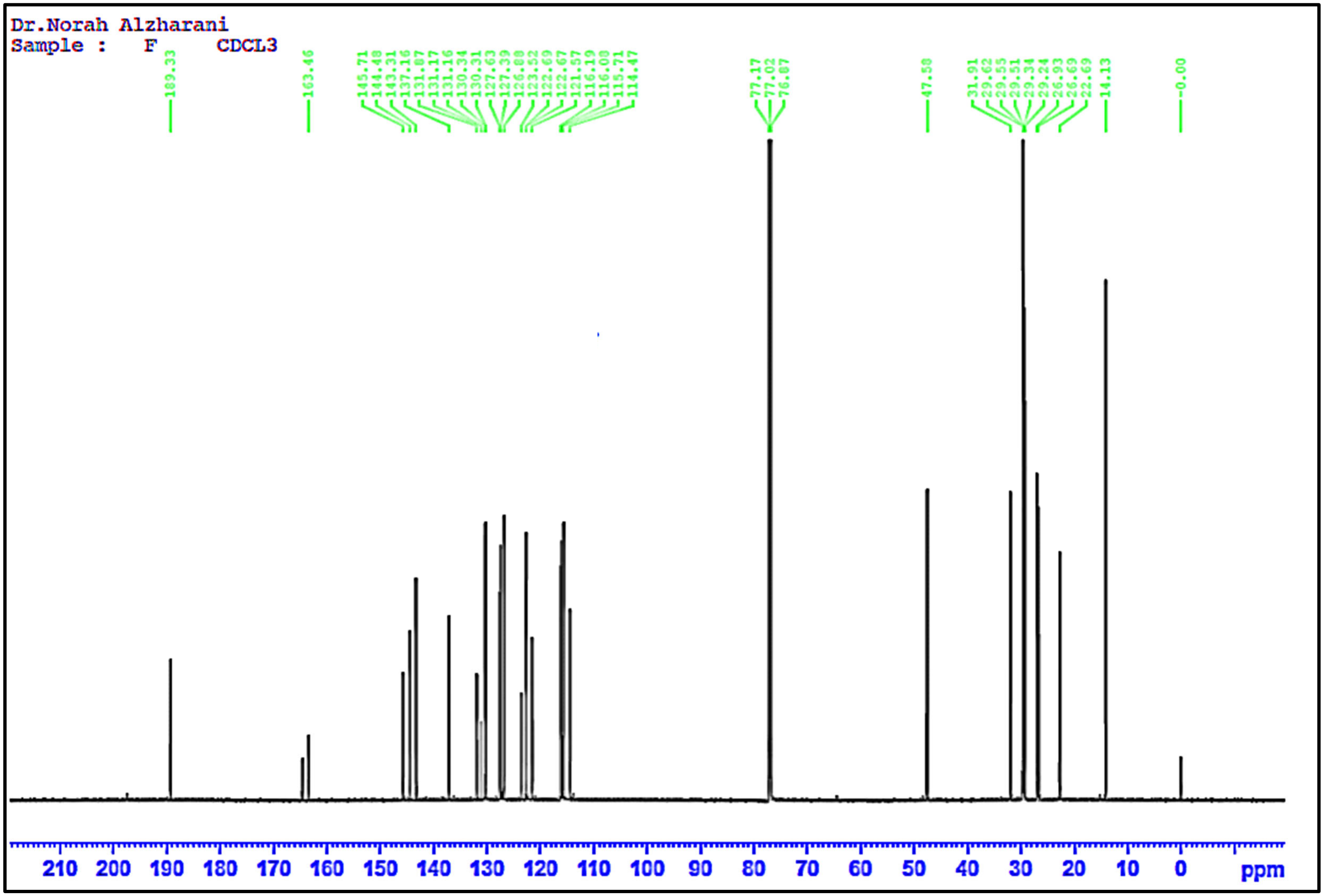

S68. ^{13}C NMR of (E)-1-(*10-dodecylphenothiazin-2-yl*)-3-(4-fluorophenyl)prop-2-en-1-on (4m).

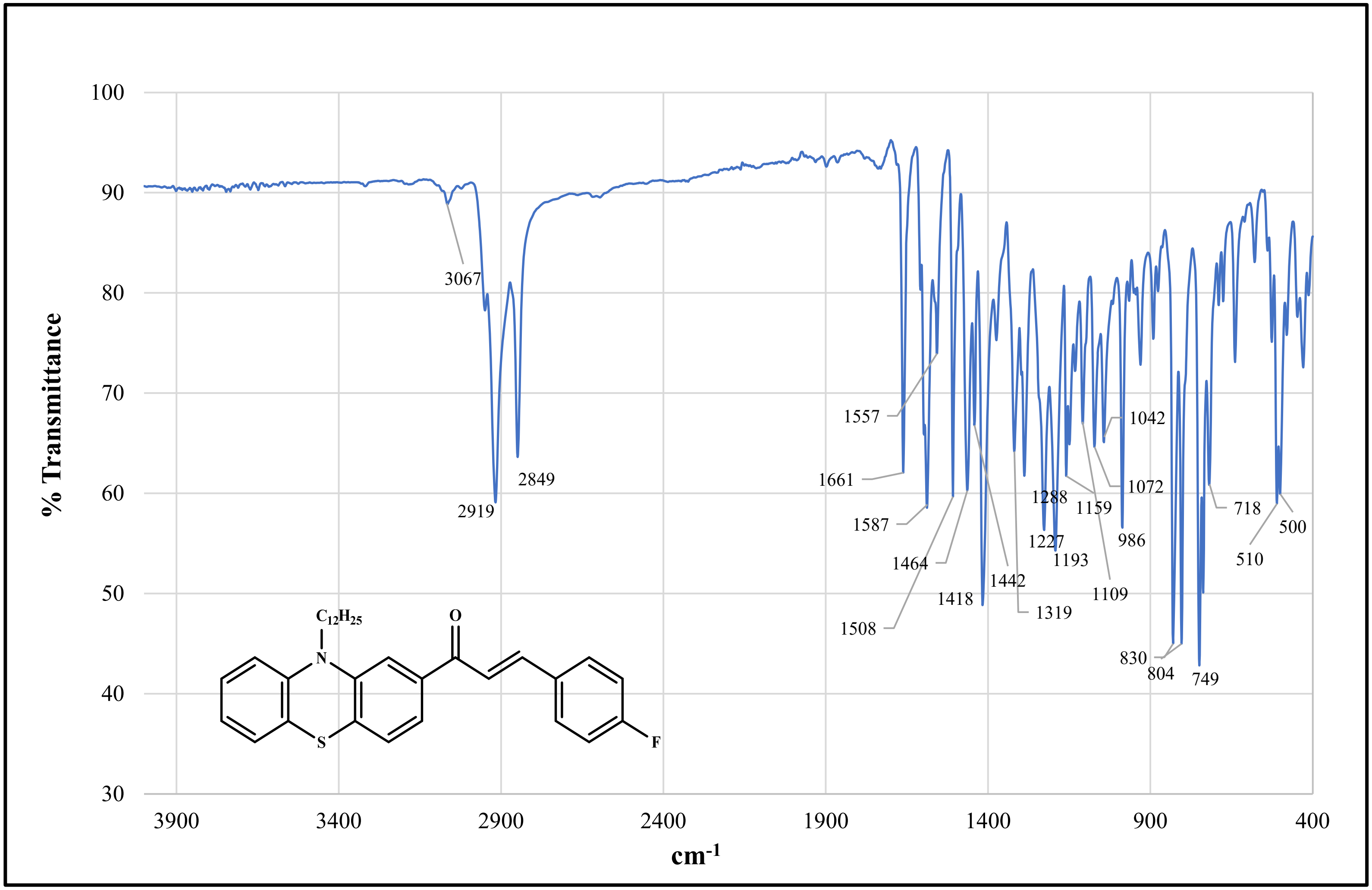

S69. IR of (E)-1-(*10-dodecylphenothiazin-2-yl*)-3-(4-fluorophenyl)prop-2-en-1-on (4m).

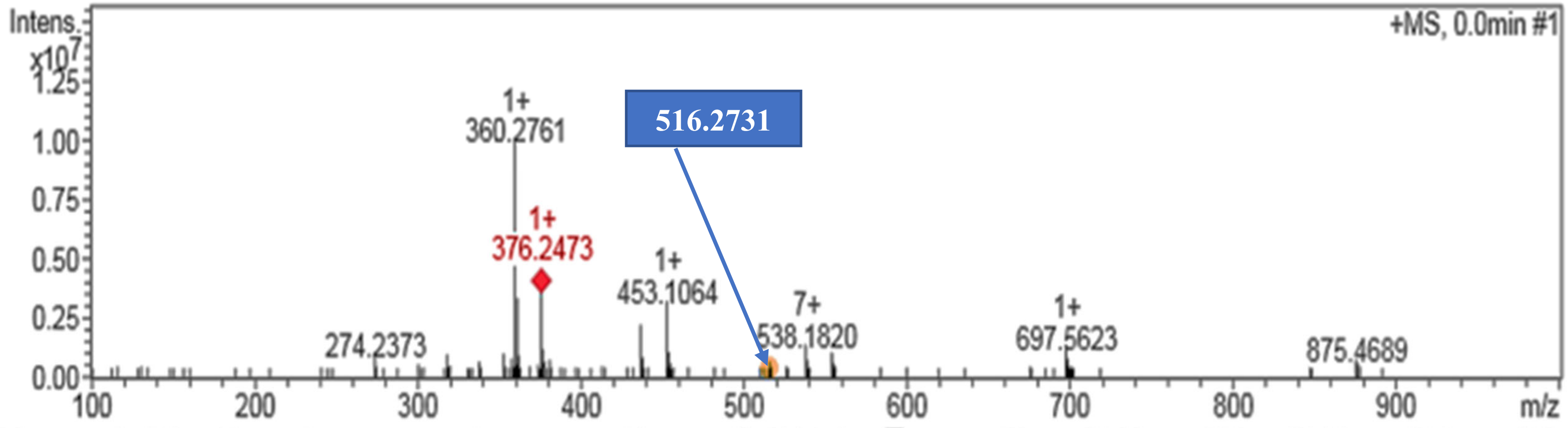

Meas. m/z	#	Ion Formula	m/z	err [ppm]	Mean err [ppm]	rdb	N-Rule	e⁻ Conf	mSigma	Std I	Std Mean m/z	Std I VarNorm	Std m/z Diff	Std Comb Dev
515.724548	1	C33H39FNOS	516.273091	1062.5	1065.5	14.5	ok	even	667.5	550.3	n.a.	n.a.	n.a.	n.a.

S70. MS of (E)-1-(*10-dodecylphenothiazin-2-yl*)-3-(4-fluorophenyl)prop-2-en-1-on (4m).

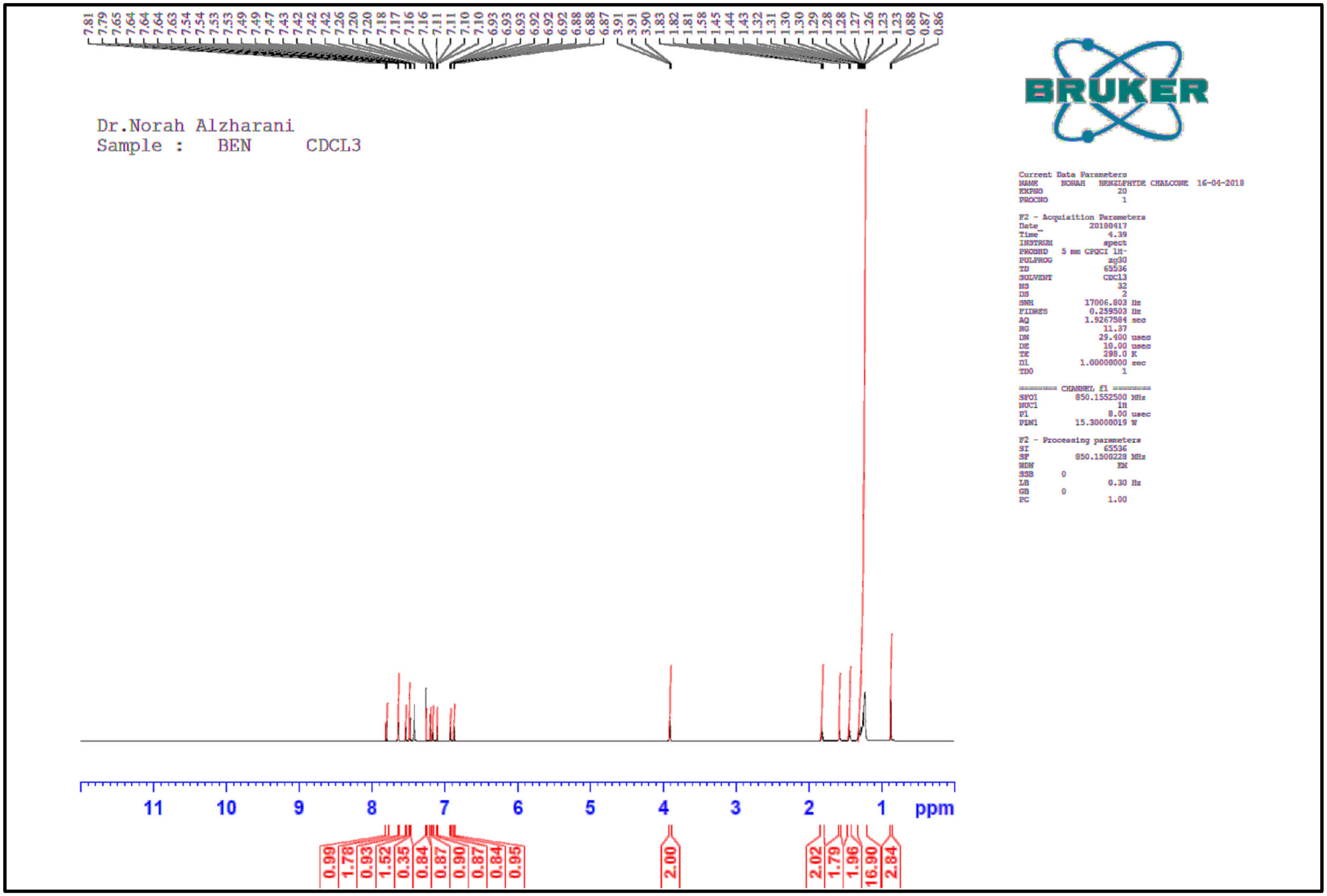

S71. **^{1}H NMR of (E)-1-(*10-dodecylphenothiazin-2-yl*)-3-phenylprop-2-en-1-one (4n).**

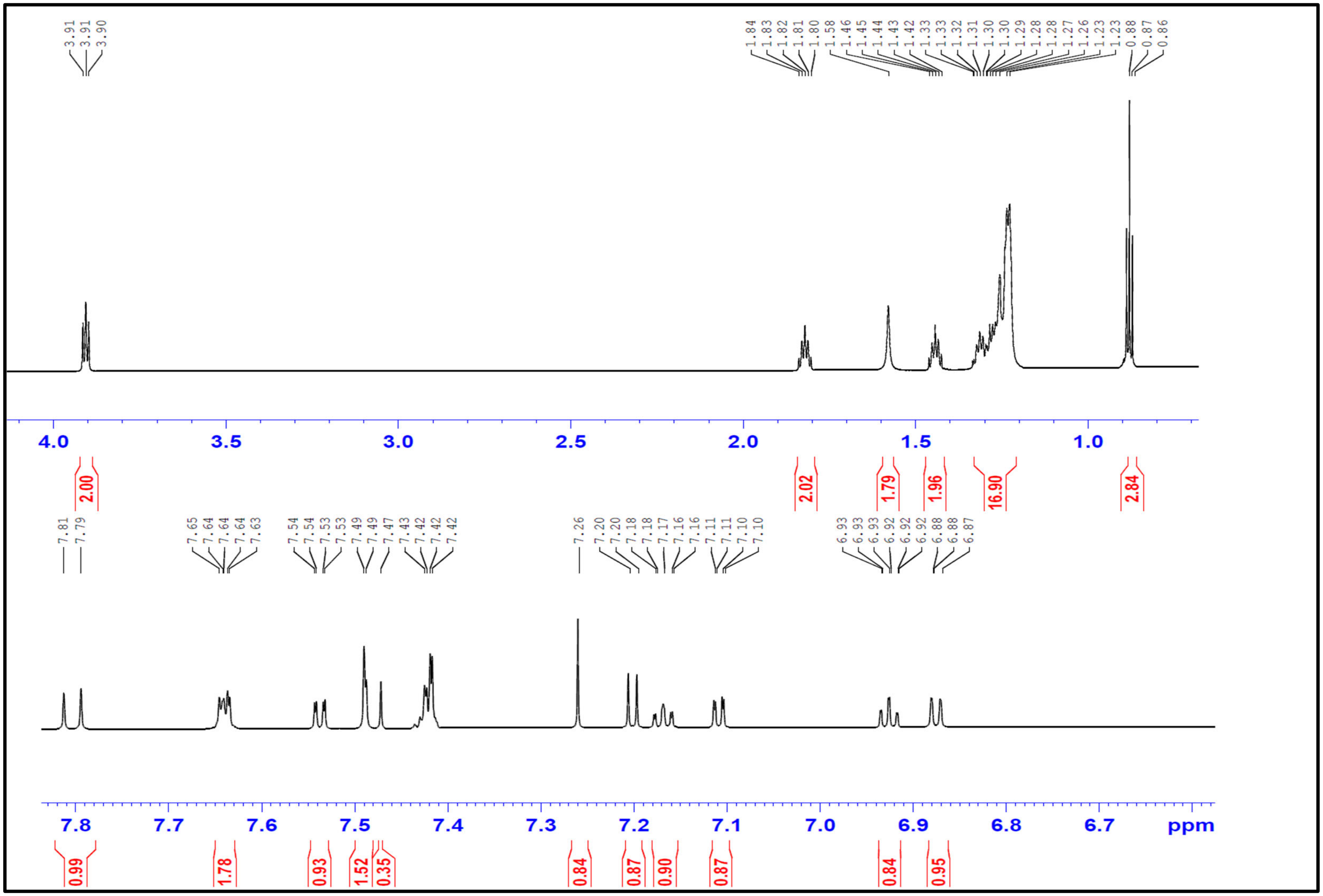

S72. ^{1}H NMR of (E)-1-(*10-dodecylphenothiazin-2-yl*)-3-phenylprop-2-en-1-one (4n).

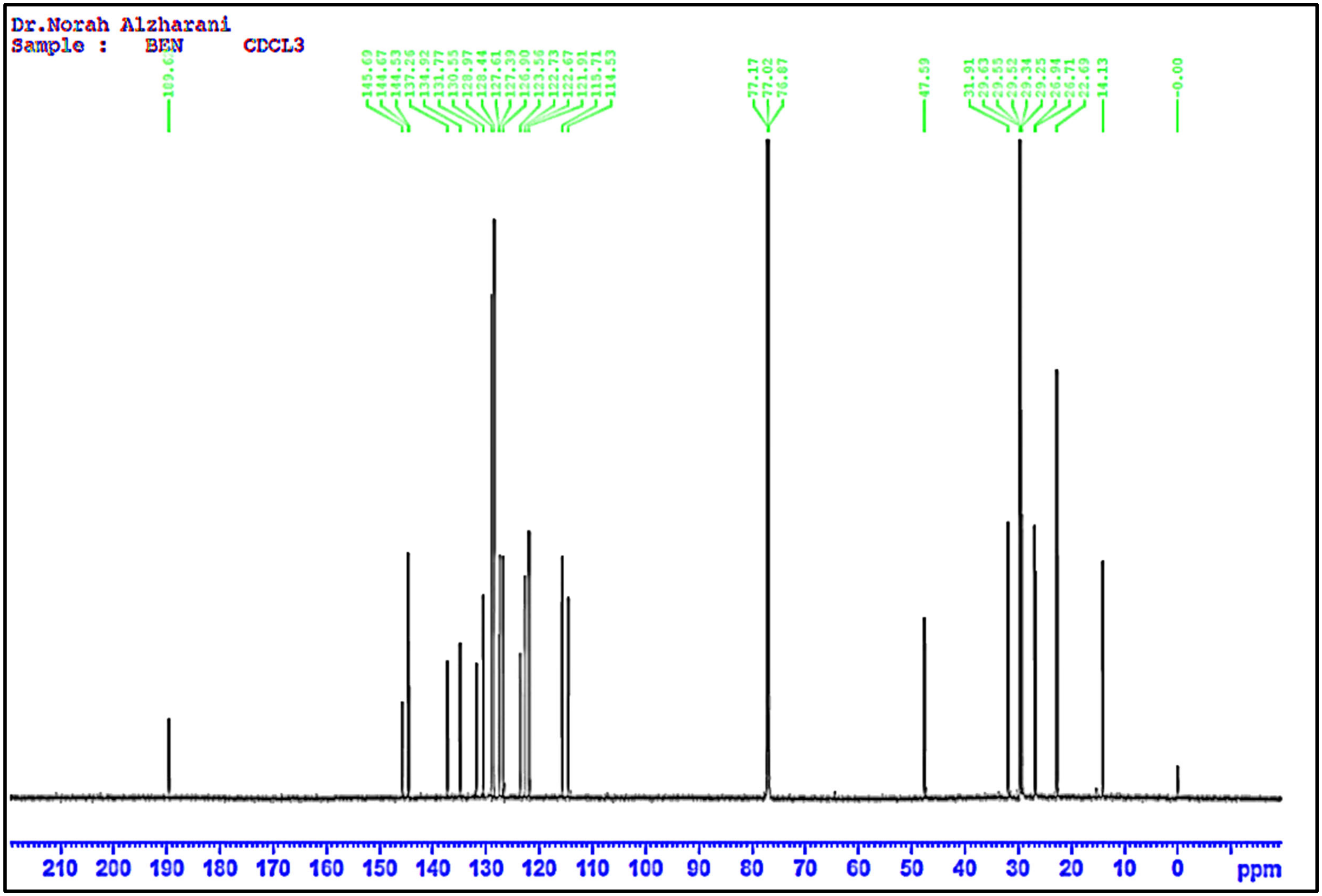

S73. ^{13}C NMR of (E)-1-(*10-dodecylphenothiazin-2-yl*)-3-phenylprop-2-en-1-one (4n).

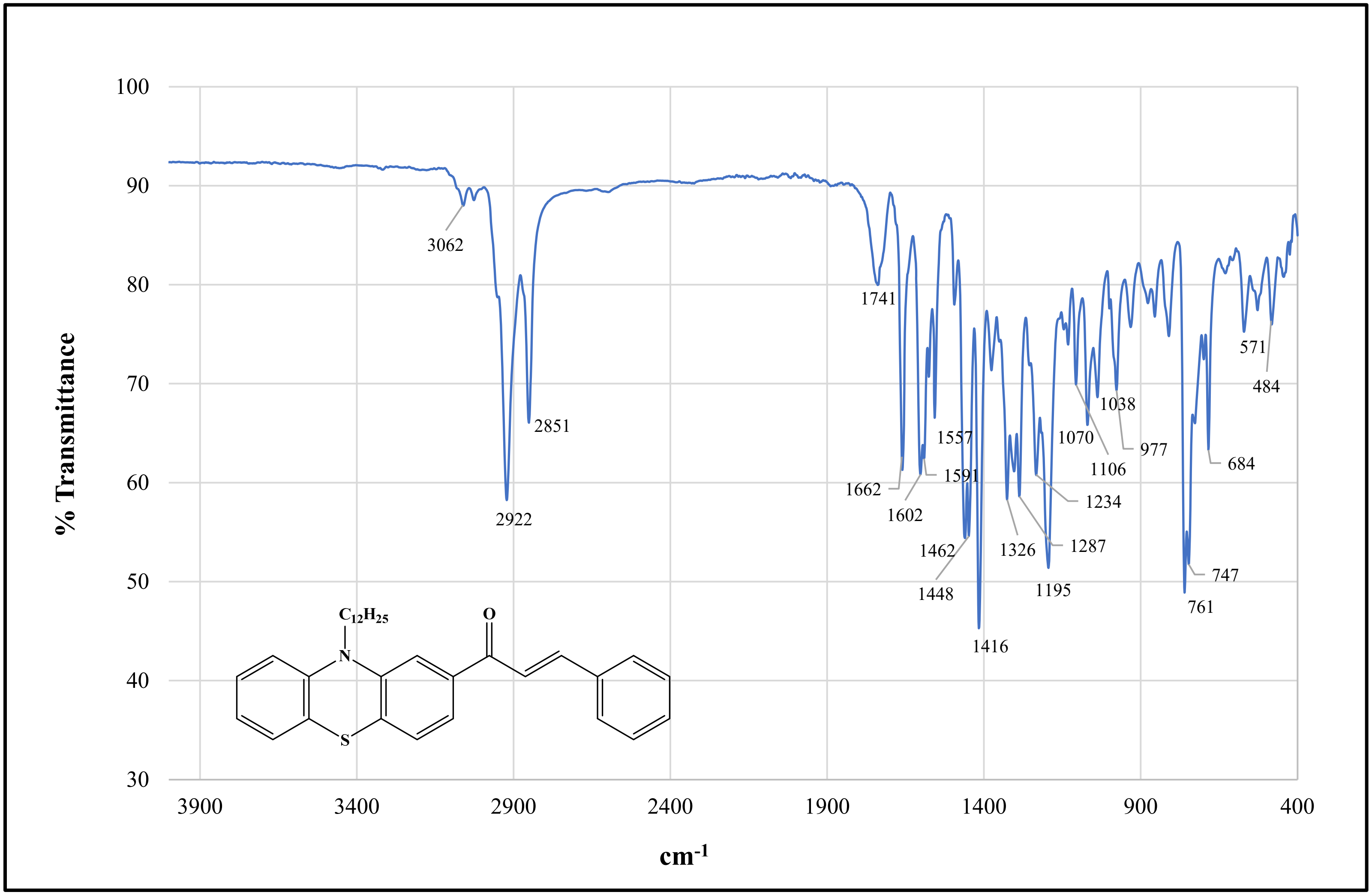

S74. IR of (E)-1-(*10-dodecylphenothiazin-2-yl*)-3-phenylprop-2-en-1-one (4n).

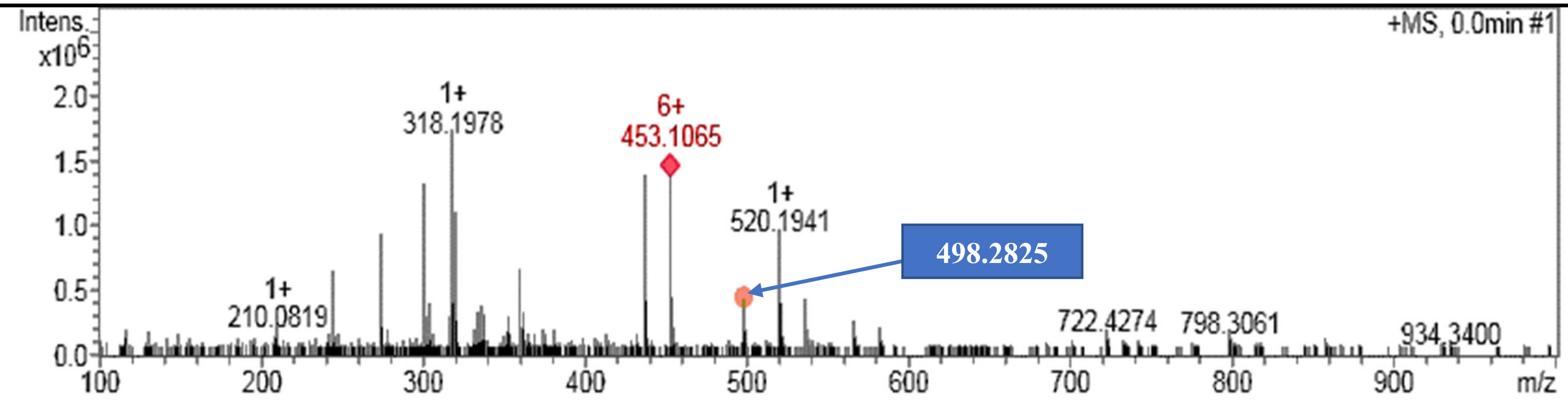

Meas. m/z	#	Ion Formula	m/z	err [ppm]	Mean err [ppm]	rdb	N-Rule	e⁻ Conf	mSigma	Std I	Std Mean m/z	Std I VarNorm	Std m/z Diff	Std Comb Dev
498.215128	1	C33H40NOS	498.282512	135.2	135.5	14.5	ok	even	9.3	15.5	n.a.	n.a.	n.a.	n.a.

S75. MS of (E)-1-(*10-dodecylphenothiazin-2-yl*)-3-phenylprop-2-en-1-one (4n).

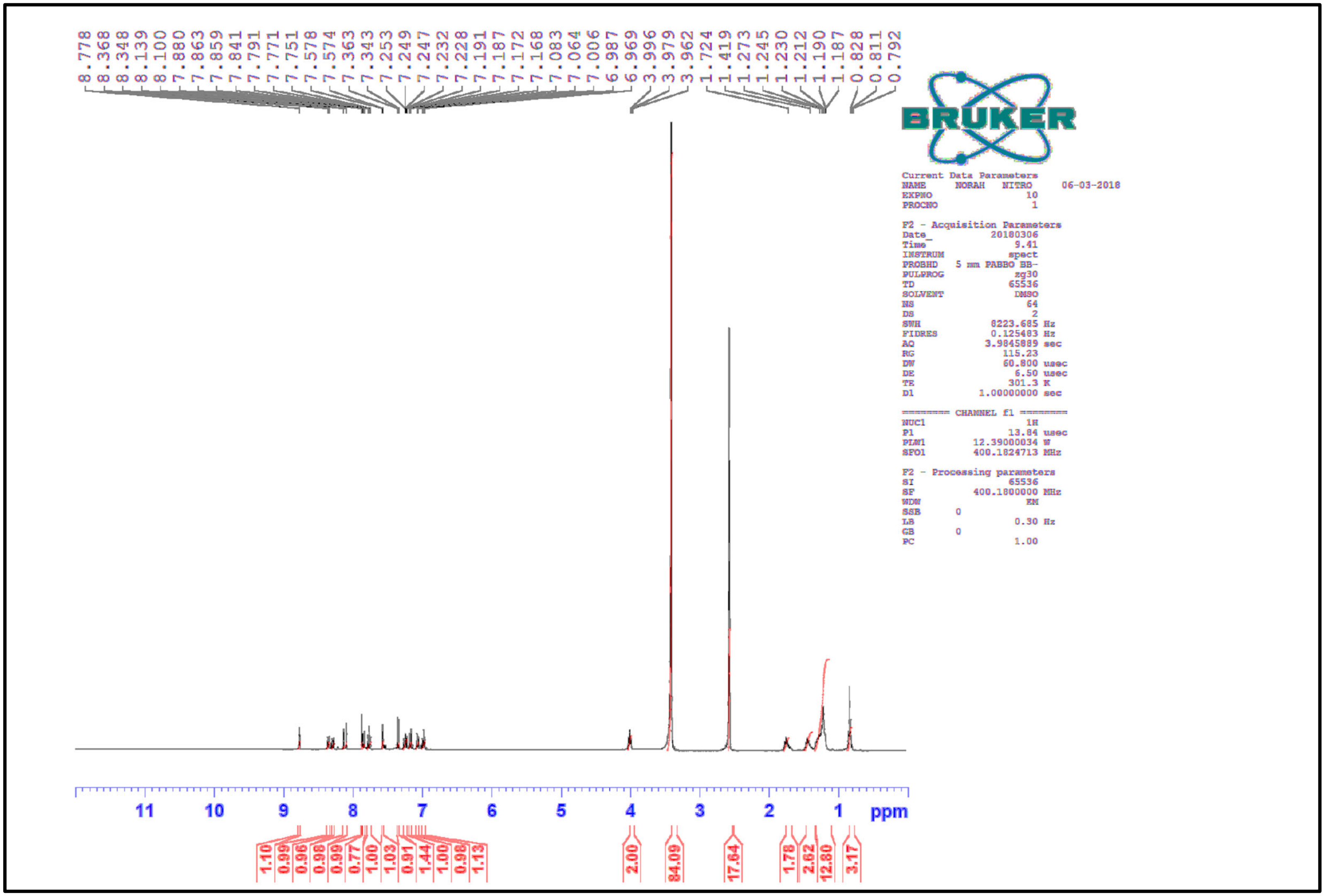

S76. ^{1}H NMR of (E)-1-(*10-dodecylphenothiazin-2-yl*)-3-(3-nitrophenyl)prop-2-en-1-one (4o).

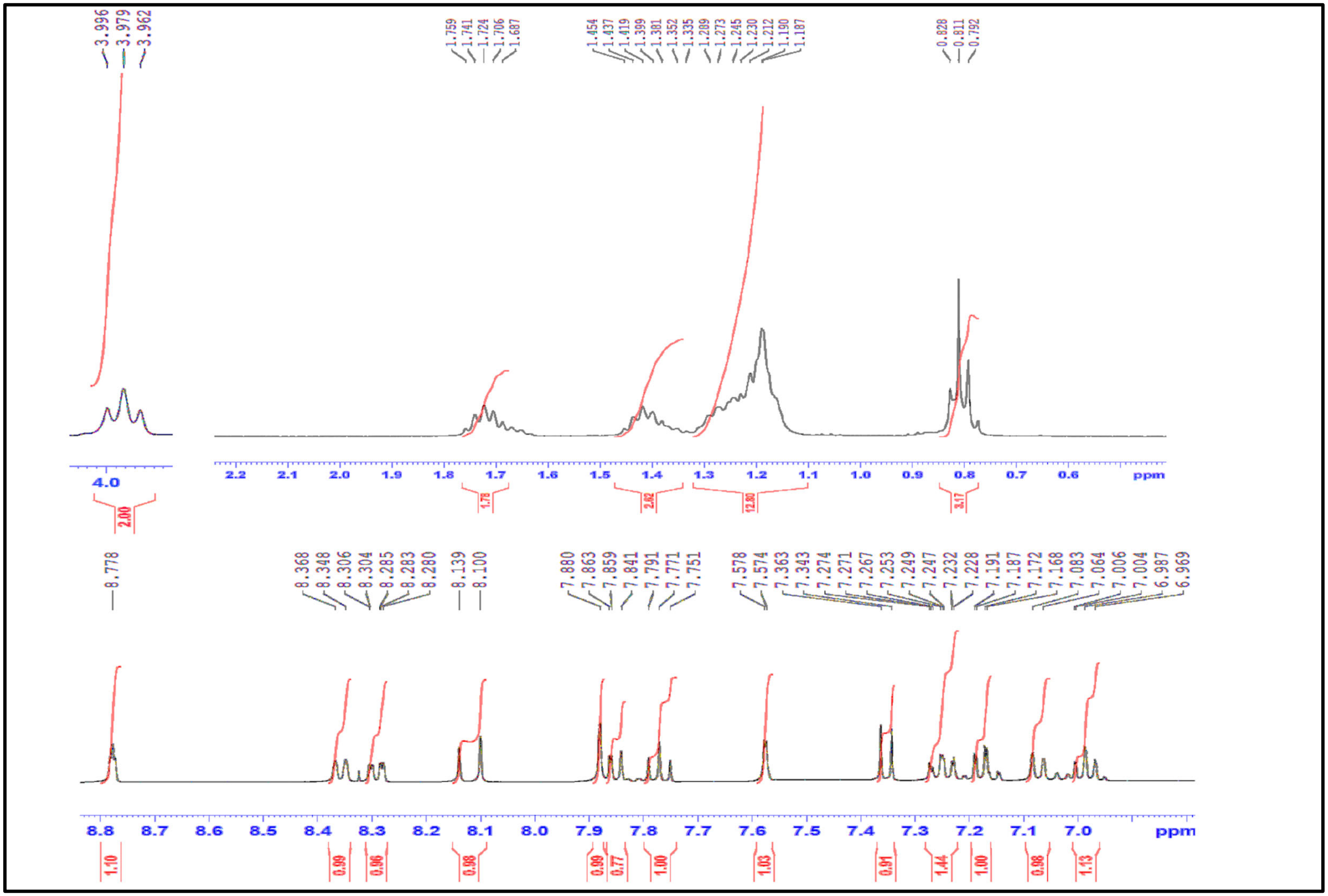

S77. ^{1}H NMR of (E)-1-(*10-dodecylphenothiazin-2-yl*)-3-(3-nitrophenyl)prop-2-en-1-one (4o).

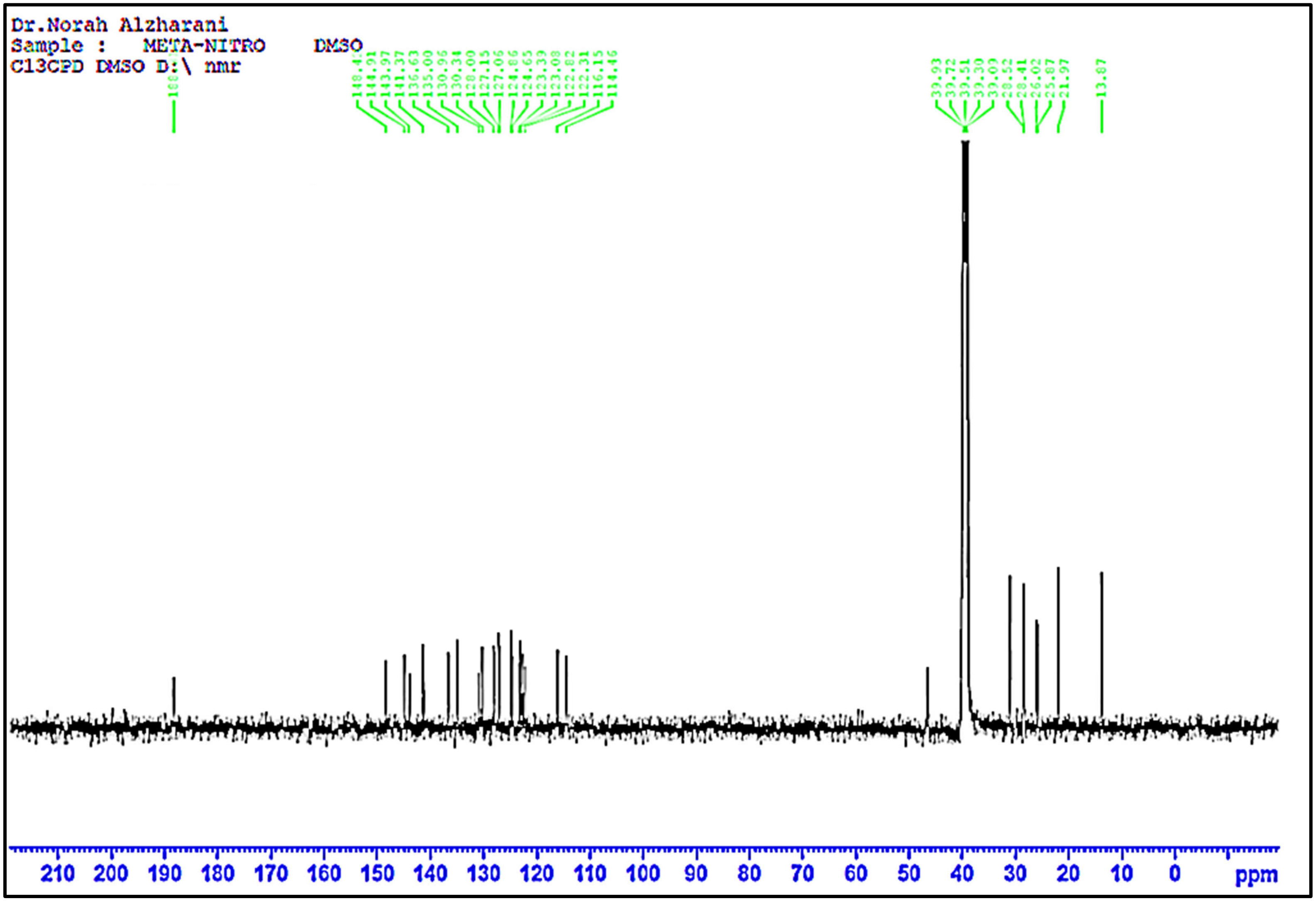

S78. ^{13}C NMR of (E)-1-(*10-dodecylphenothiazin-2-yl* 3-(3-nitrophenyl)prop-2-en-1-one (4o).

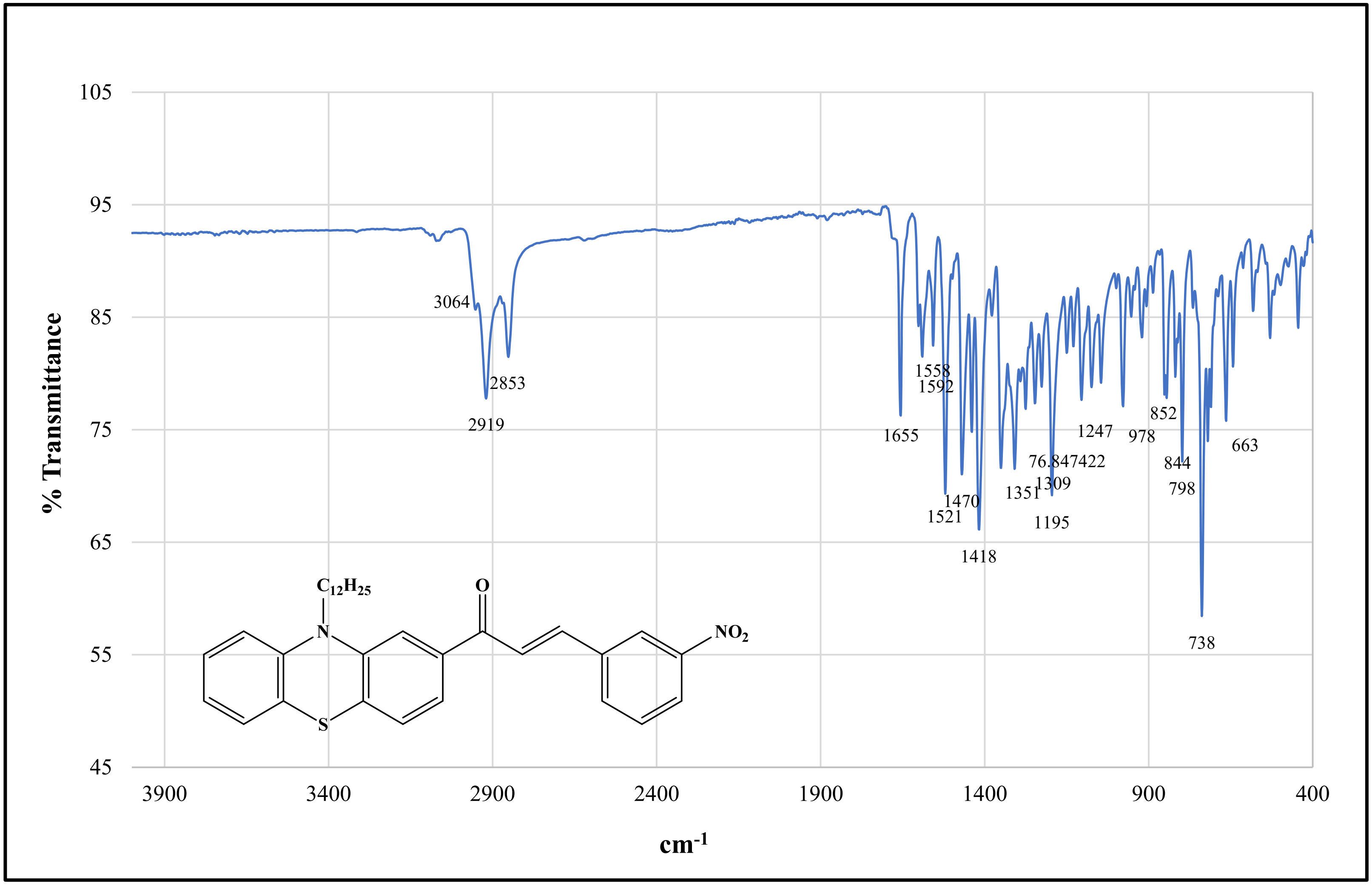

S79. IR of (E)-1-(*10-dodecylphenothiazin-2-yl*)-3-(3-nitrophenyl)prop-2-en-1-one (4o).

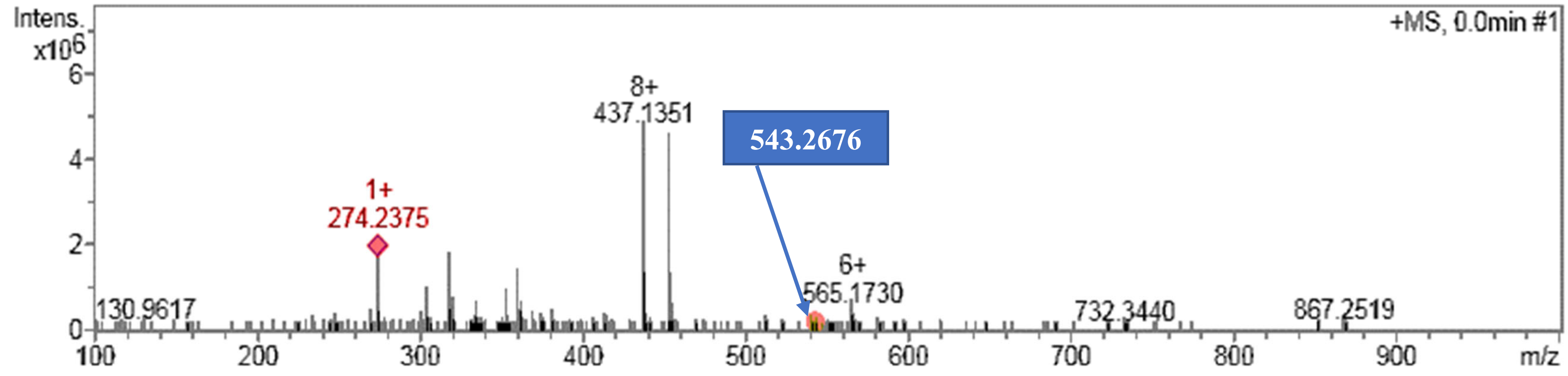

Meas. m/z	#	Ion Formula	m/z	err [ppm]	Mean err [ppm]	rdb	N-Rule	e⁻ Conf	mSigma	Std I	Std Mean m/z	Std I VarNorm	Std m/z Diff	Std Comb Dev
542.747265	1	C33H39N2O3S	543.267591	957.8	976.5	15.5	ok	even	635.6	530.3	n.a.	n.a.	n.a.	n.a.

S80. MS of (E)-1-(*10-dodecylphenothiazin-2-yl*)-3-(3-nitrophenyl)prop-2-en-1-one (4o).

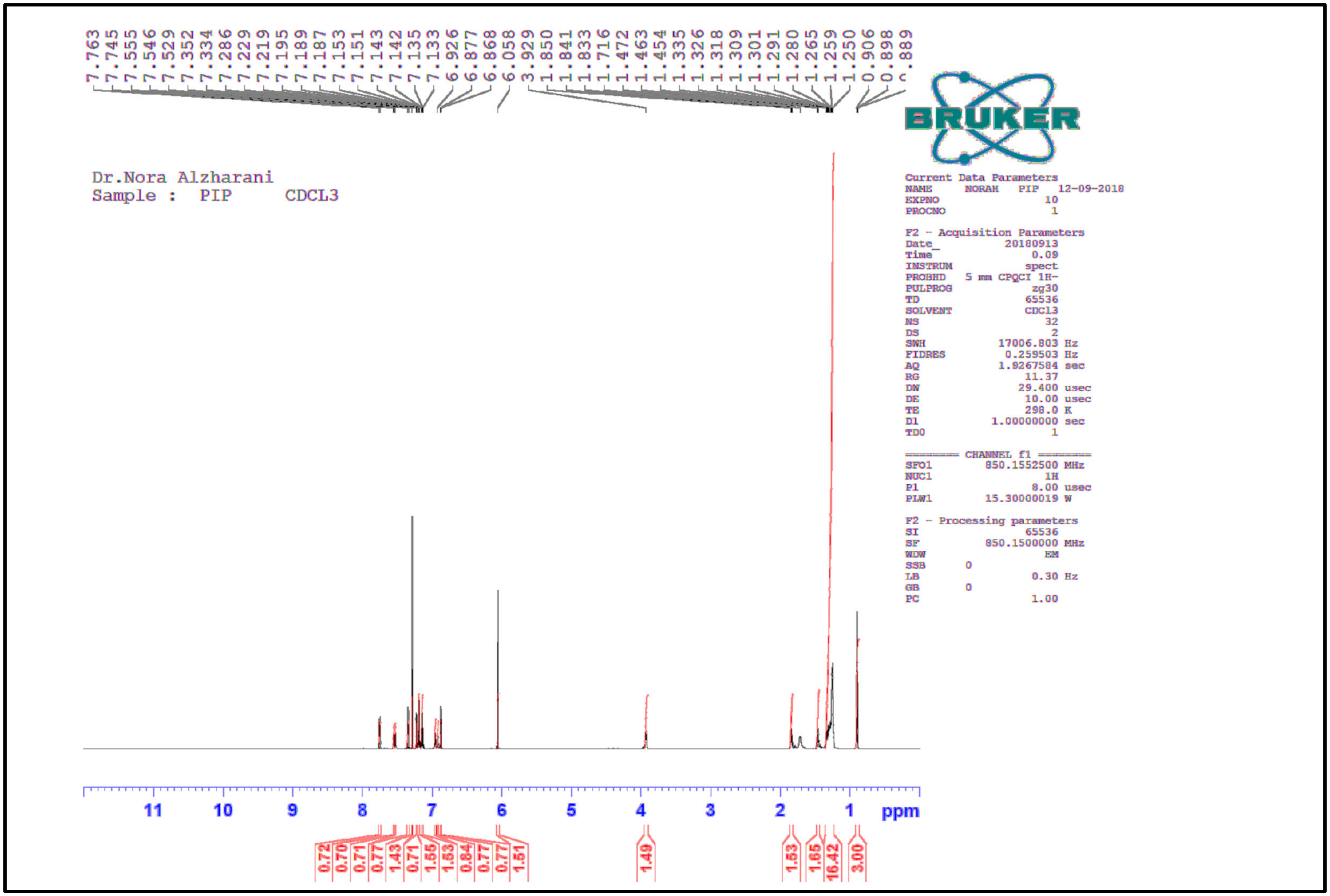

S81. ^{1}H NMR of (E)-3-(benzo[d][1,3]dioxol-5-yl)-1-(*10-dodecylphenothiazin-2-yl*)prop-2-en-1-one (4p).

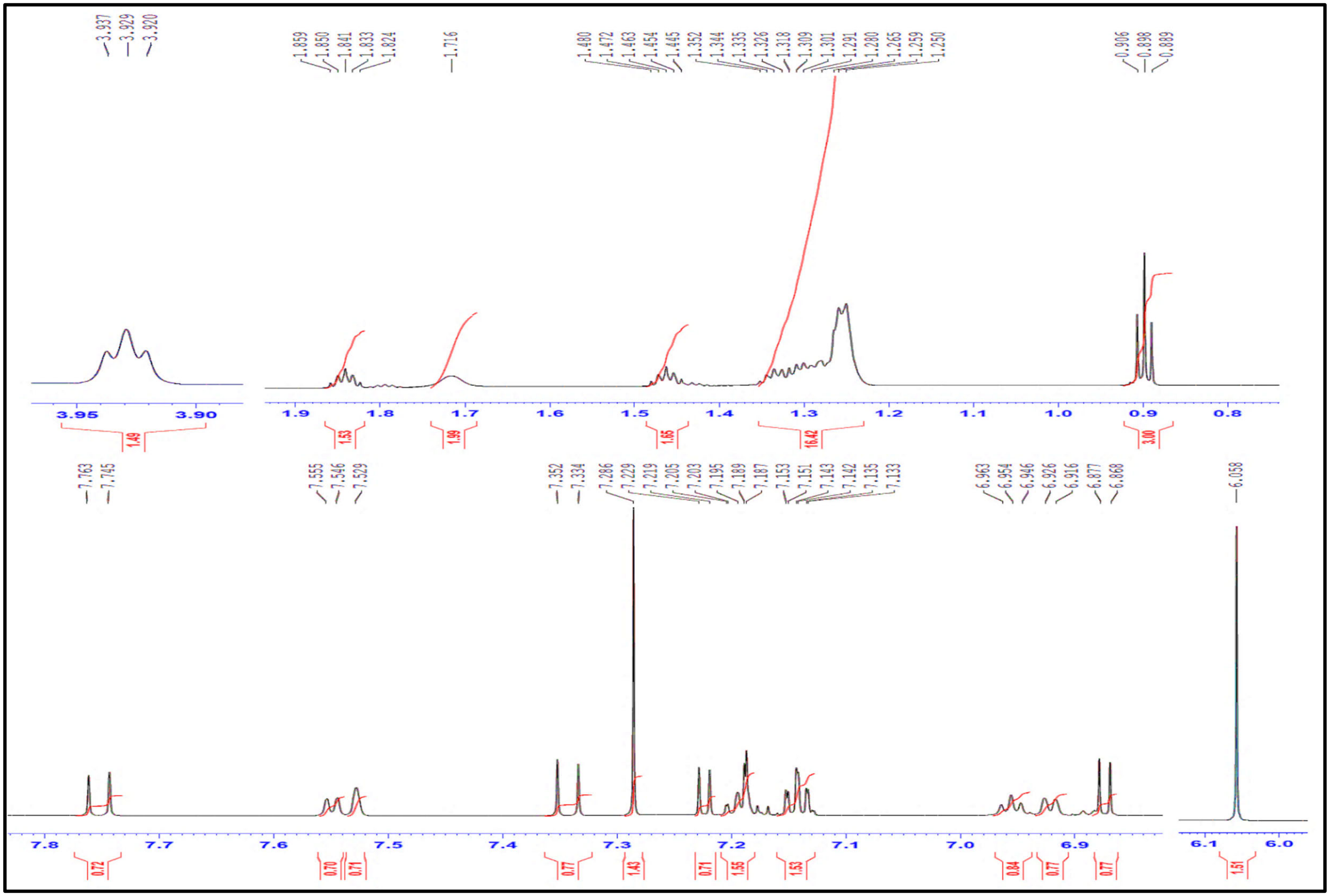

S82. **^{1}H NMR of (E)-3-(benzo[d][1,3]dioxol-5-yl)-1-(*10-dodecylphenothiazin-2-yl*)prop-2-en-1-one (4p).**

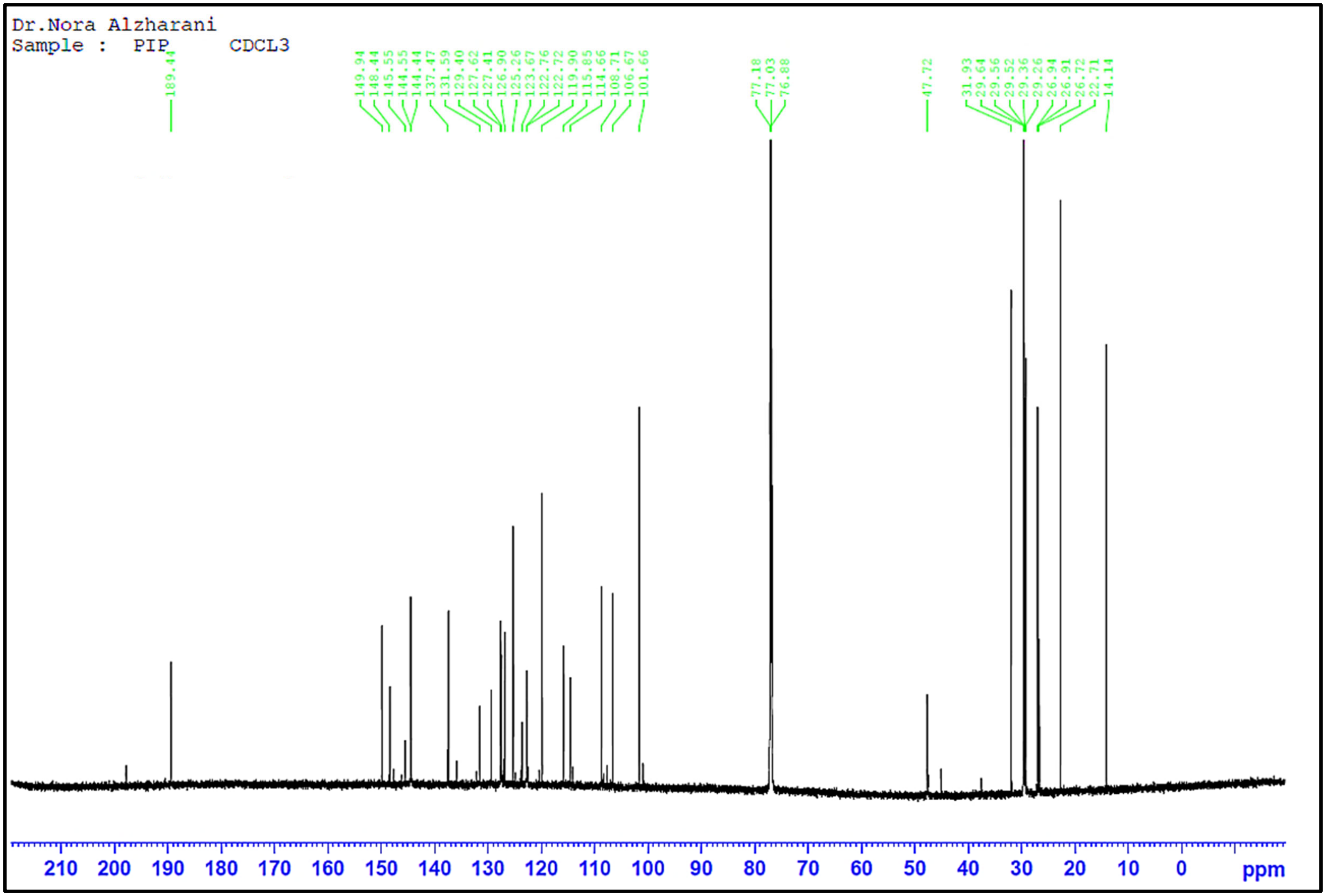

S83. ^{13}**C NMR of (E)-3-(benzo[d][1,3]dioxol-5-yl)-1-(***10-dodecylphenothiazin-2-yl***)prop-2-en-1-one (4p).**

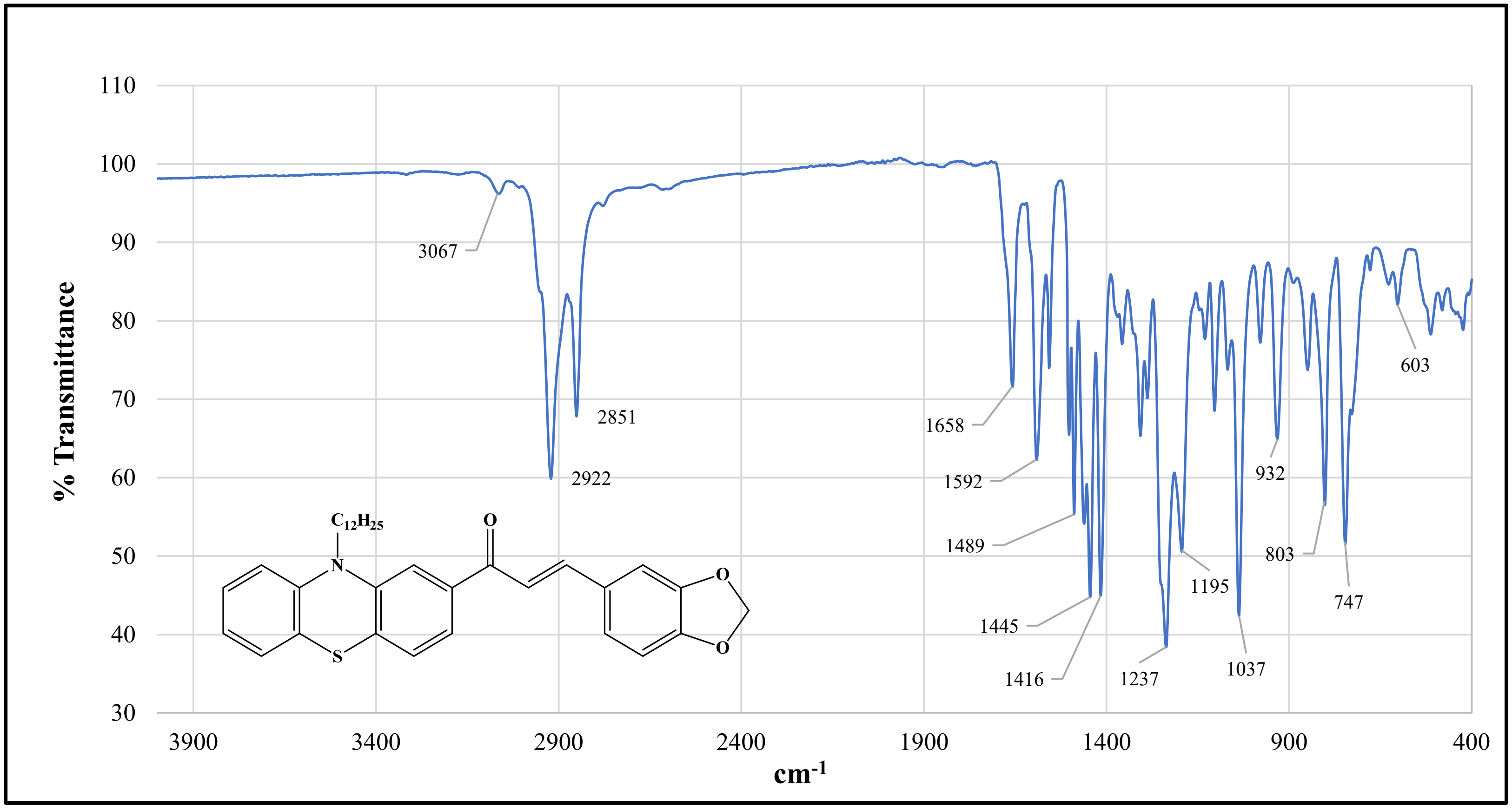

S84. **IR of (E)-3-(benzo[d][1,3]dioxol-5-yl)-1-(*10-dodecylphenothiazin-2-yl*)prop-2-en-1-one (4p).**

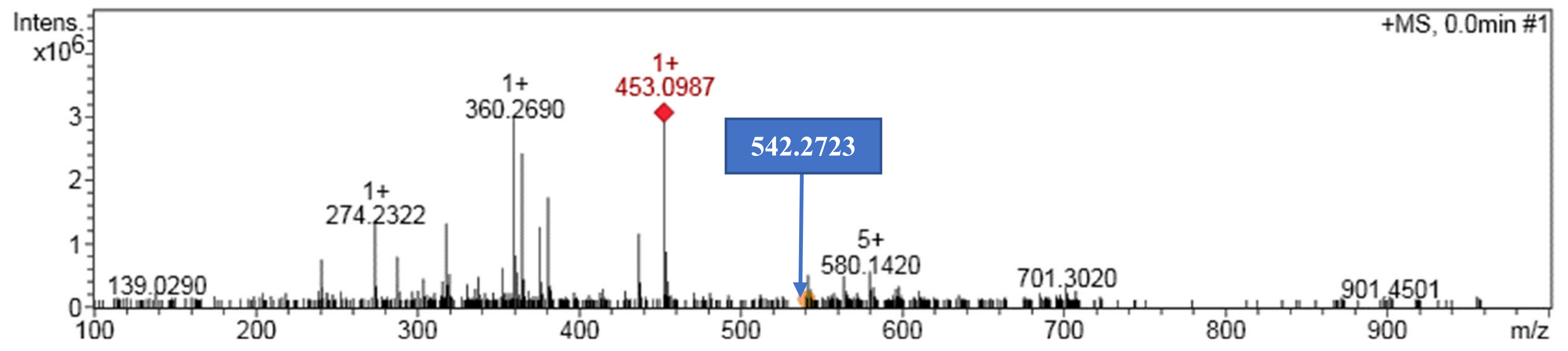

Meas. m/z	#	Ion Formula	m/z	err [ppm]	Mean err [ppm]	rdb	N-Rule	e⁻ Conf	mSigma	Std I	Std Mean m/z	Std I VarNorm	Std m/z Diff	Std Comb Dev
541.238664	1	C34H40NO3S	542.272342	1906.2	1915.5	15.5	ok	even	706.0	956.7	n.a.	n.a.	n.a.	n.a.

S85. MS of (E)-3-(benzo[d][1,3]dioxol-5-yl)-1-(*10-dodecylphenothiazin-2-yl*)prop-2-en-1-one (4p).

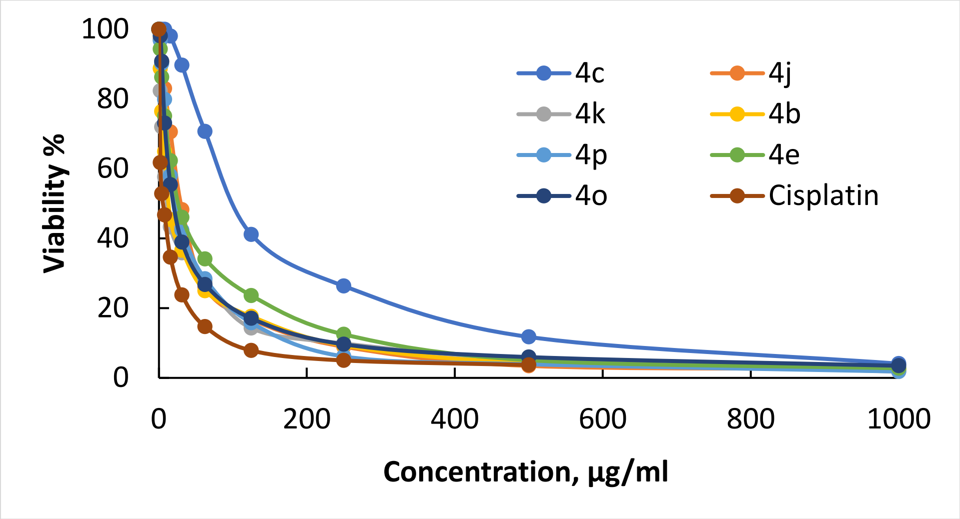

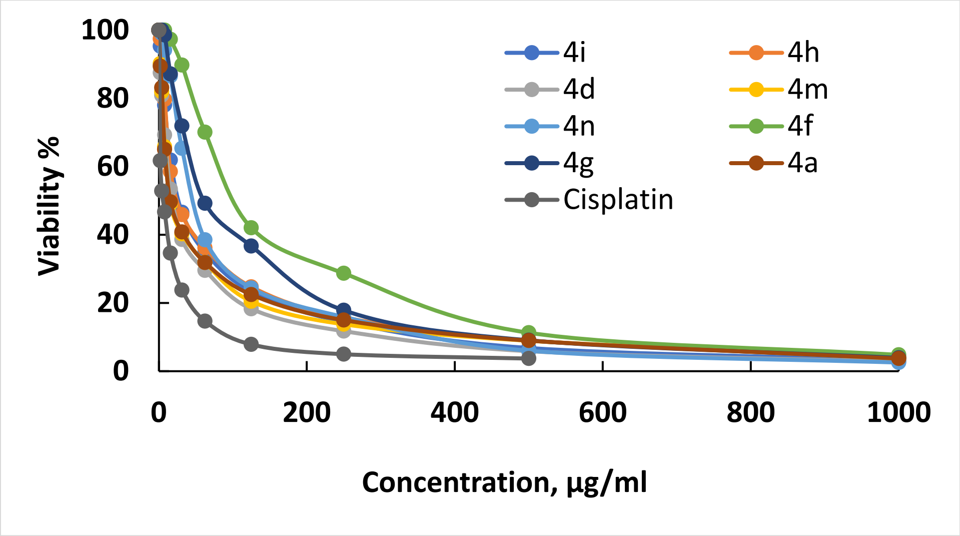

S86. The dose response curve for the in vitro antitumor activity of different compounds against human breast cancer (MCF-7) cell line.

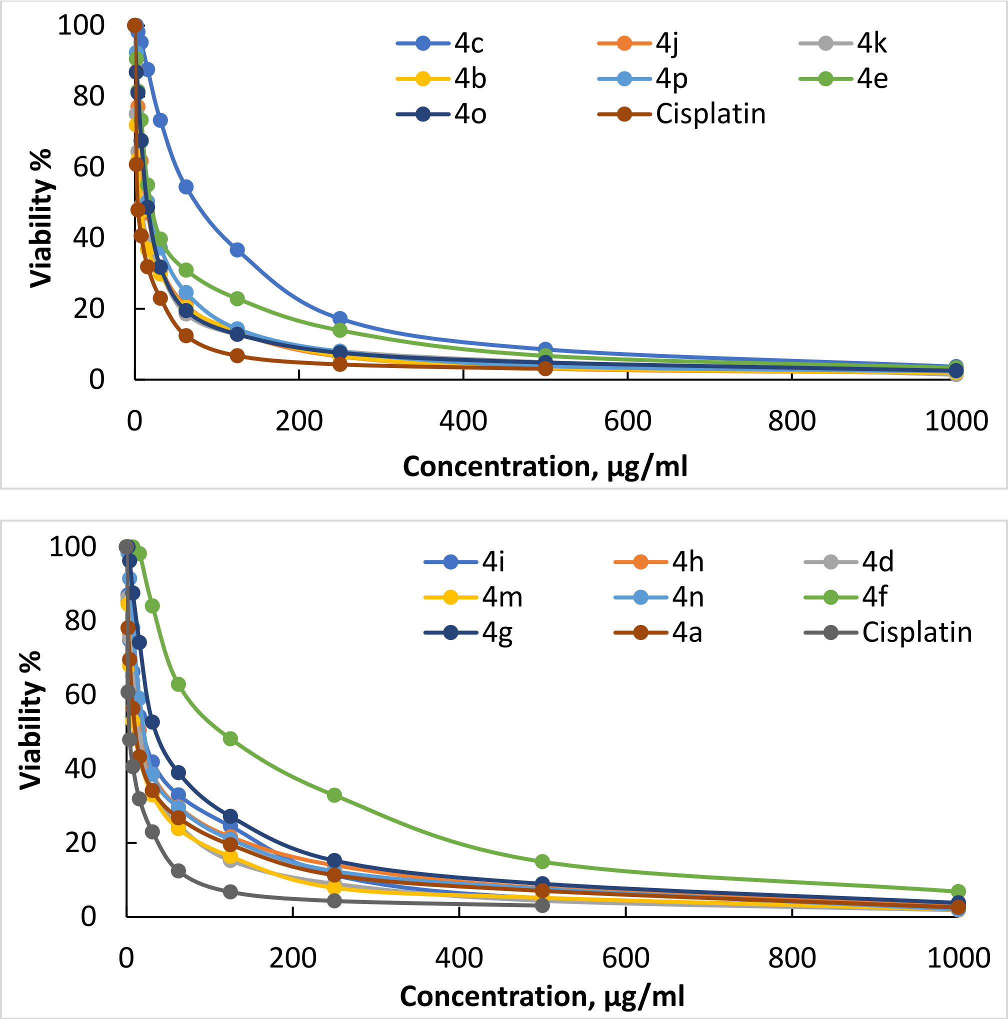

S87. The dose response curve for the in vitro antitumor activity of different compounds against human liver cancer (HepG-2) cell line.
